# Supplementary material for: PRMT5 Is a Critical Regulator of Breast Cancer Stem Cell Function via Histone Methylation and FOXP1 Expression
Source: Cell Rep. 2017 Dec 19;21(12):3498–513. doi: 10.1016/j.celrep.2017.11.096 (PMC5746596; doi:10.1016/j.celrep.2017.11.096)
Supplement: Document S1. Supplemental Experimental Procedures and Figures S1–S7 [file mmc1.pdf]

Cell Reports, Volume *21*

Supplemental Information

PRMT5 Is a Critical Regulator of Breast Cancer Stem Cell Function via Histone Methylation and FOXP1 Expression

Kelly Chiang, Agnieszka E. Zielinska, Abeer M. Shaaban, Maria Pilar Sanchez-Bailon, James Jarrold, Thomas L. Clarke, Jingxian Zhang, Adele Francis, Louise J. Jones, Sally Smith, Olena Barbash, Ernesto Guccione, Gillian Farnie, Matthew J. Smalley, and Clare C. Davies

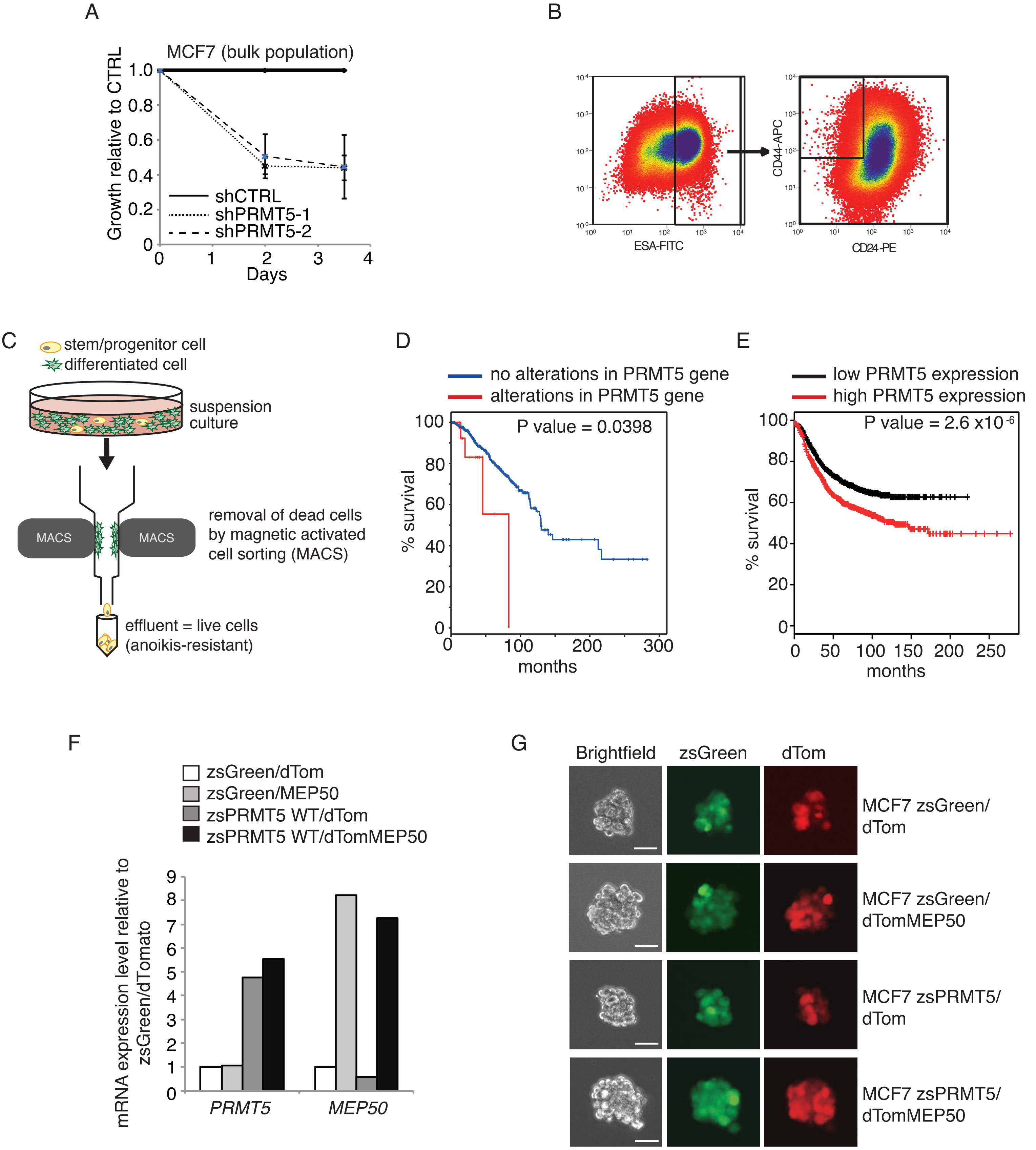

Supplemental Figure 1, related to Figure 1: PRMT5 is required for breast cancer maintenance.
(A) Proliferation of MCF7 shCTRL, shPRMT5-1 (hairpin1) or shPRMT5-2 (hairpin2) cells was assessed by MTT assay. **(B)** FACS-sorting methodology. Cells were stained with ESA-FITC, CD24-PE and CD44-APC and gated for $ESA^{+}/CD24^{low}/CD44^{+}$. **(C)** Schematic for the isolation of anoikis-resistant cells. **(D)** Kaplan-Meier (KM) plot of % survival for alterations in PRMT5. Data taken from cBioportal.org (TCGA, 2015 dataset, 816 cases). 2% of cases exhibit alterations in the PRMT5 gene; 11 amplification, 4 missense and 1 in-frame mutation. **(E)** KM plot for survival of breast cancer patients with low and high PRMT5 levels. Data taken from http://kmplot.com/analysis/ with filters set to “all” breast cancers using probe 1564520_s_at. **(F)** qPCR for PRMT5 and MEP50 gene expression in engineered MCF7 cells. (G) Microscope images of mammospheres derived from MCF7 cells engineered to overexpress PRMT5 and MEP50. Scale bar = 50µm.

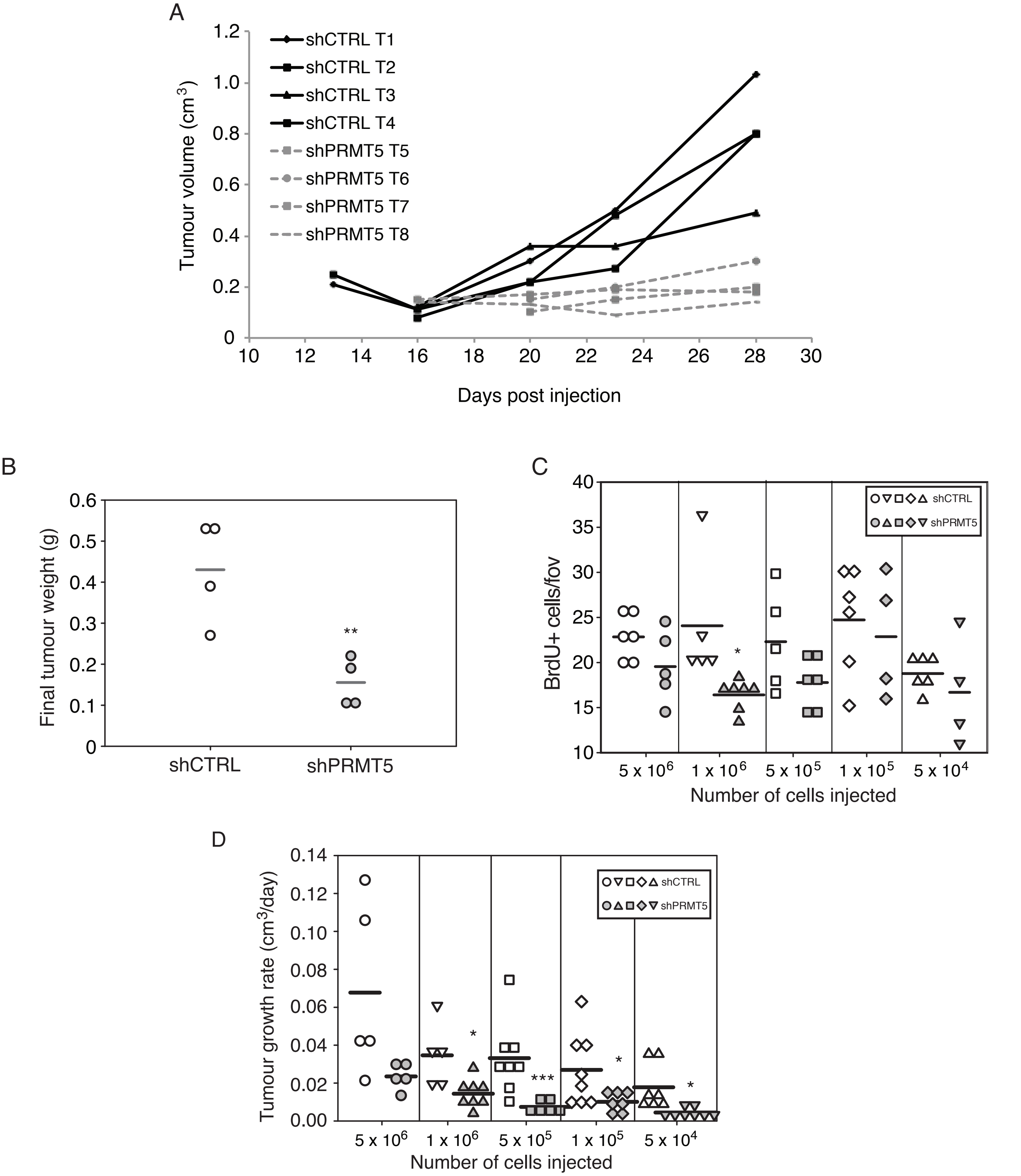

	Average tumour growth rate (cm^3/day) ± s.e.m.				
# cells injected	5 x 10^6	1 x 10^6	5 x 10^5	1 x 10^5	5 x 10^4
shCTRL	0.07 ± 0.02	0.03 ± 0.01	0.03 ± 0.01	0.03 ± 0.01	0.02 ± 0.005
shPRMT5	0.02 ± 0.003	0.01 ± 0.003	0.01 ± 0.001	0.01 ± 0.002	0.004 ± 0.001

Supplemental Figure 2, related to Figure 2: PRMT5 is required for self-renewal and tumour growth *in vivo*.
Data shown in (A) and (B) are from a specific cohort of mice (Group 3 - 5x10^5 cells) from one limiting dilution experiment as shown in Figure 2B. **(A)** Growth of tumours from mice injected with 5 x 10^5 cells was monitored by caliper measurements at the times indicated and plotted as tumour volume (cm^3) over time. T denotes an individual tumour. T1-4 denote shCTRL tumours (solid black lines). T5-8 denote shPRMT5 tumours (dotted grey lines). **(B)** Final tumour weight (g) of tumours derived from shCTRL or shPRMT5-expressing cells in (A). Bar = mean, ** $p < 0.01$ Student's *t*-test. **(C)** Dot density plot of BrdU$^+$ /total number of cells in field of view (fov) from shCTRL and shPRMT5 tumours. **(D)** Average tumour growth rate of tumours from the limiting dilution assay shown in Figure 2B.

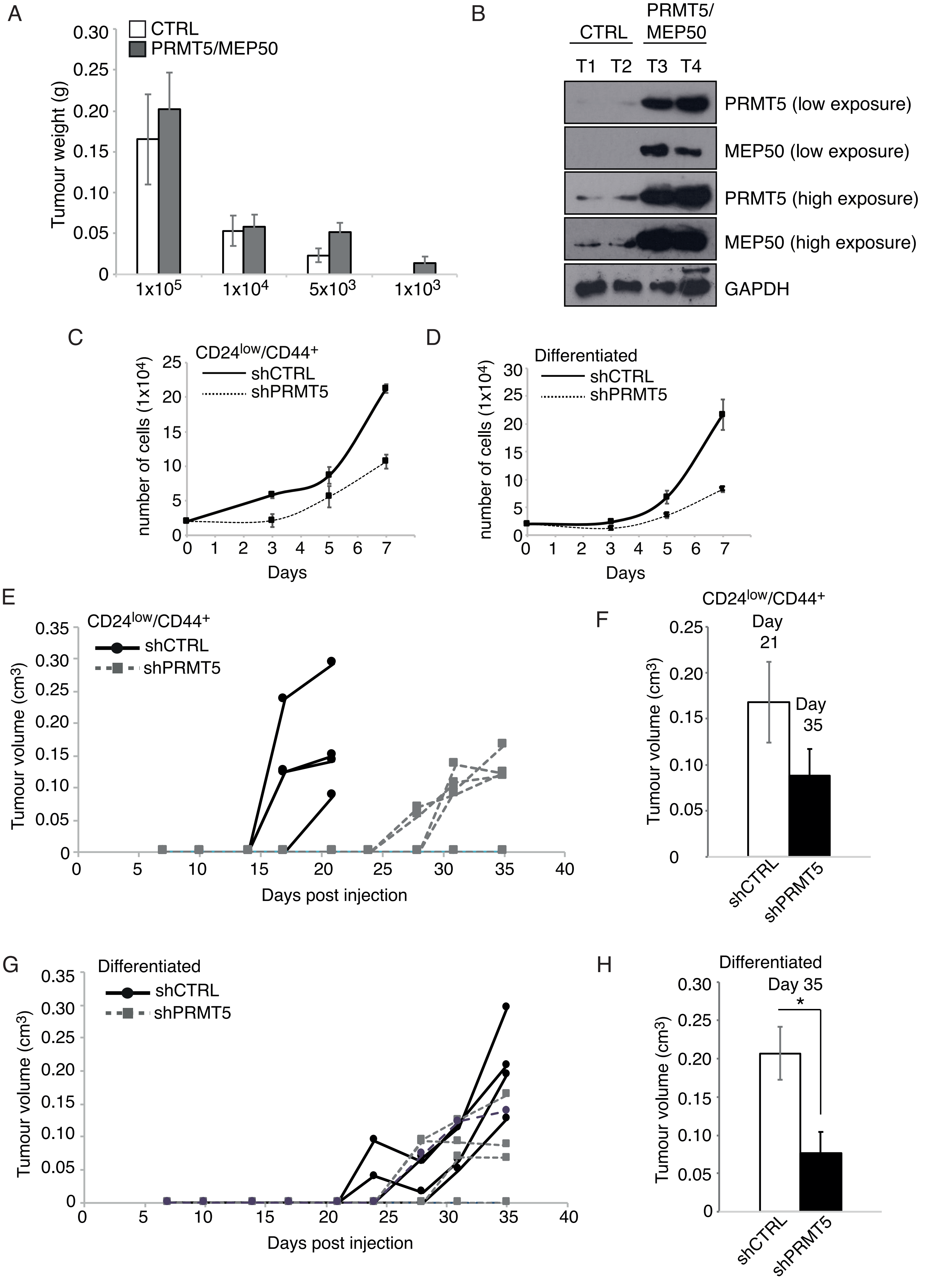

Supplemental Figure 3, related to Figure 2: PRMT5 is required for BCSC and bulk tumour function *in vivo*.
(A) Average weight of tumours derived from PRMT5/MEP50 or control cells from the limiting dilution assay shown in Figure 2G. **(B)** PRMT5 and MEP50 overexpression was assessed in tumours derived from the limiting dilution assay. T1 and T2 are denote individual control tumours and T3 and T4 denote individual tumours derived from PRMT5/MEP50 overexpressing cells. Proliferation of shCTRL or shPRMT5 **(C)** $ESA^+/CD24^{low}/CD44^+$ or **(D)** differentiated cells was determined by counting on a haemocytometer. MCF7 shCTRL or shPRMT5 $ESA^+/CD24^{low}/CD44^+$ or differentiated cells were FACS sorted and injected into NSG mice. **(E)** Growth of tumours derived from shCTRL or shPRMT5 $EESA^+/CD24^{low}/CD44^+$ cells. **(F)** Average final volume of tumours derived from shCTRL or shPRMT5 $ESA^+/CD24^{low}/CD44^+$ cells. **(G)** Growth of tumours derived from shCTRL or shPRMT5 differentiated cells. **(H)** Average final volume of tumours derived from shCTRL or shPRMT5 differentiated cells.

A

Injected cells	Slide number	Grade	Cellularity	Tubules	Pleomorphism	Mitosis	Fibrous Tissue	Necrosis (%)
MCF7 shCTRL	135/F3/L	3	high	3	2	3	min	nd
MCF7 shCTRL	135/F3/R	2	mod	3	2	2	mod	nd
MCF7 shCTRL	135/F5/P5/L	nd	nd	nd	nd	nd	nd	nd
MCF7 shCTRL	135/F5/P5/R	nd	nd	nd	nd	nd	nd	nd
MCF7 shCTRL	24/F4/R	3	high	3	3	3	min	0
MCF7 shCTRL	24/F5	3	high	3	3	3	min	30
MCF7 shCTRL	27/F1/L	3	high	3	3	3	min	25
MCF7 shCTRL	27/F1/R	3	high	3	3	3	min	20
MCF7 shCTRL	27/F2/L	3	high	3	3	3	min	40
MCF7 shCTRL	27/F2/R	3	high	3	3	3	min	60
MCF7 shCTRL	27/F3/L	3	high	3	3	3	min	5
MCF7 shCTRL	27/F3/R	3	high	3	3	3	min	25
MCF7 shCTRL	27/F4/L	3	high	3	3	3	low	0
MCF7 shCTRL	27/F4/R	3	high	3	3	3	min	10
MCF7 shCTRL	54/F3/L	3	high	3	3	3	low	65
MCF7 shCTRL	54/F3/R	3	high	3	3	3	min	0
MCF7 shCTRL	54/F4/L	3	high	3	3	3	min	45
MCF7 shPRMT5	136/F3/C/L	3	very high	3	3	3	none	20
MCF7 shPRMT5	136/F3/C/R	nd	nd	nd	nd	nd	nd	40
MCF7 shPRMT5	160/F3/L	3	mod	2	3	3	mod	0.1
MCF7 shPRMT5	160/F3/R	3	high	3	3	3	min	0.05
MCF7 shPRMT5	160/F5/L	3	mod	2	3	3	mod	0
MCF7 shPRMT5	160/F5/R	3	mod	2	3	3	mod-high	0
MCF7 shPRMT5	162/F2/L	3	high	2	3	3	mod	0
MCF7 shPRMT5	186/F2/L	3	high	3	3	3	low	10
MCF7 shPRMT5	186/F2/R	3	high	3	3	3	mod	<5
MCF7 shPRMT5	186/F3/L	3	mod	2	3	3	mod	5
MCF7 shPRMT5	186/F3/R	3	mod	2	3	3	mod	<5
MCF7 shPRMT5	186/F5/L	3	mod	2	3	3	mod	10
MCF7 shPRMT5	186/F5/R	3	mod	2	3	3	mod	40
MCF7 shPRMT5	24/F1/L	3	mod	2	3	3	mod	<5
MCF7 shPRMT5	24/F1/R	3	high	2	3	3	min	0

B

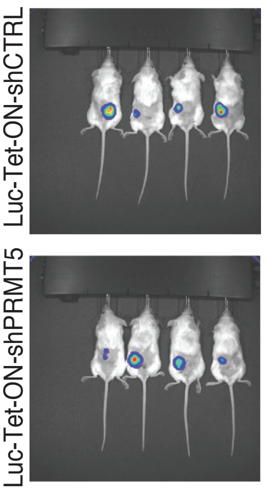

C

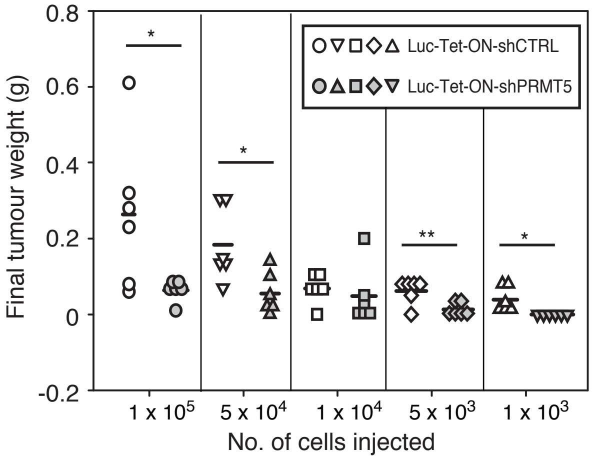

D

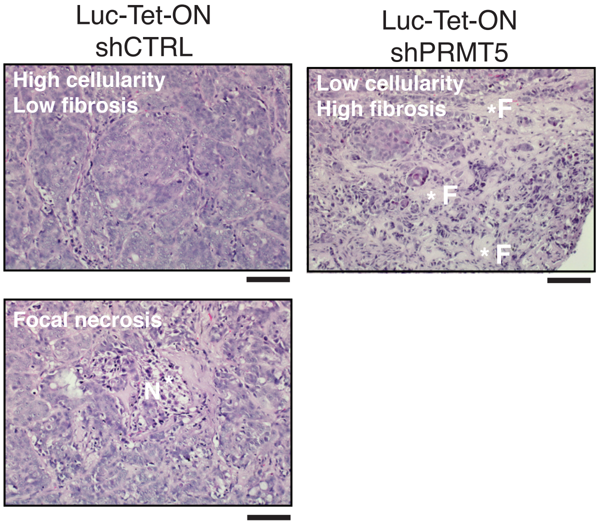

E

Injected cells	Slide number	Grade	Cellularity	Tubules	Pleomorphism	Mitosis	Fibrous Tissue	Necrosis (%)
MCF7 Luc tet-ON shCTRL	137/F2/L	3	high	3	3	3	low	0
MCF7 Luc tet-ON shCTRL	137/F2/R	3	high	3	3	2	low	0
MCF7 Luc tet-ON shCTRL	137/F6/L	3	high	3	3	2	mod	2% focal
MCF7 Luc tet-ON shCTRL	143/F1/L	3	high	3	3	2	none	0
MCF7 Luc tet-ON shCTRL	143/F1/R	3	high	3	3	3	none	0.3
MCF7 Luc tet-ON shCTRL	143/F3/L	2	mod	2	3	2	mod-high	0
MCF7 Luc tet-ON shCTRL	143/F3/R	3	high	3	3	2	min	0
MCF7 Luc tet-ON shCTRL	150/F6/L	2	mod	2	3	2	mod	0
MCF7 Luc tet-ON shCTRL	150/F6/R	3	high	3	3	3	min (focal)	0
MCF7 Luc tet-ON shCTRL	156/F5/L	3	mod	3	3	3	mod	0.05
MCF7 Luc tet-ON shCTRL	156/F5/R	3	mod	3	3	3	min	0
MCF7 Luc tet-ON shPRMT5	137/F5/L	3	mod	3	3	2	mod	0
MCF7 Luc tet-ON shPRMT5	137/F5/R	3	mod	3	3	2	mod	0
MCF7 Luc tet-ON shPRMT5	143/F2/L	1	v low	1	3	1	90% v high	0
MCF7 Luc tet-ON shPRMT5	143/F2/R	3	high	3	3	3	none	0
MCF7 Luc tet-ON shPRMT5	143/F4/R	2	mod	2	3	1	mod	0
MCF7 Luc tet-ON shPRMT5	152/F1/L	2	mod	2	3	2	mod	0
MCF7 Luc tet-ON shPRMT5	152/F2/R	2	mod	2	3	2	mod	0

Supplemental Figure 4, related to Figure 3 and 5: Depletion of PRMT5 reduces tumour aggressiveness.
(A) Table of pathological features of shCTRL and shPRMT5 tumour sections from limiting dilution assay shown in Figure 2B. **(B)** IVIS imaging to confirm tumour growth prior to *in vivo* doxycycline-induced depletion of PRMT5. **(C)** Dot density plot of final tumour weight from limiting dilution assay of MCF7 Luc-TET-ON-shCTRL or shPRMT5 cells. Bar = mean, * $p < 0.05$, ** $p < 0.01$ Student's *t*-test. **(D)** Representative images of pathological features from Luc Tet-ON-shCTRL or shPRMT5 tumours. F = fibrous tissue, N = necrotic area. Scale bar = 100µm. **(E)** Table of pathological features of MCF7 Luc-Tet-ON shCTRL and shPRMT5 tumour sections.

A

Genes suppressed by PRMT5

**ABCC11**	12.69	EDN1	3.92	S100A8	3.05	RAMP3	2.64	BDKRB2	2.43	NFKBIZ	2.19	ANKRD50	1.98
**SLC4A10**	9.91	COL4A5	3.87	ACKR3	3.02	CRISPLD1	2.63	ACACB	2.38	LGALS3BP	2.16	PALLD	1.98
**CLCA2**	8.15	COL12A1	3.83	ATF3	2.99	PIK3IP1	2.61	TFPI2	2.35	STMN3	2.16	GDF15	1.97
**MALRD1**	7.87	MAPK8IP2	3.69	ADCY5	2.99	QPRT	2.61	KIF1A	2.35	USP40	2.16	ALG10	1.97
**ABCA12**	6.48	SLC6A14	3.58	**KITLG**	2.99	GABARAPL1	2.59	**CDKN1A**	2.34	PMP22	2.15	S100A9	1.97
**COL3A1**	6.30	KYNU	3.51	METTL7A	2.97	ELF5	2.59	NTN4	2.34	DOCK10	2.14	TIMP2	1.95
CMAHP	6.22	SH3TC1	3.49	SLC12A4	2.92	MTRNR2L10	2.57	ITGB6	2.33	ANXA6	2.14	NCAM2	1.94
**COL21A1**	6.07	TIMP3	3.43	IGSF1	2.83	PHLDB2	2.56	MAP3K1	2.33	EFHD1	2.14	UGCG	1.93
**ERBB4**	5.93	COL5A1	3.43	NYNRIN	2.80	AKR1C1	2.55	ARHGAP29	2.32	FAXDC2	2.11	SEMA3E	1.93
**NPY1R**	5.89	GFRA1	3.39	SYNPO2	2.79	DBN1	2.54	LIFR	2.32	TMPRSS2	2.11	MDM2	1.93
PLXDC2	5.42	EHF	3.35	MAP1B	2.77	ELP2	2.54	ADAMTS9	2.32	PARM1	2.10	JUN	1.91
ARHGAP36	5.13	BNIPL	3.24	SESN3	2.76	CTSF	2.52	CRABP2	2.27	TMEM63C	2.08	SSFA2	1.90
CDH18	5.12	ST8SIA4	3.17	CAB39L	2.73	NGEF	2.49	ATP2B4	2.26	DBP	2.07	GNA12	1.89
OLFML3	4.96	AMIGO2	3.16	WNT5A	2.70	KIAA1407	2.47	INSR	2.26	CLSTN2	2.05	**PRICKLE2**	1.89
C16orf45	4.89	SLC39A6	3.15	FAM129A	2.69	MTRNR2L8	2.47	ETNK2	2.26	KLHL24	2.04	MARCKS	1.84
L1CAM	4.67	RGS22	3.14	KLF6	2.69	HPGD	2.45	FBLIM1	2.22	COX6C	2.04	MAN1A1	1.84
SHISA2	4.52	ACSS1	3.12	LDHB	2.68	LOC374443	2.44	PEG10	2.21	ALG10B	2.03		
PTGER4	4.28	ARMCX2	3.12	ALDH3B2	2.67	FSCN1	2.44	SYT1	2.20	PLCG2	2.01		
PDK4	4.25	SYT10	3.10	SCARA3	2.66	CLSTN3	2.43	DUSP8	2.19	IRS2	2.00		
NAALADL2	4.10	RPS18P9	3.07	PAPSS2	2.66	MTRNR2L6	2.43	MAOA	2.19	SLC7A2	2.00		

Genes activated by PRMT5

**LOX**	0.13	HSH2D	0.33	IFITM10	0.42	SLC29A3	0.48
**C1orf116**	0.15	SOX21-AS1	0.34	NOL3	0.43	FAM171B	0.49
**SCIN**	0.16	FGD3	0.35	PDK1	0.43	TSTA3	0.49
**KRT81**	0.16	**LRP4**	0.36	PLXNA2	0.43	SLC39A4	0.49
PPP1R3G	0.17	ARID5A	0.37	SLC9A2	0.43	COBLL1	0.49
**CRAT**	0.19	SLC45A3	0.37	**HK2**	0.44	HIST1H2AH	0.50
**MAB21L3**	0.21	CEACAM5	0.38	PKIB	0.44	**TPD52**	0.50
**MPPED2**	0.23	EHD3	0.39	NR5A2	0.45	STARD10	0.50
**RIMKLA**	0.25	HOXA13	0.39	ABHD17C	0.45	SPDEF	0.50
**ADM**	0.25	FUT11	0.39	GPC6	0.45	EIF5B	0.51
MUCL1	0.25	RAB3B	0.39	ITGA6	0.45	ANKRD36	0.51
**NDRG1**	0.26	MLKL	0.39	GOLM1	0.45	COX5B	0.51
SERPINB5	0.29	ALDOC	0.40	KANK1	0.46	FIGN	0.51
FAM13A	0.30	MUC5B	0.40	CA2	0.47	**FOXP1**	0.52
CAPN9	0.30	EGLN3	0.41	ZFP36L2	0.47	SNRPC	0.53
**PRMT5**	0.31	TOX3	0.41	PTPN18	0.47	PPIF	0.53
SOX21	0.32	ABLIM1	0.41	NEB	0.47	STARD7	0.54
EML5	0.33	PPFIA4	0.41	HIST1H2BB	0.48	PFN1	0.56
PRODH	0.33	GALNT12	0.41	NCAPH	0.48		
ALDH3A1	0.33	BCAS1	0.42	AGR2	0.48		

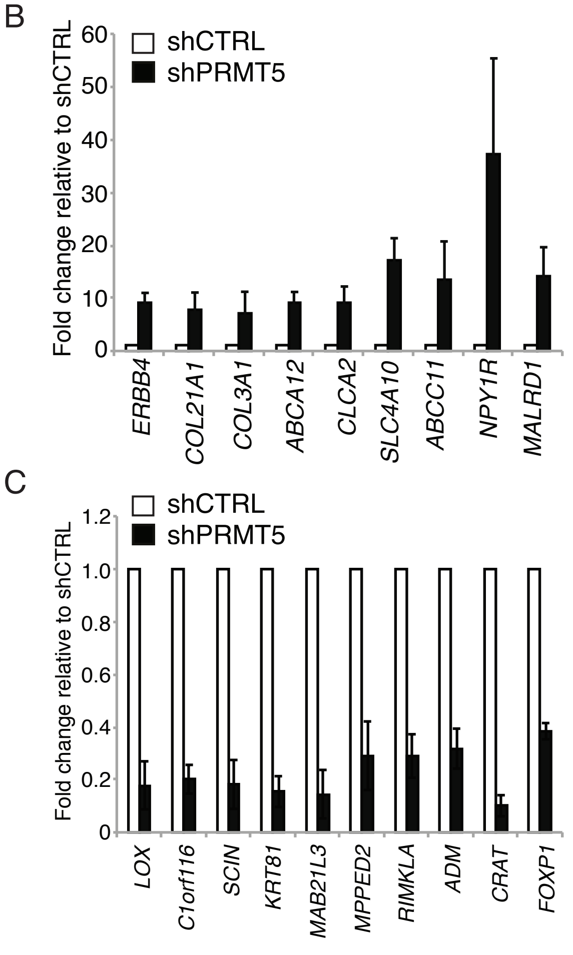

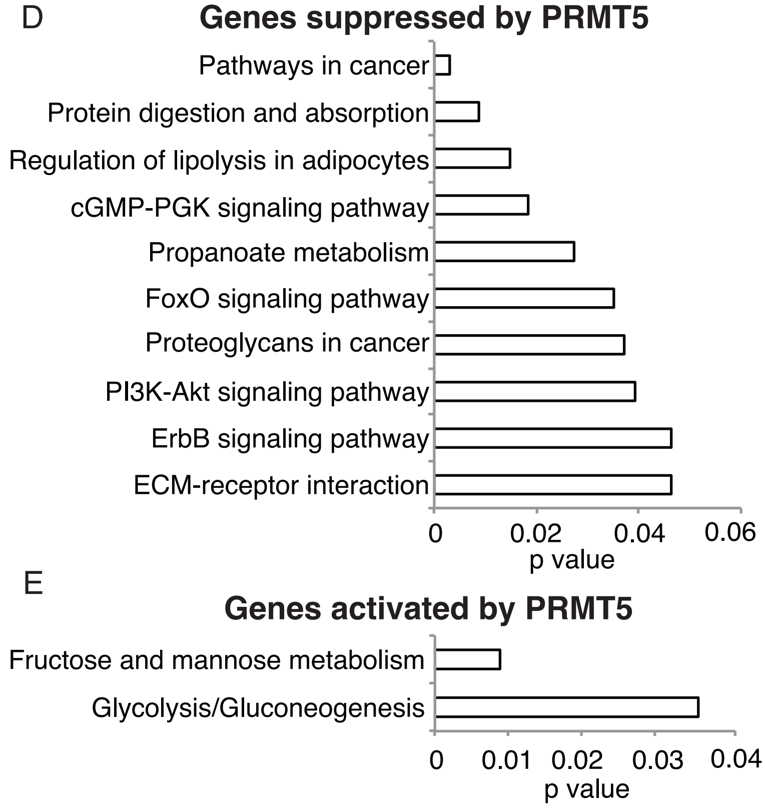

Supplemental Figure 5, related to Figure 6: PRMT5 regulates gene expression in BCSCs.
(A) Table of PRMT5-regulated genes in BCSCs. Three independent RNA samples were isolated from MCF7 shCTRL or shPRMT5 ESA^{+}/CD24^{low}/CD44^{+} cells. Number of reads for each sample are shCTRL 1: 31,997,304, shCTRL 2: 44,222,799, shCTRL 3: 13,885,650, shPRMT5 1: 39,039,672, shPRMT5 2: 132,754,382 (sample resequenced hence higher read number) and shPRMT5 3: 14,209,470. Samples were paired-end sequenced and aligned to the human hg19 genome. Differential gene expression was determined using Cuffdiff 2.2.1 with a threshold of FDR < 0.05 and fold change > 1.5. Fold change of genes that were significantly upregulated following PRMT5 knockdown (hence, suppressed by PRMT5) (red) and downregulated following PRMT5 knockdown (activated by PRMT5) (blue) are shown. Validation of top 10 differentially **(B)** PRMT5-suppressed and **(C)** PRMT5-activated genes, as highlighted in bold in (A), was performed by qPCR. *FOXP1* is not a top10 gene but is included for comparison. n=3, error = s.e.m. KEGG pathway analysis of **(D)** genes suppressed by PRMT5 and **(E)** genes activated by PRMT5.

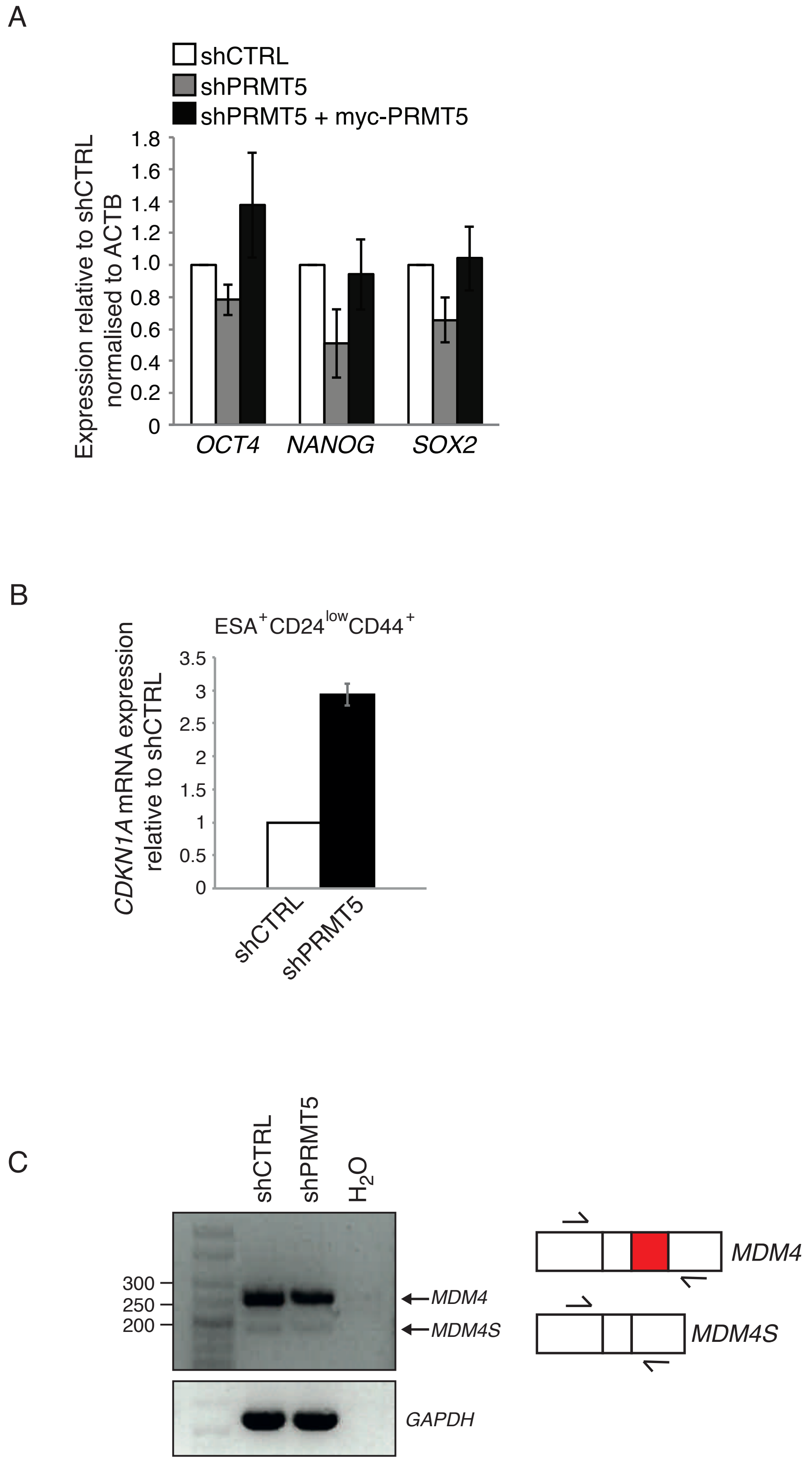

Supplemental Figure 6, related to Figure 6: Analysis of differentially regulated genes following PRMT5 depletion in BCSCs. **(A)** Quantitative PCR (qPCR) for *OCT4, NANOG* and *SOX2* in MCF7 shCTRL, shPRMT5 or shPRMT5 anoikis-resistant cells reconstituted with ectopically overexpressed PRMT5. Gene expression is expressed relative to shCTRL (n=3). **(B)** Expression of CDKN1A in ESA^{+}/CD24^{low}/CD44^{+} cells was assessed by qPCR following PRMT5 depletion (n=3). **(C)** Analysis of MDM4 splicing following PRMT5 depletion in anoikis-resistant cells. MDM4 = full length isoform, MDM4S = short isoform as a result of exon skipping.

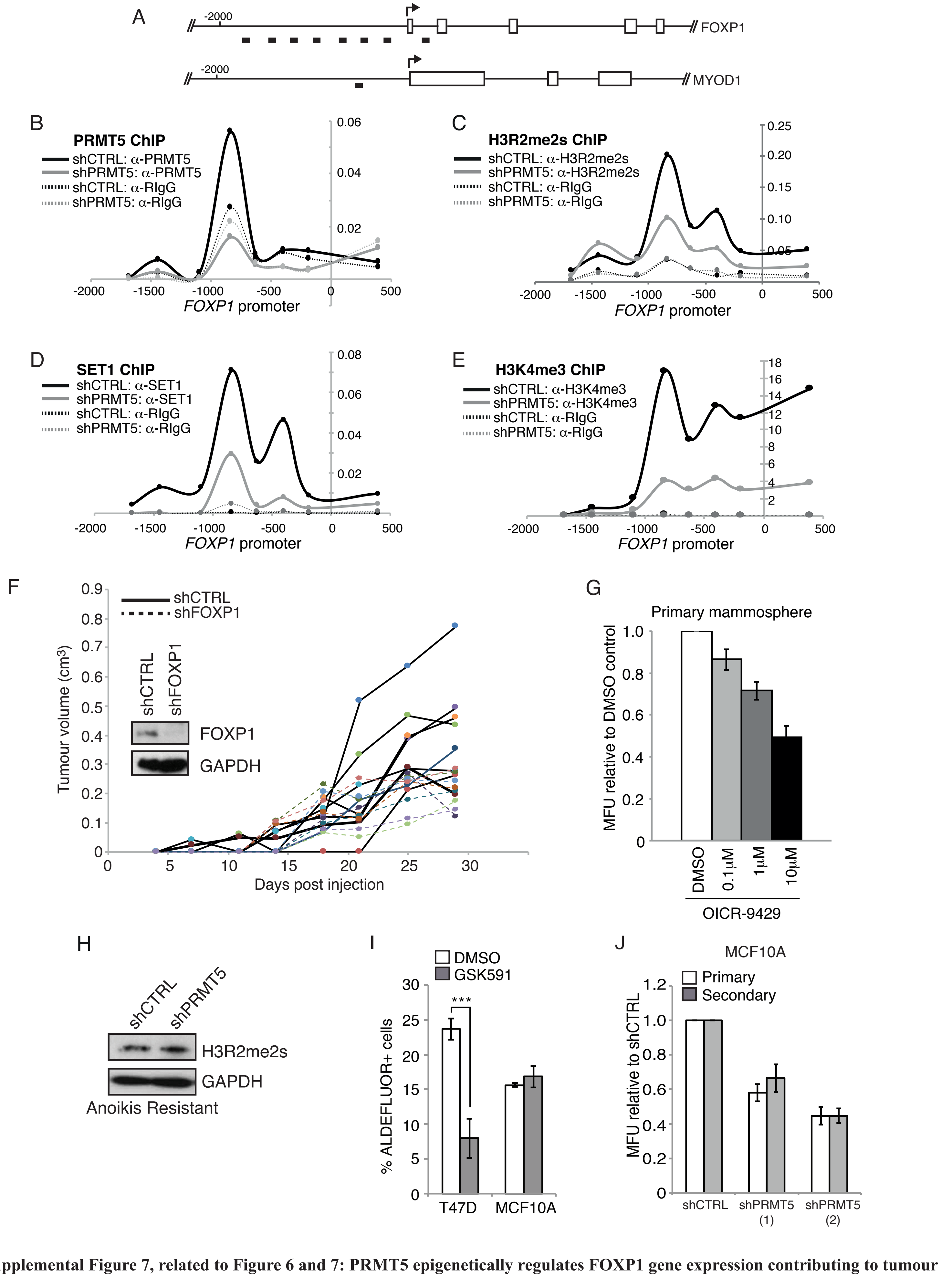

Supplemental Figure 7, related to Figure 6 and 7: PRMT5 epigenetically regulates FOXP1 gene expression contributing to tumour growth.
(A) Schematic representation of FOXP1 and MYOD1 promoter regions. Thick black bars show tiling primers used to assess enrichment at these loci. Tiling ChIP-qPCR of **(B)** PRMT5, **(C)** H3R2me2s, **(D)** SET1, **(E)** H3K4me3 enrichment across the FOXP1 promoter region. **(F)** Growth of tumours derived from shCTRL or shFOXP1 cells. FOXP1 levels in MCF7 shCTRL or shFOXP1 cells were assessed prior to injection (inset). **(G)** Mammosphere assay of MCF7 cells following WDR5 inhibition by OICR-9429. **(H)** Immunoblot for H3R2me2 levels in anoikis resistant cells. **(I)** ALDEFLUOR assay of T47D and MCF10A cells ± 5μM GSK591. **(J)** Mammosphere assay of MCF10A shCTRL or shPRMT5 cells. n=3, error = s.e.m. *** $p < 0.005$ Student's t test.

Supplementary Experimental Procedures

Cell Culture

MCF7 and T47D cells were cultured in Dulbecco's Modified Eagle's Media (DMEM) (Sigma) supplemented with 10% foetal bovine serum (FBS) (Gibco) and 5mM glutamine (Sigma). MCF10A cells were cultured in DMEM/F-12 supplemented with 25% horse serum, 500ng/ml hydrocortisone (Sigma), 10µg/ml insulin (Sigma), 20ng/ml EGF (Miltenyi) and 100ng/ml cholera toxin (Sigma). SUM159 cells were cultured in Ham's F12 Nutrient Mixture (Thermo Scientific 21765-039) supplemented with 5% FBS, 1µg/ml hydrocortisone, 5µg/ml insulin and 10mM HEPES (Sigma). All cell lines were grown in 5U/ml penicillin and 1mg/ml streptomycin.

Generation of cell lines used in this study

All RNAi-mediated depletions were conducted via lentiviral infection of mammary/breast cancer cells and the generation of stable cell lines. For lentiviral production, low passage 293T cells were transfected with pLKO-shCTRL, -shPRMT5, -shPRMT1, -shFOXP1, pHIVzsGreen, pHIVzsGreen-PRMT5 or -FOXP1, or pHIVdTomato-MEP50 and appropriate packaging vectors, using PEI reagent (Park Scientific) in a 3:1 ratio of DNA:PEI for a minimum of six hours prior to addition of fresh media. Cells were cultured for 24hr before harvesting of viral supernatant. MCF7, T47D and MCF10A cells were transduced by centrifuging with viral supernatant containing 8µg/ml polybrene (Sigma) for 90 mins at 4000rpm. Cells were selected with 1µg/ml puromycin and only low passage numbers used for experiments. For doxycycline-inducible cell lines, MCF7 cells were first transduced with p201-doubleTet lentivirus (gift from K. Brennan) and CMV-Luciferase lentiviral particles (Amsbio), selected with 1mg/ml G418 (Sigma) and 2µg/ml blasticidin (Thermo Scientific) and then

infected with pLKO-Tet-ON-shCTRL or shPRMT5. For reconstitution of wild-type PRMT5 or catalytically inactive PRMT5, cells were first transfected with pLKO-shCTRL or shPRMT5 and selected with 1µg/ml puromycin. Surviving cells were then lentivirally infected with shRNA-resistant pHIVzsGreen-PRMT5 or PRMT5 (G367A/R368A) as described above and cells expressing the vector were isolated by flow cytometry. The shRNA sequences used in this study are as follows:

shCTRL: 5'-CCTAAGGTTAAGTCGCCCTCG-3'

shPRMT1(1): 5'-CCGGCAGTACAAAGACTACAA-3'

shPRMT1(2): 5'-TGAGCGTTCCTAGGCGGTTTC-3'

shPRMT5(1): 5'-GCGTTTCAAGAGGGAGTTCAT-3'

shPRMT5(2): 5'-AGGGACTGGAATACGCTAATT-3'

shFOXP1 (SIGMA TRCN0000015664): 5'-GCAGCAAGTTAGTGGATTAAA-3'

Mammosphere assay

MCF7, T47D and MCF10A cells were trypsinised and passed through a 25G needle to generate a single cell suspension. Cells were plated onto pHEMA (Sigma) -coated 6-well plates at a density of 3000 cells/well for MCF7 and 5000 cells/well for T47D, SUM159 and MCF10A cells and cultured at 37°C for five days. For MCF7, T47D and SUM159, cells were cultured in DMEM/F12 (Thermo Scientific 21041025) supplemented with B27 (Thermo Scientific 12587010), 20ng/ml EGF (Miltenyi) and 5U/ml penicillin and 1mg/ml streptomycin. For MCF10A, cells were cultured in DMEM/F12 supplemented with B27, 500ng/ml hydrocortisone, 10µg/ml insulin, 20ng/ml EGF, 100ng/ml cholera toxin and 5U/ml penicillin and 1mg/ml streptomycin. Mammospheres > 50µm were scored using a graticle. To assess self-renewal capabilities, mammospheres were harvested and disaggregated into single cell suspension as previously. Cells were cultured in pHEMA-coated 6-well plates for a further five

days prior to scoring. For mammosphere assays from xenografts, tumours were dissociated using the Miltenyi Tumour Cell Dissociation kit according to manufacturer's instructions. Cells were plated at 5000 cells/well and scored for both primary and secondary mammospheres and expressed as mammosphere forming unit (MFU). For drug treatment, cells were incubated with 5μM GSK591 or 2.5μM 4-OHT (Sigma).

Isolation of anoikis-resistant cells

MCF7 cells were plated on poly-HEMA coated 15cm dishes at a density of 900 cells/cm^2 and cultured in suspension overnight at 37°C. Anoikis-resistant cells were isolated by depletion of dead cells using the Miltenyi Dead Cell Removal kit according to manufacturer's instructions (Miltenyi 130-090-101).

Cell sorting and flow cytometry

Cells were trypsinised to form a single cell suspension. Cells were incubated with primary antibody as detailed in the table below prior to sorting or analysis.

Antibody	Cat No	Dilution
**ESA-FITC**	130-080-301	1:10
**CD24-PE**	130-095-952	1:10
**CD44-APC**	130-095-177	1:20
**Mouse IgG1-FITC**	130-092-213	1:10
**Mouse IgG1-PE**	130-092-212	1:10
**Mouse IgG1-APC**	130-098-214	1:20

Annexin-FITC (Biolegend 640905) staining was performed according to manufacturer's instructions. For PI staining, cells were harvested and fixed with ice cold 70% ethanol for 30

mins prior to incubation with 25 μg/ml propidium iodide solution (Sigma) containing 0.1 mg/ml RNAse A.

ALDEFLUOR assay

ALDEFLUOR assay (Stem Cell Technologies) was performed according to manufacturer's instructions using 2 x 10^5 cells per sample and incubation for 30 mins at 37^oC. Where appropriate, cells were incubated with 5μM GSK591 or DMSO for a minimum of 24hr prior to harvest.

Xenograft studies and *in vivo* imaging

Animal experiments were conducted in accordance with United Kingdom Home Office regulations. For the limiting dilution assay, 5-7 week old female NSG (NOD/Scid/IL-2Rγnull) mice were injected with the appropriate number of MCF7 shCTRL, shPRMT5, shFOXP1, zsGreen/dTomato or zsPRMT5/MEP50 cells cells in a 1:1 ratio with matrigel (200μl total volume) (BD Biosciences). At the time of injection, a slow release oestrogen pellet (Innovative Research of America, NE-121) was subcutaneously implanted at the base of the tail. Tumour growth was monitored by caliper measurements. Two hours prior to harvest at the experimental endpoint, mice were injected with 100mg/kg BrdU (Sigma). Extracted tumours were weighed and imaged. Tumours were subsequently processed for protein extraction, immunohistochemistry (IHC) and mammosphere assay where appropriate. For *in vivo* depletion of PRMT5, female NSG mice were injected with 5 x 10^6 MCF7 Luc-Tet-ON-shCTRL or shPRMT5 cells as above. IVIS imaging and doxycycline-inducible knockdown initiated when tumours were of a palpable size. Mice were then maintained on diet consisting of doxycycline chow (TestDiet, T-5BQ8-1816629-203). For imaging, mice were injected IP with 150μg/g of luciferin (Promega) 15 mins prior to imaging. Images were captured on an

IVIS Spectrum. Tumours were harvested at the experimental endpoint and weighed prior to dissociation into single cells using the Miltenyi tumour dissociation kit according to manufacturer's instructions. Cells were then injected into female NSG mice in a limiting dilution assay.

Immunoblotting

Cell lysates were prepared by sonicating in lysis buffer (Tris-HCl pH 8.0, 150mM NaCl, 50mM, 1mM EDTA, 1% NP40, 1mM Na_3VO_4, 50mM NaF, 1mM β-glycero-phosphate, 100mM phenylmethylsulfonyl fluoride (PMSF), 10μg/ml leupeptin, 10μg/ml aprotinin), prior to incubation on ice and clarification by centrifugation. Proteins were separated by SDS-PAGE and transferred to nitrocellulose or PVDF. Membranes were blocked and incubated overnight in primary antibody at the dilutions indicated below. Membranes were washed and incubated with secondary antibody as indicated below.

Antibody	Company	Catalogue number	Dilution
**PRMT1**	CST	2449	1:1000
**PRMT5**	Millipore	07-405	1:1000
**MEP50**	CST	2823	1:1000
**SDMA**	CST	13222	1:1000
**SmD3**	Sigma-Aldrich	HPA001170	1:100
**FOXP1**	Kind gift from A. Banham		1:30
**GAPDH**	Santa Cruz	sc-32233	1:5000
**ACT**	Sigma-Aldrich	A2228	1:5000
**Tubulin**	Sigma-Aldrich	T6199	1:1000

**Anti-Rabbit IgG HRP**	DAKO	P0399	1:5000
**Anti-Mouse IgG HRP**	DAKO	P0447	1:5000

Immunohistochemistry

Tumours were fixed overnight in 10% neutralised buffered formalin (NBF) prior to embedding in paraffin. Sections were stained for BrdU (DAKO, M0744, 1:200) using the Mouse on Mouse (M.O.M. ™) Basic kit (Vector Labs) according to manufacturer's instructions and with haematoxylin (Sigma) and eosin (Sigma).

RNA extraction, qPCR and RT-PCR

RNA was extracted from cell pellets using the GenElute Mammalian Total RNA Isolation Kit (Sigma) according to manufacturer's instructions. RNA was reverse transcribed using the SensiFAST™ cDNA Synthesis Kit (Bioline) according to manufacturer's instructions. qPCR was performed using SYBR® Green PCR Master Mix (Thermo Scientific) according to manufacturer's instructions. RT-PCR for MDM4 was performed under the following conditions: 95°C: 5mins, followed by 95°C: 45s, 58°C: 30s, 72°C: 40s (43 cycles for MDM4 and 40 cycles for GAPDH) and 74°C: 4mins followed by cooling to 4°C. Water was used as a negative control. PCR products were visualised on a 3.5% agarose gel containing ethidium bromide. Primers used in this study are described below.

Gene	Forward primer (5’-3’)	Reverse Primer (5’-3’)
ACTB	CTCTTCCAGCCTTCCTTCCT	GGATGTCCACGTCACACTTC
PRMT5	GGCCAAGCAGGGGTTTGA	GGGCCGATTCTTAGCAGGTT
MEP50	CTCACTTGGGTTGGGGAGAG	CTCATTCTCATCTAGTTCCCACA
FOXP1	CCCTTCCCCTTCAACCTCTT	TTGCCTGTGGTTTCTTCTGC
OCT4	CGTGAAGCTGGAGAAGGAGA	TGCTCGAGTTCTTTCTGCAG
NANOG	CGTGAAGCTGGAGAAGGAGA	TGCTCGAGTTCTTTCTGCAG
SOX2	CCCCTGTGGTTACCTCTTCC	CCGGGGAGATACATGCTGAT
CRAT	CAGAAGATGGCTCCTGTGGG	AGCTCGGGTTTCTTCGTGTA
LOX	ACCCAGCCGACCAAGATATT	CTTCAGCCACTCTCCTCTGG
C1orf116	TTTCCGAGCACTGCCCATAA	TGCTGAGTGATGGTCTCCTC
SCIN	CGTGGAAGAAGGAAGTGAACC	TCATCATCACCTCCATCTGG
KRT81	CATGATCCAAAGGCTGACGG	CATCACTGAGGGCCGCCT
MAB21L3	GGAAGCTGGTGGAAAATGCA	TGTTTCCACAGCAACCCAAA
MPPED2	CTTCTCCACACAGGCGATTT	TGATTCCCAGCAATCACTATTTTA
RIMKLA	ACTGATGGACGGATGCAGAG	CCAACTGCTTGCCTTGTTCT
ADM	CGGCCCCAGGACATGAAG	TAGCGCTTGACTCGGATGC
CDKN1A	GAAGAAGGGCACCCTAGTTCTAC	ACCACATGGGACCCTCAC
ERBB4	CATCGAGAATGTGCTGGAGG	GGGACACTGAGTAACACATGC
COL21A1	GGGAAGTGATGAAGCAGAAACT	ATCCCCTTTCATCACGAGCT
COL3A1	CCCCCTGGAATCTGTGAATC	GCGAGTCCTCCTACTGCTAC
ABCA12	CTCGCACGAATTCTTGGCTT	GCTGTCACATAGTTCTCTTCGT
CLCA2	ACTTAACAGCTGGCTACGGA	CTCATCGAACACACCCCAAC

SLC4A10	AGAGGATGGAAGGGAGTCAC	TTCCTCGTCATCATCCTCGG
ABCC11	AGAACACCATCCCTCCACTG	GCTTTTTCAATCCCTCGCCT
NPY1R	AGGAGAAATACCAGCGGATCT	TTCCCTTGAACTGAACAATCC
MALRD1	AGGTATCAGAACAAGGGACCTG	ATTTCCGGACAAAGCTCAGA
MDM4	TGTGGTGGAGATCTTTTGGG	GCAGTGTGGGGATATCGT
GAPDH	GAAGGTGAAGGTCGGAGTC	GAAGATGGTGATGGGATTTC

RNA-sequencing

ESA^{+}CD24^{low}CD44^{+} cells were isolated by flow cytometry from MCF7 shCTRL or shPRMT5 cells. Total RNA was extracted using the GenElute Mammalian Total RNA Isolation Kit and rRNA was depleted using the Low Input RiboMinus™ Eukaryote System v2 (Thermo Scientific) prior to sequencing on the Illumina Hiseq. Sequenced reads were mapped to build hg19 of the human genome from the University of California Santa Cruz (UCSC) genome database using TopHat version 2.0.12 (Kim et al., 2013) and aligned with Bowtie (Langmead et al., 2009) using default parameters and by supplying the gene model with annotations from Ensembl. Differential gene expression was determined using Cuffdiff 2.2.1 with a threshold of FDR < 0.05 and fold change > 1.5. Enriched Gene Ontology terms and KEGG pathways were identified using DAVID (david.ncifcrf.gov) (Huang et al., 2009). Experiment was performed using three independent replicates of shCTRL and shPRMT5 ESA^{+}CD24^{low}CD44^{+} cells.

Isolation of tumour-derived patient cells

Fresh tumour samples were collected from resected breast tumours from patients at the Queen Elizabeth Hospital, Birmingham or in collaboration with Barts Cancer Institute with informed consent. Samples were chopped into 0.5cm^3 chunks and dissociated by incubating overnight

with 0.5mg/ml collagenase (Sigma-Aldrich) and 0.5mg/ml hyaluronidase (Sigma) in RPMI supplemented with 5% FBS, 5μg/ml amphotericin (Sigma) and 5U/ml penicillin and 1mg/ml streptomycin. Cells were isolated by centrifugation and incubated in red blood cell lysis buffer (155mM NH_4Cl, 10mM $KHCO_3$, 0.1mM EDTA) at room temperature for 10 min. Cells were collected by centrifugation at 800rpm for 10 min. For mammosphere assays, 10 000 cells/well of a poly-HEMA coated 6-well plate were cultured ± 5μM GSK591 or DMSO for the duration of the assay.

Chromatin Immunoprecipitation

MCF7 shCTRL or shPRMT5 cells were fixed in 1% formaldehyde before being quenched with 0.125M glycine. Cells were washed once with PBS and nuclei isolated by incubating with lysis buffer (10mM Tris-HCl pH 8.0, 10mM NaCl, 0.2% NP40, 10mM sodium butyrate, 50μg/ml PMSF, 1μg/ml leupeptin) and centrifugation. Nuclei were lysed (50mM Tris-HCl pH 8.1, 10mM EDTA, 1% SDS, 10mM sodium butyrate, 50μg/ml PMSF, 1μg/ml leupeptin) and chromatin sonicated to produce DNA fragments of between 300bp and 500bp. Chromatin was precleared using Rabbit IgG (Millipore, 12-370; 1 μg per 10^6 cells) and protein G-agarose (Roche, 11719416001). ChIP was performed using chromatin equivalent to 5 x 10^6 cells. Chromatin was incubated overnight at 4^oC with 5μg PRMT5 (Millipore, 07-405), H3K4me3 (Abcam, ab8580), Set1 (Abcam ab70378), H4R3me2s (Abcam ab5823), 10μl H3R2me2s (gift from E. Guccione) or the equivalent amount of Rabbit IgG (Millipore, 12-370), followed by 3hr incubation with protein G beads. Beads were collected and washed twice with wash buffer 1 (20 mM Tris-HCl pH 8.1, 50 mM NaCl, 2 mM EDTA, 1% Triton X-100, 0.1% SDS), once with wash buffer 2 (10mM Tris-HCL pH 8.1, 250mM LiCl, 1mM EDTA, 1% NP-40, 1% deoxycholic acid), twice with Tris-EDTA (pH 8) and eluted in 100mM $NaHC0_3$, 1% SDS. Immunoprecipitated DNA was reverse crosslinked and treated with proteinase-K prior to

purification by phenol/chloroform and ethanol precipitation. For anoikis-resistant cells, ChIP was performed using chromatin equivalent to 2 x 10^5 cells. Chromatin was incubated overnight at 4°C with 1µg H3K4me3 (Abcam, ab8580) or Rabbit IgG (Millipore, 12-370) and isolation of DNA as described earlier. Associated DNA was quantified using the primers below.

Gene	Forward primer (5'-3')	Reverse Primer (5'-3')
FOXP1 **(-1683)**	ccttagcagcctccctttct	gggtggcataagactatcgc
FOXP1 **(-1442)**	gacagtaagcctctcccctc	tcccaaaccagcatttccag
FOXP1 **(-1098)**	aggctcttagtttattccttcga	gctctcccagtgatcagtca
FOXP1 **(-838)**	agtggctctgacaagggg	ttctcccctgtttctcggaa
FOXP1 **(-627)**	gacgggagagagggagaaag	gtgacagttgaaggagagcg
FOXP1 **(-397)**	ccccatttcccatctcctga	agggatctggcaggaaagac
FOXP1 **(-187)**	cccattctttggcaattgcg	gtccctgacgtcacatccac
FOXP1 **(+396)**	caccgagagaccatttgcag	gcttgggaaaaagtggagca
MYOD1 **(-585)**	cacttcaactctcggggtct	ccgaaagaggctgagagaca

Cell proliferation assay

Cells were plated in triplicate at a density of 20 000 cells/well in 24-well plates. Cell proliferation was determined by counting on a haemocytometer with trypan blue exclusion.
