# Supplementary material for: PRMT5 Is a Critical Regulator of Breast Cancer Stem Cell Function via Histone Methylation and FOXP1 Expression
Source: Cell Rep. 2017 Dec 19;21(12):3498–513. doi: 10.1016/j.celrep.2017.11.096 (PMC5746596; doi:10.1016/j.celrep.2017.11.096)
Supplement: Document S2. Article plus Supplemental Information [file mmc2.pdf]

Cell Reports

Article

PRMT5 Is a Critical Regulator of Breast Cancer Stem Cell Function via Histone Methylation and FOXP1 Expression

Graphical Abstract

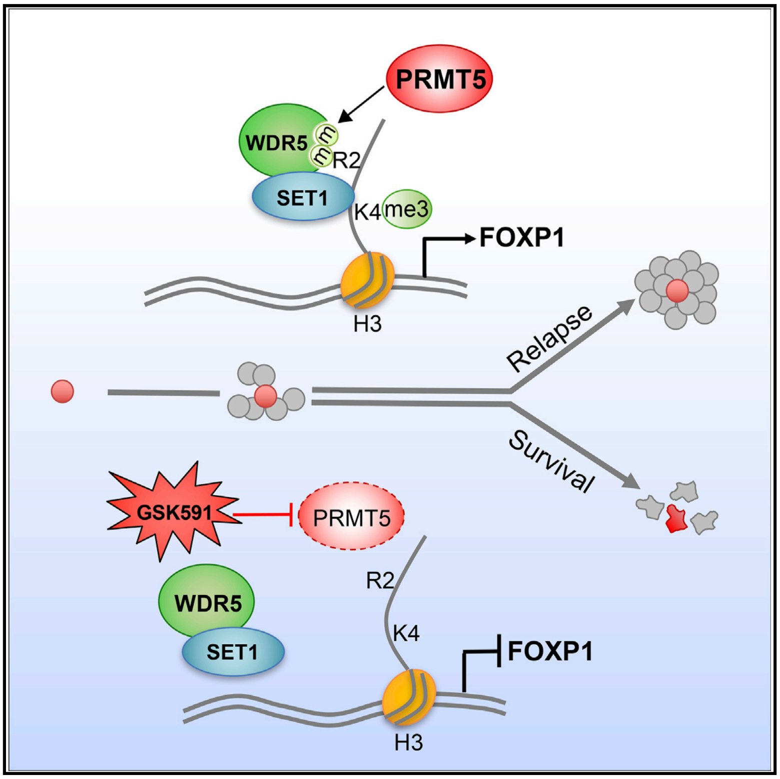

Authors

Kelly Chiang, Agnieszka E. Zielinska, Abeer M. Shaaban, ..., Gillian Farnie, Matthew J. Smalley, Clare C. Davies

Correspondence

c.c.davies@bham.ac.uk

In Brief

Chiang et al. show that the arginine methyltransferase PRMT5 contributes to breast cancer stem cell function, in part through histone methylation regulating FOXP1 expression. Targeting of PRMT5 through depletion or inhibition reduces stem cell frequency *in vitro* and *in vivo*, implicating PRMT5 as important in breast cancer pathogenesis.

Highlights

- PRMT5 expression is elevated in breast cancer stem cells (BCSCs)
- PRMT5 is required for BCSC function and self-renewal *in vitro* and *in vivo*
- Targeting PRMT5 in an established tumor reduces stem cell numbers and tumor growth
- FOXP1 promoter is methylated by PRMT5 and is an effector of PRMT5-driven BCSC function

Data and Software Availability

GSE107762

Chiang et al., 2017, Cell Reports *21*, 3498–3513
December 19, 2017 © 2017 The Authors.
https://doi.org/10.1016/j.celrep.2017.11.096

OPEN ACCESS
CellPress

Cell Reports
Article

PRMT5 Is a Critical Regulator of Breast Cancer Stem Cell Function via Histone Methylation and FOXP1 Expression

Kelly Chiang,[1] Agnieszka E. Zielinska,[1] Abeer M. Shaaban,[2] Maria Pilar Sanchez-Bailon,[1] James Jarrold,[1] Thomas L. Clarke,[1] Jingxian Zhang,[6] Adele Francis,[2,9] Louise J. Jones,[4] Sally Smith,[4] Olena Barbash,[5] Ernesto Guccione,[6,7] Gillian Farnie,[3] Matthew J. Smalley,[8] and Clare C. Davies[1,10,*]
[1]Institute of Cancer and Genomic Sciences, College of Medical and Dental Sciences, University of Birmingham, Birmingham B15 2TT, UK
[2]Department of Cellular Pathology, Queen Elizabeth Hospital Birmingham, and Institute of Cancer and Genomic Sciences, University of Birmingham, Birmingham B15 2GW, UK
[3]Structural Genomics Consortium, Botnar Research Centre, NDORMS, University of Oxford, Oxford OX3 7LD, UK
[4]Centre for Tumour Biology, Barts Cancer Institute, A Cancer Research UK Centre of Excellence, Queen Mary University of London, John Vane Science Centre, London EC1M 6BQ, UK
[5]Cancer Epigenetics DPU, GlaxoSmithKline, Collegeville, PA 19426, USA
[6]Institute of Molecular and Cell Biology (IMCB), A*STAR (Agency for Science, Technology and Research), 61 Biopolis Drive, Proteos Building #3-06, 138673 Singapore, Singapore
[7]Department of Oncological Sciences and Pharmacological Sciences, Tisch Cancer Institute, Icahn School of Medicine at Mount Sinai, New York, NY, USA
[8]European Cancer Stem Cell Research Institute, Cardiff School of Biosciences, Cardiff University, Cardiff CF24 4HQ, UK
[9]Deceased
[10]Lead Contact
*Correspondence: c.c.davies@bham.ac.uk
https://doi.org/10.1016/j.celrep.2017.11.096

SUMMARY

Breast cancer progression, treatment resistance, and relapse are thought to originate from a small population of tumor cells, breast cancer stem cells (BCSCs). Identification of factors critical for BCSC function is therefore vital for the development of therapies. Here, we identify the arginine methyltransferase PRMT5 as a key *in vitro* and *in vivo* regulator of BCSC proliferation and self-renewal and establish FOXP1, a winged helix/forkhead transcription factor, as a critical effector of PRMT5-induced BCSC function. Mechanistically, PRMT5 recruitment to the *FOXP1* promoter facilitates H3R2me2s, SET1 recruitment, H3K4me3, and gene expression. Our findings are clinically significant, as PRMT5 depletion within established tumor xenografts or treatment of patient-derived BCSCs with a pre-clinical PRMT5 inhibitor substantially reduces BCSC numbers. Together, our findings highlight the importance of PRMT5 in BCSC maintenance and suggest that small-molecule inhibitors of PRMT5 or downstream targets could be an effective strategy eliminating this cancer-causing population.

INTRODUCTION

Despite advances in early screening strategies and development of treatments tailored to specific molecular subtypes, breast cancer is still a disease associated with significant morbidity and mortality. A major contributing factor is resistance to first-line therapy and disease recurrence coupled with metastatic dissemination. Emerging research suggests that the main phenomenon contributing to this is the presence of "tumor-initiating cells," a small population of cells in the bulk tumor mass that is thought to be responsible for tumor initiation, maintenance, heterogeneity, and drug resistance. Because these cells share many biological traits with normal stem cells, they are often referred to as breast cancer stem cells (BCSCs). BCSCs can asymmetrically divide, producing one stem cell (self-renewal) and one daughter progenitor cell, which is the root of the hierarchy that defines cancer heterogeneity. Importantly, these cells have slow rates of division, high expression of drug efflux pumps, and high capacity for DNA repair (Holohan et al., 2013) and are thus relatively drug resistant. Therefore, although BCSCs represent only a small proportion of the bulk tumor, they are of major clinical importance. Given the failure of current treatments, identifying factors that are critical for BCSC function is vital for the development of novel therapies.

In the breast, cancer stem cells (CSCs) are molecularly defined as $ESA^{+}CD24^{low}CD44^{+}$ (Al-Hajj et al., 2003; Fillmore and Kuperwasser, 2008). Transplantation of as few as 1,000 patient-derived $ESA^{+}CD24^{low}CD44^{+}$ cells enabled reconstitution of a heterogeneous tumor phenotypically resembling the original tumor. Moreover, $ESA^{+}CD24^{low}CD44^{+}$ cells isolated from xenografts could undergo multiple rounds of serial transfer in mice (Al-Hajj et al., 2003). Thus, the $ESA^{+}CD24^{low}CD44^{+}$ lineage has considerable proliferative and repopulating potential and has been extensively used to isolate cells with increased tumorigenicity, defining the breast tumor-initiating population. This phenomenon can also be recapitulated *in vitro*; culturing of patient-derived or breast cancer cell lines in suspension as

This is an open access article under the CC BY license (http://creativecommons.org/licenses/by/4.0/).

OPEN ACCESS
CellPress

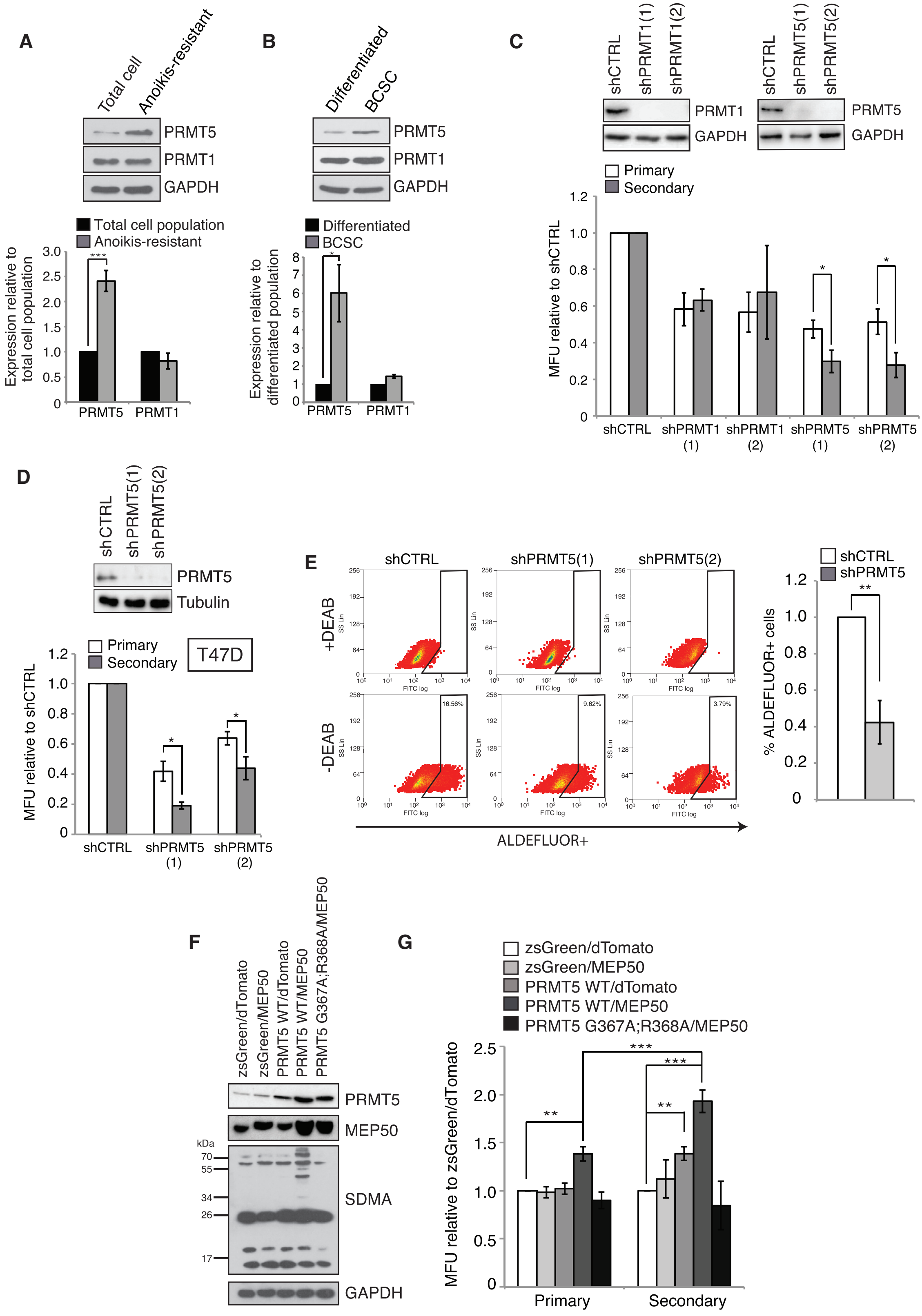

(legend on next page)

mammospheres enriches for tumor-initiating cells that generate *in vivo* tumor growth 1,000 times more potently than monolayer-derived cells (Ponti et al., 2005).

Post-translational modification of histone tails leading to changes in chromatin composition and configuration is a principal component of epigenetic-mediated gene expression. Recently, there has been a growing appreciation that histone-modifying enzymes are responsible for promoting gene expression in CSCs that facilitates cellular plasticity between cancer and non-cancer stem cell-like phenotypes (Feinberg et al., 2016; Harrison et al., 2010; Muñoz et al., 2012). This is also true in the breast, in which deregulated histone lysine methylation contributes to BCSC function and aggressive disease (Chang et al., 2011; Wu et al., 2013). In contrast, very little is understood about the contribution of arginine methylation. Protein arginine methyltransferases (PRMTs) catalyze mono- and dimethylation of the guanidino group of the arginine residue using S-adenosyl methionine (SAM) as a methyl donor. Dimethylation can occur asymmetrically (asymmetric dimethylarginine [ADMA]), with two methyl groups placed onto one of the terminal nitrogen atoms of the guanidino group, or symmetrically (symmetric dimethylarginine [SDMA]), whereby one methyl group is placed onto each of the terminal nitrogen atoms. Recently PRMT5, the main symmetric arginine dimethyltransferase in mammalian cells, has been increasingly associated with stemness. PRMT5 maintains embryonic stem cell (ESC) pluripotency by upregulating NANOG and OCT4 expression (Tee et al., 2010), promotes somatic cell reprogramming (Goyal et al., 2013; Nagamatsu et al., 2011), and is required for the homeostasis of adult stem cells (Liu et al., 2015; Zhang et al., 2015). Notably, PRMT5 can drive or repress gene expression according to the modified histone residue; histone H3R2me2s drives H3K4me3 and gene expression, while methylation of H2AR3, H4R3, and H4R8 represses gene activation (Di Lorenzo and Bedford, 2011; Migliori et al., 2012). Given the parallels between normal stem cell function, somatic cell reprogramming, and CSCs, these findings imply that PRMT5 may be an important regulator of CSCs. Indeed, PRMT5 has been shown to contribute to leukemic and glioblastoma stem cell function (Banasavadi-Siddegowda et al., 2017; Jin et al., 2016). Regarding the breast, very few studies have addressed the potential pro-tumorigenic role of PRMT5, despite high PRMT5 expression being associated with breast cancer progression, aggressive disease, and poor prognosis (Chen et al., 2017; Powers et al., 2011; Yang et al., 2015). Using a systematic *in vitro* and *in vivo* approach to analyze the contribution of PRMT5 to BCSC function, we found that PRMT5 depletion in established estrogen receptor (ER)$^+$ xenografts not only reduced tumor growth but substantially reduced the proportion of BCSCs after serial transplantation. Significantly, treatment of BCSCs isolated from patient-derived tumors with a pre-clinical PRMT5 small-molecule inhibitor substantially reduced tumor-initiating potential. Our results thus demonstrate the importance of PRMT5-mediated arginine methylation for BCSC function and tumor initiation and imply that drug targeting of this pathway could have significant patient benefit by eradicating the cell population responsible for drug resistance and recurrence.

RESULTS

PRMT5, but Not PRMT1, Is Functionally Required for *In Vitro* BCSC Function in ER$^+$ Breast Cancers

PRMT1 and PRMT5 have been increasingly linked to stem cell function in normal and cancer cells (Banasavadi-Siddegowda et al., 2017; Blanc et al., 2017; Jin et al., 2016; Liu et al., 2015; Zhang et al., 2015) and breast cancer pathogenesis (Baldwin et al., 2012; Chen et al., 2017; Goulet et al., 2007; Powers et al., 2011; Yang et al., 2015). Whilst depletion of PRMT5 reduces the proliferation of bulk MCF7 breast cancer cells (Figure S1A; Scoumanne et al., 2009), no study has yet examined whether PRMT1 and PRMT5 also regulate the BCSC population. To address this, we exploited the fact that breast cancer cell lines possess a small population of cells that molecularly and functionally behave as cancer stem cells (Harrison et al., 2010; Ponti et al., 2005). Two approaches were used to isolate this population: flow cytometry gating on ESA$^+$CD24^{low}CD44$^+$ (Figure S1B) and the isolation of viable MCF7 cells after 16 hr suspension culture on poly-HEMA-coated plates to prevent cell attachment (Figure S1C). These anoikis-resistant (AR) cells are significantly enriched in stem cell markers compared with their monolayer counterpart and have tumor-initiating capacities (Harrison et al., 2010). Although PRMT1 levels remained the same, PRMT5 expression was significantly elevated within the AR or BCSC population (Figures 1A and 1B). To investigate the significance of this, we generated two PRMT1 and PRMT5-knockdown MCF7 cell lines each expressing distinct short hairpin RNA (shRNA) sequences (shPRMT1[1] and shPRMT1[2]; shPRMT5[1] and shPRMT5[2]; Figure 1C) and analyzed their ability to form mammospheres, a measure of the number and proliferative potential of tumor-initiating cells *in vitro* (Ponti et al., 2005). Depletion of either PRMT1 or PRMT5 reduced primary mammosphere formation, indicative of either reduced

Figure 1. PRMT5 Is Preferentially Expressed in BCSC-Enriched Cell Populations and Is Required for Self-Renewal of ER$^+$ Breast Cancer Cells
(A and B) Immunoblot of PRMT5 and PRMT1 levels in (A) anoikis-resistant and (B) ESA$^+$CD24^{low}CD44$^+$ BCSC-enriched populations of MCF7 cells, quantified below.
(C) Immunoblot of MCF7 cells expressing a non-targeting shRNA or two independent shRNAs targeting either PRMT1 or PRMT5 (shCTRL, shPRMT1[1], shPRMT1[2], shPRMT5[1] and shPRMT5[2]) (top). Mammosphere assay of cells after PRMT1 or PRMT5 depletion (bottom).
(D) Mammosphere assay of T47D ER$^+$ breast cancer cells expressing shCTRL, shPRMT5(1), or shPRMT5(2). PRMT5 depletion is shown (above). MFU, mammosphere-forming unit.
(E) ALDEFLUOR assay of T47D shCTRL, shPRMT5(1), or shPRMT5(2). Percentage ALDEFLUOR$^+$ cells is quantified (right).
(F) Immunoblot of PRMT5, MEP50, and pan-symmetric dimethylarginine (SDMA) in MCF7 cells engineered to express wild-type or enzymatically inactive PRMT5 (PRMT5-WT or PRMT5 G367A/R368A) and/or MEP50.
(G) Mammosphere assay of cells overexpressing PRMT5 and/or MEP50.
All data are mean ± SEM, n $\geq$ 3.

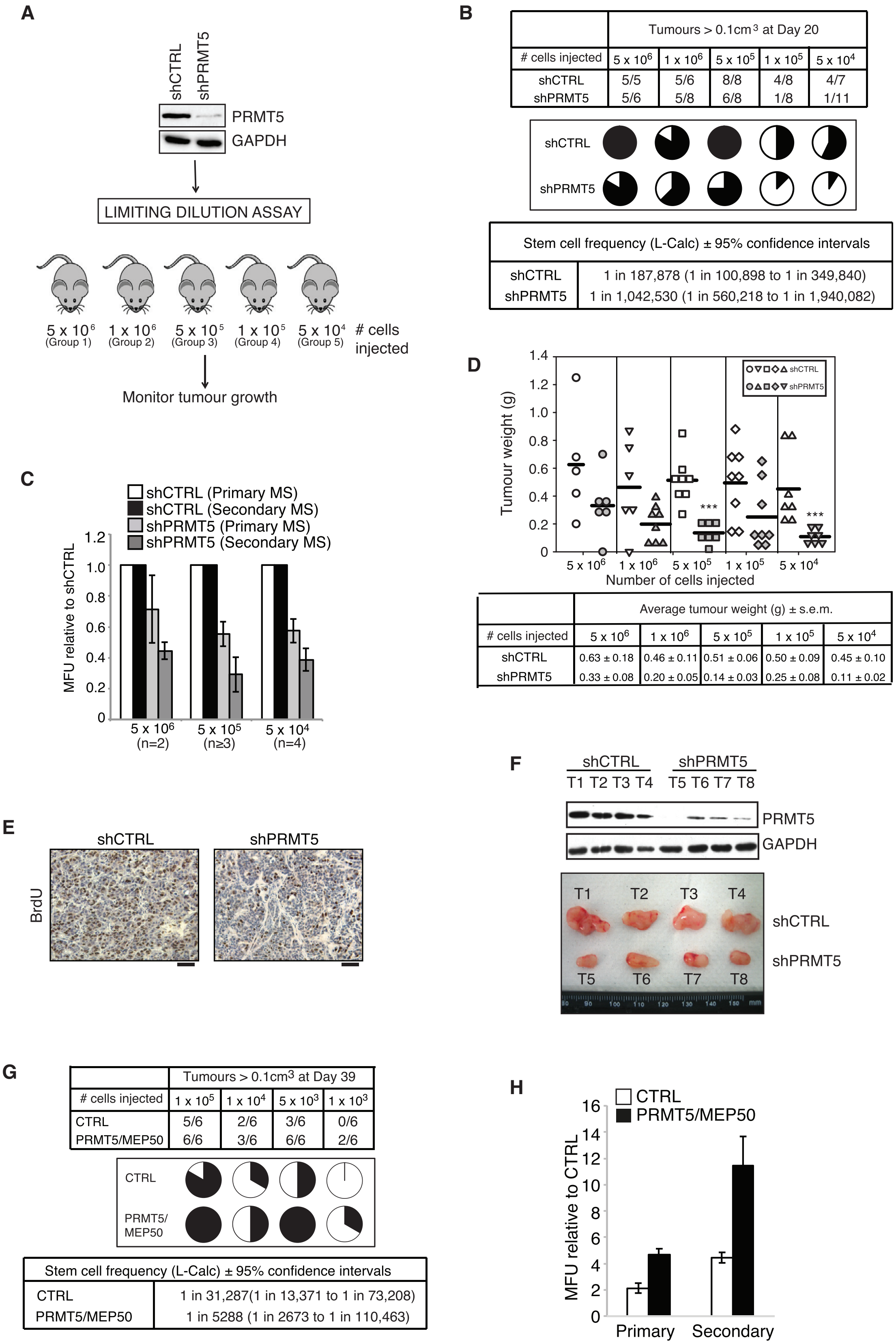

(legend on next page)

stem cell/progenitor proliferation or a reduction in the number of tumor-initiating cells, but only depletion of PRMT5 led to a further reduction in secondary mammospheres, suggesting self-renewal defects (Figure 1C). Thus, it appears that although both PRMT1 and PRMT5 are required for BCSC proliferation and production of progenitor cells, only PRMT5 is required for BCSC self-renewal. To validate these findings, we repeated these experiments in a second ER^+ breast cancer cell line, T47D (Figure 1D), and analyzed stem cell numbers by ALDEFLUOR staining, an alternative marker of BCSCs (Ginestier et al., 2007). As depletion of PRMT5 also reduced the proportion of $ALDEFLUOR^+$ T47D cells (Figure 1E), our data strongly imply that PRMT5 is required for maintaining BCSC function *in vitro*.

Amplification and overexpression of PRMT5 in breast cancer is associated with reduced patient survival rates (Figures S1D and S1E) (Cerami et al., 2012; Gao et al., 2013; Györffy et al., 2010). Since chromatin-modifying enzymes are known to regulate cellular events that can lead to the conversion of non-CSCs toward a CSC-like phenotype and the expansion of the BCSC pool (Chang et al., 2011; Tam and Weinberg, 2013), we next asked whether overexpression of active PRMT5 by co-expressing PRMT5 and its essential cofactor MEP50 was sufficient to promote a BCSC phenotype. As expected, co-expression of PRMT5 requires MEP50 for elevated symmetric dimethylation levels (Figures 1F, S1F, and S1G). Accordingly, overexpression of PRMT5 alone had no significant effect on primary mammosphere numbers, whereas co-expression of PRMT5 and MEP50 significantly increased both primary and secondary mammosphere numbers (Figure 1G). Interestingly, co-overexpression of catalytically inactive PRMT5 (PRMT5-G367A/R368A) (Pal et al., 2004) with MEP50 failed to stimulate BCSC activity (Figures 1F and 1G). These findings therefore imply that PRMT5 drives BCSC proliferation and self-renewal via the methylation of target substrates.

PRMT5 Is Required for *In Vivo* BCSC Maintenance

The gold-standard *in vivo* assay to determine tumor-initiating capacity of putative BCSCs is limiting dilution analysis. Here, groups of NSG (NOD/Scid/IL-2Rγnull) mice were subcutaneously injected with five different dilutions (5×10^6 to 5×10^4) of unsorted cells and monitored for tumor growth, the premise being that the more tumor-initiating cells present within the bulk tumor cell population, the fewer cells required to generate a tumor (Figure 2A). After 20 days, control animals from the 5×10^6 cell group reached license limit, and all mice were scored for the presence of tumors >0.1 cm^3 as evidence of tumor initiation. Depletion of PRMT5 clearly showed a reduction in the potential to form tumors at all cell numbers injected, becoming more evident with small cell numbers. For example, whereas 4 of 7 mice (57%) injected with 5×10^4 shCTRL cells developed tumors, only 1 of 11 mice (9.1%) injected with the same number of shPRMT5 cells did so. Strikingly, using L-Calc (Stem Cell Technologies) to calculate stem cell frequency, we show that depletion of PRMT5 reduced cancer stem cell numbers from 1:187,878 to 1:1,042,530, a 5.5-fold reduction (Figure 2B). In order to determine the effects of PRMT5 depletion on BCSC and tumor biology, mice in each group were aged and sacrificed when control animals reached license limit. Tumors were excised and analyzed for stem cell function by mammosphere assay. Importantly, the reduction in stem cell frequency was reflected *ex vivo*, as PRMT5-depleted cells from excised tumors displayed reduced proliferation and self-renewal characteristics (Figure 2C). Consistent with a reduction in stem cells, we observed that PRMT5-depleted tumors were less proliferative, as indicated by differences in final tumor weight, BrdU incorporation, and tumor growth rate (Figures 2D–2F and S2). Conversely, overexpression of active PRMT5 in limiting dilution xenografts increased stem cell numbers by 5.91-fold (Figures 2G, 2H, S3A, and S3B), which was reflected *ex vivo* by mammosphere assay (Figure 2H). Together, this *in vivo* analysis clearly shows that elevated levels of active PRMT5 are sufficient for BCSC activity.

Although our limiting dilution analysis demonstrates an important role for PRMT5 in regulating BCSCs, PRMT5 is also known to promote the proliferation of bulk breast cancer cells (Figure S1A; Scoumanne et al., 2009). In line with this, depletion of PRMT5 reduces both the *in vitro* and *in vivo* proliferation of isolated BCSCs and the differentiated population (Figures S3C–S3H). Importantly, and in agreement with our limiting dilution analysis using bulk cell population (Figures 2B and 2G), depletion of PRMT5 affected the tumor-initiating capacity of only $ESA^+CD24^{low}CD44^+$ BCSCs (Figure S3E), not the differentiated population (Figure S3G). Taken together, these results imply that PRMT5 has multiple roles in breast cancer growth,

Figure 2. PRMT5 Is Required for Maintenance of Stem Cells *In Vivo*

(A) Schematic of limiting dilution assay. MCF7 shCTRL or shPRMT5 cells were injected into NSG mice in a limiting dilution assay. PRMT5 levels of cells injected were assessed by immunoblotting prior to the experiment (top).

(B) Stem cell frequencies of MCF7 shCTRL and shPRMT5 cells were determined using L-Calc software. Upper table shows number of tumors with a positive response (response = tumor > 0.1 cm^3 at 20 days post-injection)/total number of tumors and is depicted in the pie chart diagram below. Lower table shows stem cell frequency ± 95% confidence intervals.

(C) Mammosphere (MS) assays of tumor-derived cells from the indicated cell number injected. For 5×10^6, error is mean ± range; for 5×10^5 and 5×10^4, error is mean ± SEM.

(D) Dot density plot of final tumor weight. The bar represents mean. Table below shows the average tumor weight ± SEM.

(E) Representative images of BrdU-stained tumor sections from shCTRL or shPRMT5-expressing cells. The scale bar represents 50 μm.

(F) Immunoblot of PRMT5 in tumors derived from 5×10^5 shCTRL or shPRMT5-expressing cells (above). Images of resected tumors (below).

(G) Stem cell frequencies of MCF7 CTRL or PRMT5/MEP50 cells were determined using L-Calc software. Upper table shows number of tumors with a positive response (response = tumor > 0.1 cm^3 at 39 days post-injection)/total number of tumors and is depicted below. Lower table shows stem cell frequency ± 95% confidence intervals.

(H) Mammosphere assay of cells isolated from CTRL and PRMT5/MEP50 tumors.

Unless otherwise stated, all data are mean ± SEM, n ≥ 3.

regulating both tumor-initiating and more differentiated cells, and that drug targeting of PRMT5 could potentially affect the survival of both cell populations.

To investigate the impact of PRMT5 depletion on tumor biology, we performed histological analysis of excised tumors in collaboration with a breast cancer pathologist and scored for pathological features currently used in patient diagnosis. We observed that PRMT5-depleted tumors displayed reduced cellularity, increased fibrosis, and tubule formation, consistent with a less aggressive phenotype (Figures 3A, 3B, and S4A). Control tumors often exhibited high cellularity and large areas of clear cell differentiation (Figure 3E), a phenotype of a particularly aggressive breast cancer subtype. Areas of necrosis, another feature of large, rapidly growing tumors, were also observed in shCTRL tumors (Figures 3C and 3D). These features were rarely exhibited in PRMT5-depleted tumors, correlating with their small size. Interestingly, depletion of PRMT5 in AR or $ESA^{+}CD24^{low}CD44^{+}$ populations did not alter cell cycle or promote apoptosis (Figures 3F and 3G). Hence, given that PRMT5-depleted tumors appear more differentiated with a less aggressive pathology, our findings are consistent with PRMT5 suppressing differentiation and thus facilitating the maintenance of BCSC identity.

PRMT5 Is Required for BCSC Function in Established Tumors

Recently, PRMT5 has become an attractive therapeutic target for the treatment of leukemia and lymphoma, with pre-clinical PRMT5-specific inhibitors displaying impressive *in vivo* efficacy in murine models of mantle cell lymphoma and chronic myelogenous leukemia (CML) (Chan-Penebre et al., 2015; Jin et al., 2016). However, these studies did not address the effect of suppressing or inhibiting PRMT5 on tumor growth in an organism that had already presented with disease, a critical question if PRMT5 inhibitors are to be a viable therapeutic option. We therefore engineered MCF7 cells to express a doxycycline (dox)-regulated PRMT5 silencing construct (Tet-ON-shPRMT5) in combination with constitutive expression of the luciferase gene enabling *in vivo* imaging of tumor growth (Figures 4A and 4B). After subcutaneous injection, tumors were allowed to grow to a palpable size (Figure S4B) and *in vivo* PRMT5 depletion induced by feeding mice dox-supplemented chow (Figures 4A and 4B). Tumors derived from Luc-Tet-ON-shCTRL cells continued to grow for the duration of the experiment. In contrast, *in vivo* depletion of PRMT5 substantially slowed tumor growth and in some cases caused regression (Figures 4C and 4D). Accordingly, Luc-Tet-ON-shPRMT5-derived tumors were considerably smaller than control tumors (Figures 4E and 4F).

Although these results suggest that PRMT5 is required to sustain the growth of an established carcinoma, we wanted to determine if this was through the maintenance of the BCSC population. We therefore serially transplanted cells derived from Luc-Tet-ON-shCTRL and shPRMT5 tumors and conducted limiting dilution analysis. Excised tumors were dissociated into single cells, and five groups of NSG mice were subcutaneously injected with 1×10^5 to 1×10^3 tumor-derived cells (Figure 5A). PRMT5 silencing in the transplanted cells was validated by immunoblotting, and tumors were allowed to develop for 40 days. As expected, mice injected with larger numbers of cells were more likely to develop tumors than those injected with small cell numbers (Figure 5C); however, irrespective of cell number, PRMT5-depleted serially transplanted tumors grew at a much slower rate (Figures 5B and 5D). Furthermore, examination of the 1×10^3 cell group clearly demonstrated that whereas serially transplanted Luc-Tet-ON-shCTRL-derived tumors steadily outgrew, Luc-Tet-ON-shPRMT5-derived tumors cells failed to generate any growths (Figure S4C). These findings strongly indicate that *in vivo* depletion of PRMT5 alters the functional capacity of tumor-initiating cells. Indeed, by scoring the number of mice that presented with tumors $>0.1\ cm^3$ at time of collection (all mice irrespective of group were collected at the same time), Luc-Tet-ON-shPRMT5-derived, serially transplanted tumors displayed a 12-fold reduction in stem cell frequency, from 1:3,555 to 1:43,135, after *in vivo* PRMT5 depletion (Figure 5C). Consistent with a reduced tumorigenic capacity, PRMT5-depleted xenografts were of a lower cellularity, displayed frequent tubule formation, and were sometimes highly fibrotic, resulting in a greater proportion of tumors with a lower histological grade (Figures 5E, S4D, and S4E). In support, mammosphere analysis of resected and dissociated tumors demonstrated that PRMT5 depletion reduced mammosphere-forming capacity *ex vivo* (Figure 5F). Taken together, our data are highly suggestive that depletion of PRMT5 in established carcinomas reduces tumor propagation by restricting the number of BCSCs.

PRMT5 Epigenetically Regulates *FOXP1* Expression

Because PRMT5 is an established regulator of gene expression (Migliori et al., 2012; Zhao et al., 2009), we hypothesized that one mechanism by which PRMT5 could be regulating BCSC function was through transcriptional control. To investigate this, we isolated three replicates of the $ESA^{+}CD24^{low}CD44^{+}$ populations from MCF7-shCTRL and shPRMT5 cells and conducted RNA sequencing (RNA-seq) to identify differences in gene expression. Depletion of PRMT5 resulted in 214 significantly differentially regulated genes, of which 136 were upregulated and 78 downregulated (Figures S5A–S5C). KEGG pathway analysis of enriched genes suppressed by PRMT5 (i.e., those that were upregulated after shRNA depletion) identified cancer pathways and signaling pathways (including cGMP-PGK, PI3K-AKT, ErbB, and FoxO pathways) (Figure S5D), while metabolic genes are predominantly induced by PRMT5 (*ALDOC*, *HK2*, *TSTA3*, and *ALDH3A1*; Figure S5E). Changes in expression of classic stem cell genes such as *OCT4* or *NANOG* were not detected by RNA-seq. However, closer examination by qPCR analysis of AR cells reconstituted with wild-type PRMT5 did imply that they were PRMT5 regulated (Figure S6A). Interestingly, Wnt/β-catenin genes, reported as PRMT5 targets in leukemic stem cells (Jin et al., 2016; Tee et al., 2010), could not be validated by qPCR (*Wnt5A*, *PRICKLE2*), implying that the mechanisms by which PRMT5 regulates stem cells in breast cancer and leukemia are different.

Surprisingly, none of our top differentially expressed validated genes appeared to have a close correlation with breast cancer pathogenesis (Figures S5B and S5C), and although *CDKN1A* was validated as a PRMT5-repressed gene (Figure S6B), the

OPEN ACCESS
CellPress

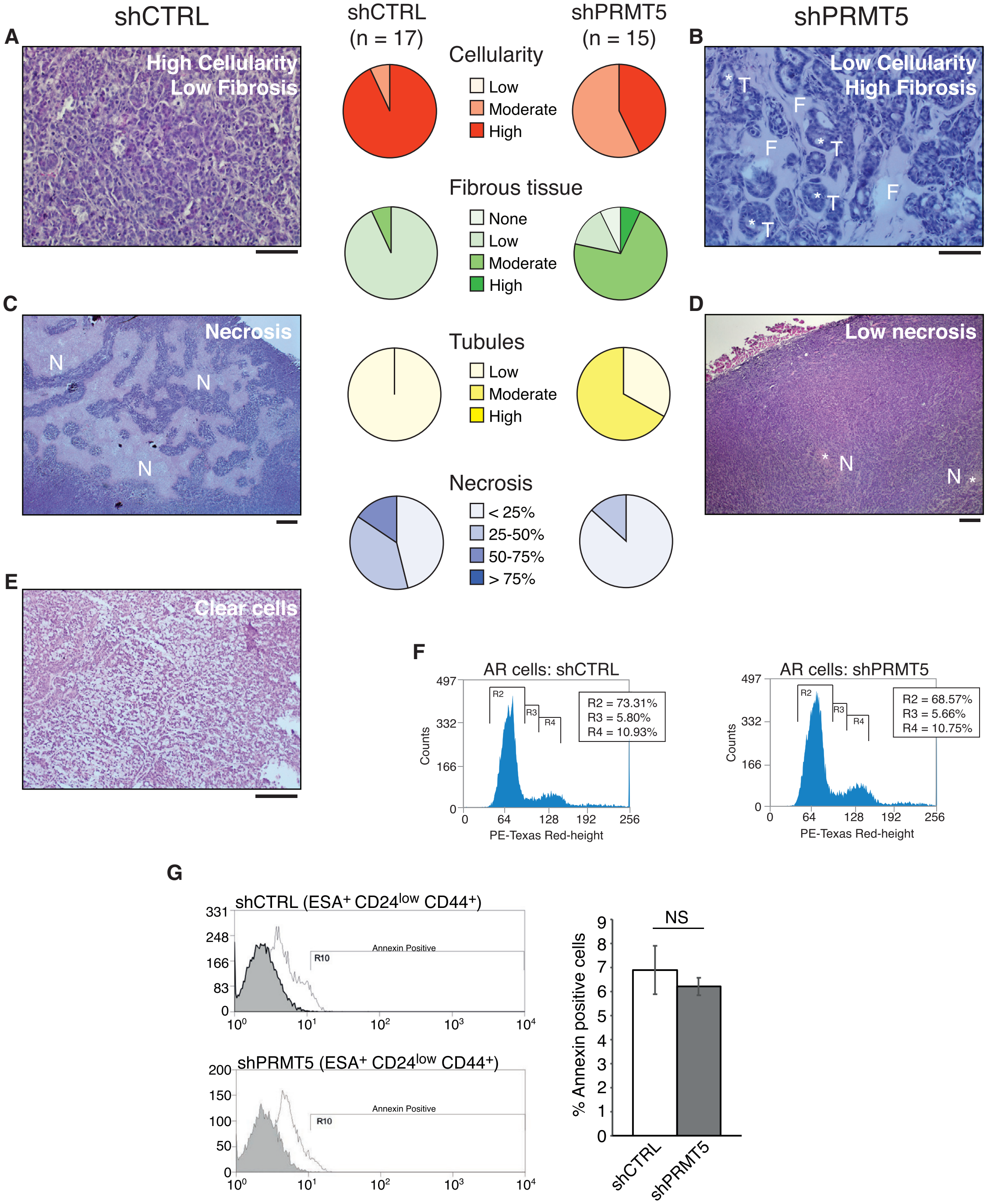

Figure 3. Depletion of PRMT5 Reduces Tumor Aggressiveness but Does Not Affect Cell Cycle or Apoptosis

(A–E) Tumors were assessed for pathological features and are depicted in pie charts as indicated. Representative images of (A and B) cellularity, fibrosis, tubule formation; (C and D) necrosis; and (E) clear cells. F, fibrous tissue; N, necrotic areas; T, tubule. The scale bar represents 100 μm.

(F) Cell cycle profiles of shCTRL or shPRMT5 AR cells.

(G) Annexin-FITC^{+} cells in ESA^{+}CD24^{low}CD44^{+} cells, quantified (right). NS, not significant.

All data are mean ± SEM, n ≥ 3.

functional significance of this is unclear given that the cell cycle profiles of shCTRL or PRMT5-depleted AR cells were indistinguishable (Figure 3F). We thus decided to focus our investigations on FOXP1, a winged helix/forkhead transcription factor that has been associated with normal and cancer stem cell function (Choi et al., 2016; Gabut et al., 2011; Naudin et al.,

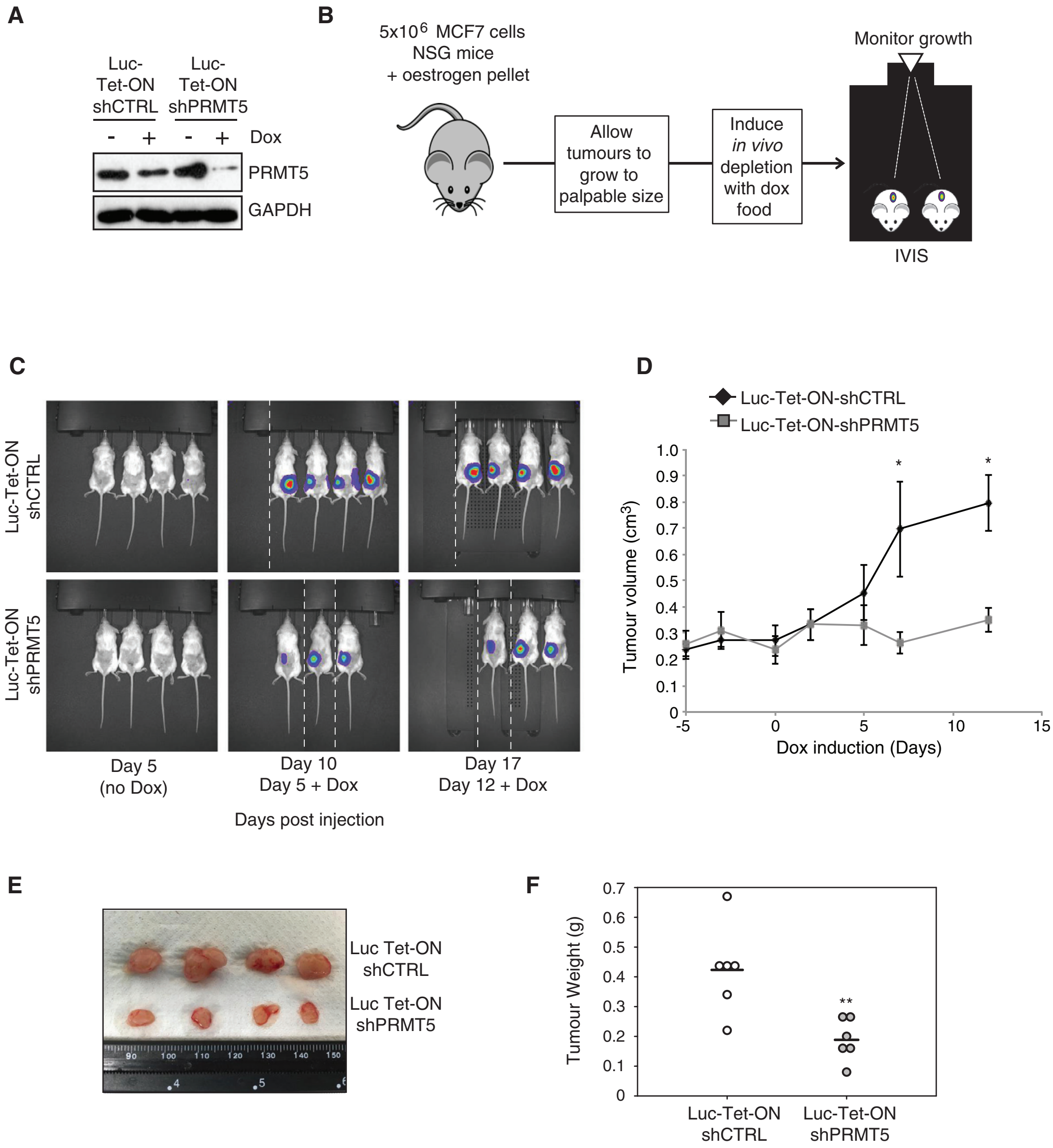

Figure 4. *In Vivo* Depletion of PRMT5 Reduces Tumor Growth

(A) Immunoblot of PRMT5 in MCF7 Luc-Tet-ON-shCTRL and shPRMT5 cells ± 1 μg/mL doxycycline (dox) for 5 days.

(B) Schematic of study. Mice were injected with MCF7 cells expressing dox-inducible Luc-Tet-ON-shCTRL or shPRMT5. Tumors were allowed to develop, and when palpable, mice were fed dox-supplemented chow to induce shRNA expression. Tumor growth was monitored by IVIS and caliper measurements.

(C) IVIS images of mice injected with Luc-Tet-ON shCTRL or shPRMT5 cells at times shown. Images were taken using the same exposure settings. Exposure time = 1 s. (In some cases, mice were reimaged after unsuccessful luciferin injection. Images have been cropped accordingly [dotted white lines].)

(D) Growth of tumors before and after dox-supplemented diet.

(E) Image of resected tumors from Luc-Tet-ON shCTRL or shPRMT5 cells.

(F) Dot density plot of final tumor weight. The bar represents the mean.

All data are mean ± SEM, n ≥ 3.

2017). Intriguingly, FOXP1 is generally considered a tumor suppressor in the breast because high levels correlate with better prognosis, despite the finding that it promotes proliferation and migration of breast cancer cell lines (Oskay Halacli, 2017; Shigekawa et al., 2011; Xiao et al., 2016). Because of this apparent contradiction and link with stem cell biology (Choi et al., 2016; Gabut et al., 2011), we decided to investigate the relationship between PRMT5, FOXP1, and BCSC function in more detail.

Similar to PRMT5, FOXP1 levels were significantly elevated in AR cells (Figure 6A). Importantly, expression was PRMT5 dependent, as depletion of PRMT5 decreased both protein (full-length

OPEN
ACCESS
CellPress

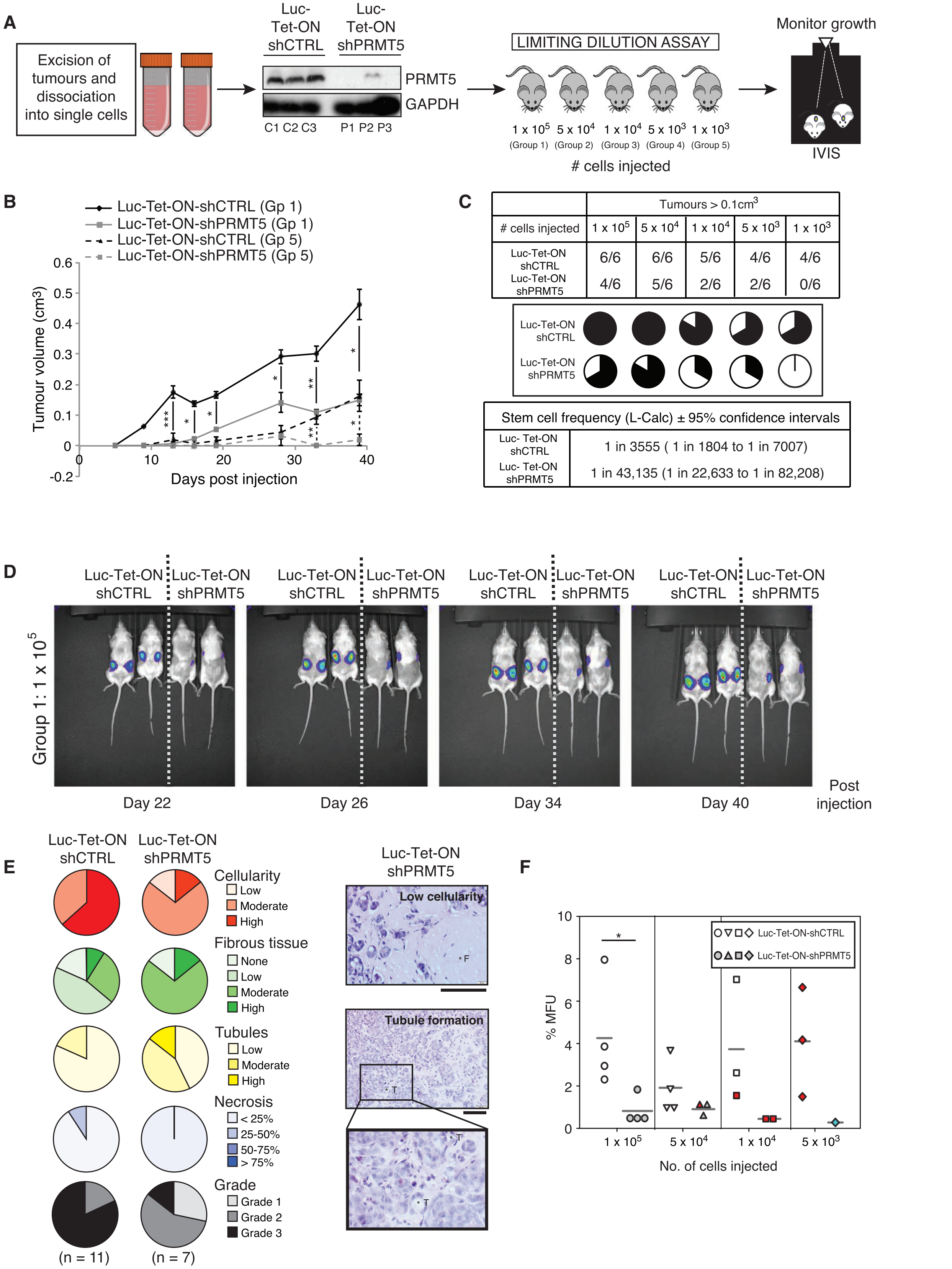

(legend on next page)

75 kDa FOXP1 isoform) and mRNA transcripts in AR and ESA$^+$ CD24^{low}CD44$^+$ cells (Figures 6B–6D). Moreover, *FOXP1* expression was dependent on the catalytic activity of PRMT5 as reconstitution of PRMT5-depleted cells with ectopic wild-type but not catalytically inactive PRMT5 increased *FOXP1* transcripts (Figure 6C). We next wanted to determine if PRMT5 was directly regulating *FOXP1* expression. Using total cell population, we used chromatin immunoprecipitation (ChIP) coupled to promoter tiling to demonstrate an enrichment of PRMT5 at the *FOXP1* promoter. In comparison, PRMT5 was not recruited to the *MYOD1* promoter, a muscle-specific gene regulated by PRMT5 but not expressed in MCF7 cells (Dacwag et al., 2007) (Figures 6E and S7A and S7B). Importantly, PRMT5 depletion significantly reduced PRMT5 promoter occupancy (Figures 6E and S7B), confirming that PRMT5 is truly recruited to the *FOXP1* promoter and validating specificity of our PRMT5 ChIP antibody. One mechanism by which PRMT5 promotes gene expression is through the methylation of H3R2 (H3R2me2s), which is recognized by WDR5, enabling the recruitment of the SET1/MLL complex and histone H3K4 trimethylation (H3K4me3) (Migliori et al., 2012). Consistent with this chain of events, H3R2me2s, SET1, and H3K4me3 were all enriched at the *FOXP1* promoter in a PRMT5-dependent manner (Figures 6F–6H and S7C–S7E). Crucially, this observation was also true in the tumor-initiating population, as treatment of AR cells with the PRMT5 inhibitor GSK591 (Duncan et al., 2015) substantially reduced *FOXP1* promoter H3K4me3 levels (Figure 6I), and treatment of primary mammospheres with the WDR5 antagonist OICR-9429 (Grebien et al., 2015) reduced BCSC proliferation in a dose-dependent manner (Figure S7G). Even though global levels of H3R2me2s were not altered by PRMT5 depletion (Figure S7H), our findings are highly supportive that PRMT5 is directly recruited to the *FOXP1* promoter, facilitating H3K4me3 and gene expression via H3R2me2s.

Given that FOXP1 has been proposed to function as a tumor suppressor in the breast, we wanted to determine the effect of FOXP1 on MCF7 growth. In support of a pro-tumorigenic role, FOXP1 depletion in MCF7 cells results in growth suppression, whereas overexpression enhances growth (Figures 6J and 6K). More significantly, depletion of FOXP1 in xenografts significantly reduced tumor growth (Figures 6L and S7F). Indeed, transcript levels of *FOXP1* are elevated >11-fold in MCF7 cells compared with immortalized but non-transformed mammary epithelial MCF10A cells (Figure 6D). Next, we wanted to determine if PRMT5-mediated upregulation of FOXP1 is required for BCSC function. Interestingly, FOXP1 depletion in MCF7 cells expressing endogenous levels of PRMT5/MEP50 reduced primary mammosphere numbers but did not significantly affect secondary mammospheres (Figures 6M and 7B), implying that under these conditions, FOXP1 regulates BCSC numbers and proliferation rather than self-renewal. In contrast, depletion of FOXP1 on the background of ectopically expressed PRMT5 and MEP50 completely abrogated the effects of PRMT5 and MEP50 overexpression on both BCSC numbers and proliferation and self-renewal (Figure 6M), implying that the effects of hyperactive PRMT5 on BCSC proliferation and self-renewal are mediated through FOXP1. These findings therefore suggest that a threshold level of FOXP1 expression, presumably via PRMT5-mediated epigenetic regulation, is required for self-renewal or that high levels of an unknown FOXP1 cofactor that drives self-renewal gene expression is present only under certain PRMT5 conditions.

Pharmacological Inhibition of PRMT5 Reduces BCSC Numbers *In Vitro*

Because we demonstrated that the catalytic activity of PRMT5 was required for driving BCSC function and FOXP1 expression (Figures 1G and 6C), we wanted to determine whether a small-molecule inhibitor targeting PRMT5 could affect BCSC function. We therefore treated MCF7 cells with the validated PRMT5 inhibitor GSK3203591 (EPZ015866; herein referred to as GSK591) (Figure 7A; Duncan et al., 2015). Similar to PRMT5 depletion, inhibition of PRMT5 significantly suppressed MCF7-derived BCSC proliferation and self-renewal and the number of ALDEFLUOR$^+$ T47D cells (Figures 7B and S7I), while overexpression of FOXP1 was able to rescue the BCSC proliferative defect induced by GSK591 (Figure 7C). Our data thus imply that drug targeting PRMT5 reduces the activity of BCSCs, but given that depletion of FOXP1 and GSK591 was not epistatic in reducing primary and secondary mammospheres (Figure 7B), FOXP1 is important but not sufficient for all PRMT5-dependent events in BCSCs.

PRMT5 has been reported as a critical component of normal stem cell function (Chittka et al., 2012; Liu et al., 2015), hence one potential limitation of PRMT5-directed therapies for breast cancer is the suppression of normal mammary stem cell function. Interestingly, although knockdown and inhibition of PRMT5 reduces the number of primary mammospheres of MCF10A cells, self-renewal was unaffected (Figures 7B, S7I, and S7J). In support, stem cell numbers,

Figure 5. *In Vivo* Depletion of PRMT5 Reduces Stem Cell Frequency

(A) Schematic of study. Following *in vivo* depletion of PRMT5, MCF7 Luc-Tet-ON-shCTRL and shPRMT5 tumors were harvested. Immunoblot of PRMT5 in resected tumors (C1-3 and P1-3 denote Luc-Tet-ON-shCTRL and PRMT5 tumors, respectively) that were dissociated into single cells and injected into NSG mice in a limiting dilution assay. Tumor growth was monitored by IVIS and caliper measurements.

(B) Tumor growth in mice injected with 1×10^5 cells (Gp1; solid line) or 5×10^3 cells (Gp5; dashed line).

(C) Stem cell frequencies of Luc-Tet-ON shCTRL and shPRMT5 cells were determined using L-Calc software. Upper table shows number of tumors with a positive response (response = tumor > 0.1 cm^3)/total number of tumors and is depicted below. Lower table shows stem cell frequency ± 95% confidence intervals.

(D) IVIS images of mice injected with 1×10^5 tumor-derived Luc-Tet-ON shCTRL or shPRMT5 cells at the specified time points.

(E) Tumors were assessed for pathological features and depicted in pie charts as indicated. Representative images (right) of pathological features. F, fibrous tissue; T, tubule. The scale bar represents 100 μm.

(F) Dot density plot of mammosphere formation of tumor-derived cells. The bar represents the mean. Red, pool of two tumors; blue, pool of three tumors.

All data are mean ± SEM, n ≥ 3.

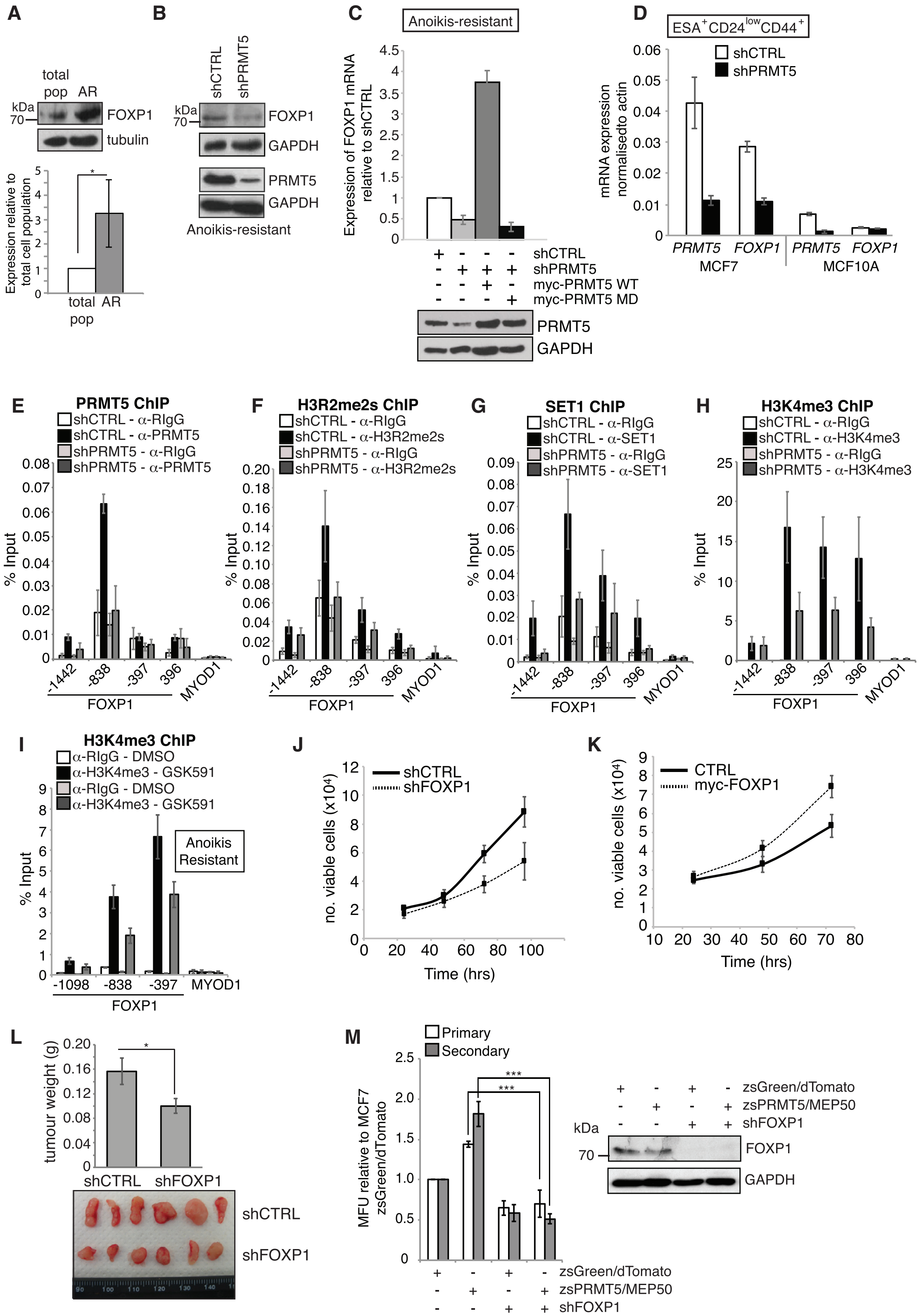

(legend on next page)

as determined by ALDEFLUOR staining was unaffected by GSK591 (Figure S7I). Moreover, this lack of effect of PRMT5 deletion or inhibition on MCF10A self-renewal was not due to receptor status, as treatment of the triple-negative breast cancer cells SUM159 with GSK591 phenocopies that of MCF7 cells, reducing both BCSC proliferation and self-renewal (Figure 7B). Hence, it appears that PRMT5 activity specifically affects self-renewal of cancer rather than normal mammary stem cells.

We next wanted to evaluate the ability of PRMT5 depletion to synergize with conventional chemotherapy for ER^+ tumors. Although PRMT5 depletion did not alter the ability of 4-hydroxytamoxifen (4-OHT), the active metabolite of tamoxifen, to suppress primary mammosphere numbers (Figure 7D), secondary mammosphere formation was markedly reduced (Figure 7E). Thus, PRMT5 inhibitors in combination with 4-OHT appear to synergistically suppress BCSC self-renewal.

To correlate our cell culture findings with therapeutic potential, we isolated cancer cells from freshly resected ER^+ patient-derived tumors from a cohort that had not undergone neo-adjuvant chemotherapy and conducted mammosphere analysis in the presence of GSK591. Continuous treatment of patient-derived BCSCs with GSK591 markedly suppressed mammosphere formation, indicative that inhibition of PRMT5 is effectively depleting primary-derived BCSCs (Figure 7F). Taken together, our results strongly imply that therapeutic targeting of PRMT5 could be an effective way of eradicating the cancer stem cell compartment.

DISCUSSION

PRMT5 has been increasingly associated with maintaining normal cell and leukemic cell “stemness” (Jin et al., 2016; Liu et al., 2015; Tee et al., 2010; Zhang et al., 2015), but very little is known about the role of PRMT5 in cancer stem cells driving carcinoma formation. In this study, we confirm that PRMT5 regulates the proliferation of bulk breast cancer cells and, more importantly, define a critical role for PRMT5 in the maintenance and propagation of BCSCs *in vitro* and *in vivo* through the epigenetic regulation of FOXP1. These findings are of high clinical relevance because small-molecule inhibitors of PRMT5 are in pre-clinical development, exhibiting good *in vivo* efficacy against lymphomas and leukemia (Chan-Penebre et al., 2015; Jin et al., 2016). Hence, drug targeting PRMT5 in the breast could have a dual effect of not only targeting more differentiated cancer cells but also effectively eliminating the tumor-initiating/BCSC population.

One key observation in this study is that PRMT5 depletion in established tumors, thereby mimicking a patient presenting with disease, substantially decreases BCSC frequency, implying that PRMT5 inhibitors could potentially eradicate this population to prevent relapse. Indeed, our data showing that treatment of patient-derived BCSCs with the tool PRMT5 inhibitor GSK591 decreases BCSC frequency and proliferation represent, to our knowledge, the first account of a PRMT5 inhibitor influencing cancer stem cells derived from solid cancers. One concern with epigenetic-based therapies is that many chromatin remodeling enzymes are essential for normal homeostatic function. Indeed, PRMT5 is an essential gene with deletion causing early embryonic lethality in mice (Tee et al., 2010). Surprisingly, we found that although PRMT5 depletion or inhibition did affect the proliferation of the non-transformed mammary epithelial cell line, MCF10A, self-renewal was unaffected, implying that either PRMT5 is not required for self-renewal of normal mammary stem cells or that residual methyltransferase activity after knockdown or inhibition is sufficient to maintain normal function. Indeed, PRMT5 expression levels are elevated in BCSCs compared with normal mammary stem cells (Figure 6D), implying that BCSCs are potentially more dependent on PRMT5 activity. Similarly, leukemic CML $CD34^+$ cells have elevated PRMT5 expression compared with normal bone marrow $CD34^+$ cells, and PRMT5 inhibition only affects the self-renewal of leukemic $CD34^+$ stem cells (Jin et al., 2016). Together, our results in conjunction with others imply that elevated PRMT5 expression within cancer stem cells offers a therapeutic window for drug treatment.

We identify that one mechanism by which PRMT5 regulates BCSCs is via the epigenetic regulation of FOXP1. PRMT5 is recruited to the FOXP1 promoter, leading to H3R2me2s, SET1 binding, and H3K4me3. Interestingly, although PRMT5-dependent H3K4me3 is a common mechanism for gene regulation in both leukemic and BCSCs, the genes that are targeted appear to be specific to the origin of the cancer stem cell, implying that the cellular mechanisms by which stemness is maintained by PRMT5 are distinct. Our results clearly show that FOXP1 is an important target of PRMT5, as overexpression can rescue the BCSC proliferation defect induced by GSK591, whereas knockdown decreases BCSC proliferation and self-renewal induced by hyperactive PRMT5. However, depletion of FOXP1 and PRMT5 inhibition are not epistatic for primary

Figure 6. PRMT5 Epigenetically Regulates FOXP1 Expression

(A) FOXP1 (75 kDa isoform) and PRMT5 protein levels were assessed in the AR population in MCF7 cells and quantified below.

(B) Immunoblot of FOXP1 levels in AR cells after PRMT5 depletion.

(C) *FOXP1* expression was assessed by qPCR in AR cells isolated from MCF7 shCTRL, shPRMT5, and shPRMT5 + PRMT5 (WT)/PRMT5 G367A/R368A (MD) cells. Immunoblot of PRMT5 (below).

(D) *PRMT5* and *FOXP1* transcript levels were assessed in MCF7 or MCF10A cells after PRMT5 depletion.

(E–H) Enrichment of (E) PRMT5, (F) H3R2me2s, (G) SET1, and (H) H3K4me3 or rabbit IgG at the *FOXP1* promoter was assessed by ChIP-qPCR.

(I) H3K4me3 enrichment at the *FOXP1* promoter in AR cells ± GSK591.

(J and K) Growth curve of MCF7 cells after (J) FOXP1 depletion or (K) FOXP1 overexpression.

(L) Average weight of shCTRL or shFOXP1 tumors. Images of resected tumors (below).

(M) Mammosphere assay of FOXP1-depleted cells overexpressing PRMT5 and MEP50. Immunoblot of FOXP1 (right).

All data are mean ± SEM, $n \geq 3$.

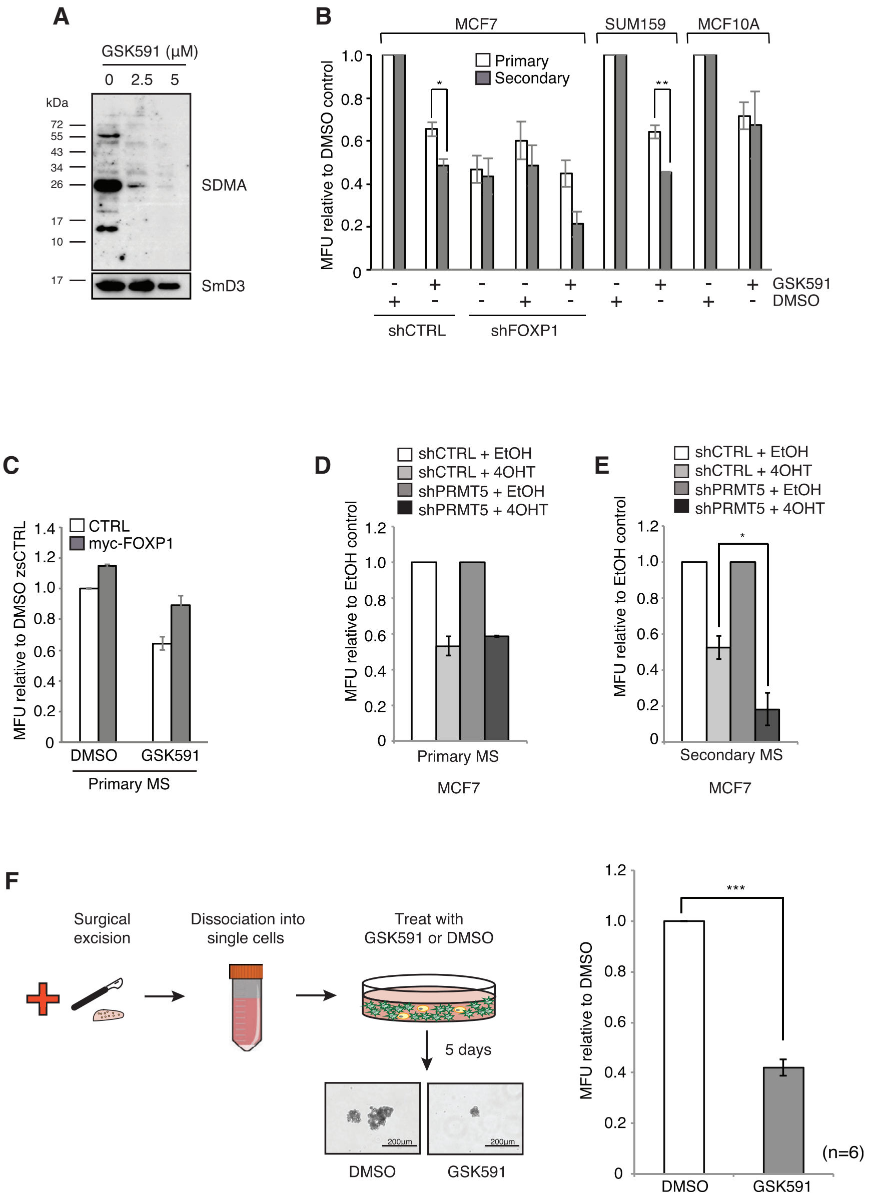

Figure 7. Inhibition of PRMT5 Reduces Self-Renewal in Cancer Cells and Sensitizes Mammospheres to 4-OHT Treatment

(A) Inhibition of PRMT5 activity using GSK591 was shown by immunoblotting for pan-symmetric dimethylarginine (SDMA). SmD3, a known target of PRMT5 was used as a loading control.

(B) Mammosphere assay of MCF7 shCTRL or shFOXP1, SUM159 (triple negative), and MCF10A cells treated with 5 μM GSK591 or DMSO (vehicle control).

(C) Mammosphere assay of MCF7 cells overexpressing FOXP1 ± 5μM GSK591.

(D) Primary mammosphere assay of MCF7 shCTRL or shPRMT5 cells ± 2.5 μM 4-OHT.

(E) Primary mammospheres from (D) were dissociated and replated. Secondary mammospheres were scored.

(F) Schematic of experiment. Patient-derived tumors were dissociated into single cells. Mammosphere assay of tumor-derived cells ± 5μM GSK591 (right). Representative images of mammospheres are shown.

All data are mean ± SEM, n ≥ 3.

and secondary mammosphere formation (Figure 7B), indicating that although FOXP1 is important, it is not the sole PRMT5 effector. Indeed, given that more than 200 genes are significantly deregulated after PRMT5 depletion, it would be interesting to understand which of these genes are direct epigenetic targets of PRMT5 and which are regulated downstream of FOXP1.

To date, immunohistochemical analysis has suggested that FOXP1 functions as a breast cancer tumor suppressor gene, as low levels correlate with poor prognosis (Xiao et al., 2016). In contrast, our findings clearly show that FOXP1 is pro-oncogenic, promoting breast cancer cell proliferation and *in vivo* tumor growth. One reason for the apparent discrepancy is that interpretation of FOXP1 immunohistochemistry is challenging. At least seven isoforms of FOXP1 are expressed, which are all detected by the JC12 antibody (Brown et al., 2008). Given that some isoforms are known to be more oncogenic (Choi et al., 2016; Gabut et al., 2011) or promote stem cell function (Gabut et al., 2011), our results highlight the necessity of understanding the pathological role of a protein before the significance of clinical correlations is made. Indeed, our observations that FOXP1 contributes to BCSC function build upon a growing connection between FOXP1 and normal and cancer stem cell biology (Gabut et al., 2011; Choi et al., 2016; Naudin et al., 2017). Interestingly, in ESCs, a switch in FOXP1 isoform usage is responsible for pluripotent gene induction while concurrently repressing differentiation genes (Gabut et al., 2011). This is intriguing because PRMT5 is a major regulator of alternative splicing events (Bezzi et al., 2013). Analysis of *MDM4* splicing failed to detect expression of the splice isoform *MDM4S* (Figure S6C), and our RNA-seq analysis was not at sufficient read depth to detect alterations in splicing patterns, but it would be important to fully explore whether in addition to directly regulating *FOXP1* transcription, PRMT5 might also direct *FOXP1* alternative splicing and isoform use.

In summary, our findings reveal important insights that link arginine methylation to the maintenance of the tumor-initiating BCSC population. Given the development of pre-clinical small molecules targeting PRMT5, combination treatment of PRMT5 inhibitors, along with conventional chemotherapy enabling tumor de-bulking, could have a significant impact on long-term outcomes for breast cancer patients.

EXPERIMENTAL PROCEDURES

Isolation of Stem Cell-Enriched Populations

BCSCs were isolated by either flow cytometry sorting of $ESA^{+}CD24^{low}CD44^{+}$ MCF7 cells or by isolation of AR cells using the Miltenyi Dead Cell Removal kit. For further details, see Supplemental Experimental Procedures.

Generation of Stable Cell Lines

Cell lines stably expressing shRNA sequences were generated by transducing with lentiviral supernatant containing 8 μg/mL polybrene (Sigma-Aldrich) by centrifugation for 90 min at 4,000 rpm. See Supplemental Experimental Procedures for more details and shRNA sequences.

Mammosphere Assay

MCF7, T47D, and MCF10A cells were plated onto pHEMA (Sigma-Aldrich)-coated six-well plates and cultured at 37°C for 5 days. Mammospheres >50 μm were scored using a graticle. For serial replating, mammospheres were disaggregated and cultured for a further 5 days prior to scoring. For drug treatment, cells were incubated with 5 μM GSK591 or 2.5 μM 4-OHT (Sigma-Aldrich) or vehicle controls. For further details, see Supplemental Experimental Procedures.

Xenograft Studies and *In Vivo* Imaging

Animal experiments were conducted in accordance with United Kingdom Home Office regulations. For the limiting dilution assay, 5- to 7-week-old female NSG mice were injected with the appropriate number of cells and a slow release estrogen pellet (NE-121; Innovative Research of America) was subcutaneously implanted at the base of the tail. Two hours prior to harvest at the experimental endpoint, mice were injected with 100 mg/kg BrdU (Sigma-Aldrich). For *in vivo* depletion of PRMT5, female NSG mice were injected with 5×10^6 cells and once tumors were palpable, maintained on a diet of dox chow (T-5BQ8-1816629-203; TestDiet). For imaging, mice were injected intraperitoneally (i.p.) with 150 mg/kg of luciferin (Promega), and images were captured on an IVIS Spectrum. See Supplemental Experimental Procedures for more detail.

RNA Sequencing

Three independent replicates of $ESA^{+}CD24^{low}CD44^{+}$ cells were isolated by flow cytometry from MCF7 shCTRL or shPRMT5 cells. Total RNA was extracted and rRNA was depleted prior to sequencing on the Illumina Hiseq. Sequenced reads were mapped to build hg19 of the human genome from the University of California, Santa Cruz (UCSC) genome database. Differential gene expression was determined using Cuffdiff 2.2.1 with a threshold of false discovery rate (FDR) <0.05 and fold change >1.5. Enriched Gene Ontology terms and KEGG pathways were identified using DAVID (https://david.ncifcrf.gov) (Huang et al., 2009). For more detail, see Supplemental Experimental Procedures.

ChIP

Chromatin extraction and ChIP were performed as described previously (Clarke et al., 2017). Briefly, cells were fixed, lysed, and sonicated to produce DNA fragments of between 300 and 500 bp. Chromatin was precleared and ChIP was performed using chromatin equivalent to 5×10^6 cells. Chromatin was incubated overnight at 4°C with antibody followed by 3 hr incubation with protein G beads. Immunoprecipitated DNA was reverse crosslinked and treated with proteinase-K. DNA was isolated by phenol/chloroform extraction and ethanol precipitation. For ChIP of AR cells, cells were treated for 5 days with 5 μM GSK591 prior to replating overnight in suspension. AR cells were isolated using the Miltenyi Dead Cell Removal kit. Following isolation, cells were processed as above. For more detail, see Supplemental Experimental Procedures.

Statistical Analysis

Unless otherwise stated, error bars represent mean ± SEM, $n \geq 3$ animals or experimental repeats. All statistical analysis was carried out using Student's t test (*$p < 0.05$, **$p < 0.001$, *** $p < 0.005$).

DATA AND SOFTWARE AVAILABILITY

Original scans of all immunoblots presented in this manuscript can be found at https://data.mendeley.com/datasets/h4y9yxzk3k/draft?a=d426c8ce-edd6-4a3a-9d7f-1e0aac13565a.

The accession number for the RNA-seq data reported in this paper is GEO: GSE107762.

SUPPLEMENTAL INFORMATION

Supplemental Information includes Supplemental Experimental Procedures and seven figures and can be found with this article online at https://doi.org/10.1016/j.celrep.2017.11.096.

ACKNOWLEDGMENTS

We thank members of the Birmingham Centre for Genome Biology (BCGB), particularly Jo Morris, for helpful discussion; Alison Banham (University of Oxford) for her kind gift of the FOXP1 antibody and discussions; Keith Brennan (University of Manchester) for lentiviral plasmids; Matthew MacKenzie (University of Birmingham) for flow cytometry; Johanna Dieguez Navas (University of Birmingham) at the Human Biomaterials Resource Centre (HBRC) for tissue processing; and all staff at the Biomedical Services Unit (BMSU). This work was supported by the University of Birmingham Fellowship awarded to C.C.D., a Breast Cancer Now grant to C.C.D. (2014NovPR352), and a Medical Research Council (MRC) grant to C.C.D. (MR/M009912/1). We are very grateful for financial support from Neil and Hillary Murphy via the Circles of Influence scheme (University of Birmingham). We dedicate this paper to the late Professor Adele Francis, an exceptional breast cancer surgeon, whose endless enthusiasm for breast cancer research and the bridging of clinical and scientific research will be sorely missed.

AUTHOR CONTRIBUTIONS

K.C. and C.C.D. performed the experiments and designed the research. A.E.Z., J.J., and T.L.C. contributed to immunohistochemical (IHC) analysis, qPCR, and cell culture. K.C. and M.P.S.-B. performed ChIP. A.M.S. performed pathological analysis. A.M.S., A.F., L.J.J., and S.S. provided patient material. O.B. provided GSK591. J.Z. and E.G. provided bioinformatic support. G.F. and M.J.S. advised on experiments and the manuscript. C.C.D. wrote the manuscript.

DECLARATION OF INTERESTS

The authors declare no competing interests.

Received: June 8, 2017
Revised: November 1, 2017
Accepted: November 28, 2017
Published: December 19, 2017

REFERENCES

Al-Hajj, M., Wicha, M.S., Benito-Hernandez, A., Morrison, S.J., and Clarke, M.F. (2003). Prospective identification of tumorigenic breast cancer cells. Proc. Natl. Acad. Sci. U S A *100*, 3983–3988.

Baldwin, R.M., Morettin, A., Paris, G., Goulet, I., and Côté, J. (2012). Alternatively spliced protein arginine methyltransferase 1 isoform PRMT1v2 promotes the survival and invasiveness of breast cancer cells. Cell Cycle *11*, 4597–4612.

Banasavadi-Siddegowda, Y.K., Russell, L., Frair, E., Karkhanis, V.A., Relation, T., Yoo, J.Y., Zhang, J., Sif, S., Imitola, J., Baiocchi, R., and Kaur, B. (2017). PRMT5-PTEN molecular pathway regulates senescence and self-renewal of primary glioblastoma neurosphere cells. Oncogene *36*, 263–274.

Bezzi, M., Teo, S.X., Muller, J., Mok, W.C., Sahu, S.K., Vardy, L.A., Bonday, Z.Q., and Guccione, E. (2013). Regulation of constitutive and alternative

splicing by PRMT5 reveals a role for Mdm4 pre-mRNA in sensing defects in the spliceosomal machinery. Genes Dev. *27*, 1903–1916.

Blanc, R.S., Vogel, G., Li, X., Yu, Z., Li, S., and Richard, S. (2017). Arginine methylation by PRMT1 regulates muscle stem cell fate. Mol. Cell. Biol. *37*, e00457-16.

Brown, P.J., Ashe, S.L., Leich, E., Burek, C., Barrans, S., Fenton, J.A., Jack, A.S., Pulford, K., Rosenwald, A., and Banham, A.H. (2008). Potentially oncogenic B-cell activation-induced smaller isoforms of FOXP1 are highly expressed in the activated B cell-like subtype of DLBCL. Blood *111*, 2816–2824.

Cerami, E., Gao, J., Dogrusoz, U., Gross, B.E., Sumer, S.O., Aksoy, B.A., Jacobsen, A., Byrne, C.J., Heuer, M.L., Larsson, E., et al. (2012). The cBio cancer genomics portal: an open platform for exploring multidimensional cancer genomics data. Cancer Discov. *2*, 401–404.

Chan-Penebre, E., Kuplast, K.G., Majer, C.R., Boriack-Sjodin, P.A., Wigle, T.J., Johnston, L.D., Rioux, N., Munchhof, M.J., Jin, L., Jacques, S.L., et al. (2015). A selective inhibitor of PRMT5 with in vivo and in vitro potency in MCL models. Nat. Chem. Biol. *11*, 432–437.

Chang, C.-J., Yang, J.-Y., Xia, W., Chen, C.-T., Xie, X., Chao, C.-H., Woodward, W.A., Hsu, J.-M., Hortobagyi, G.N., and Hung, M.-C. (2011). EZH2 promotes expansion of breast tumor initiating cells through activation of RAF1-β-catenin signaling. Cancer Cell *19*, 86–100.

Chen, H., Lorton, B., Gupta, V., and Shechter, D. (2017). A TGFβ-PRMT5-MEP50 axis regulates cancer cell invasion through histone H3 and H4 arginine methylation coupled transcriptional activation and repression. Oncogene *36*, 373–386.

Chittka, A., Nitarska, J., Grazini, U., and Richardson, W.D. (2012). Transcription factor positive regulatory domain 4 (PRDM4) recruits protein arginine methyltransferase 5 (PRMT5) to mediate histone arginine methylation and control neural stem cell proliferation and differentiation. J. Biol. Chem. *287*, 42995–43006.

Choi, E.J., Seo, E.J., Kim, D.K., Lee, S.-I., Kwon, Y.W., Jang, I.H., Kim, K.-H., Suh, D.-S., and Kim, J.H. (2016). FOXP1 functions as an oncogene in promoting cancer stem cell-like characteristics in ovarian cancer cells. Oncotarget *7*, 3506–3519.

Clarke, T.L., Sanchez-Bailon, M.P., Chiang, K., Reynolds, J.J., Herrero-Ruiz, J., Bandeiras, T.M., Matias, P.M., Maslen, S.L., Skehel, J.M., Stewart, G.S., and Davies, C.C. (2017). PRMT5-dependent methylation of the TIP60 coactivator RUVBL1 is a key regulator of homologous recombination. Mol. Cell *65*, 900–916.e7.

Dacwag, C.S., Ohkawa, Y., Pal, S., Sif, S., and Imbalzano, A.N. (2007). The protein arginine methyltransferase Prmt5 is required for myogenesis because it facilitates ATP-dependent chromatin remodeling. Mol. Cell. Biol. *27*, 384–394.

Di Lorenzo, A., and Bedford, M.T. (2011). Histone arginine methylation. FEBS Lett. *585*, 2024–2031.

Duncan, K.W., Rioux, N., Boriack-Sjodin, P.A., Munchhof, M.J., Reiter, L.A., Majer, C.R., Jin, L., Johnston, L.D., Chan-Penebre, E., Kuplast, K.G., et al. (2015). Structure and property guided design in the identification of PRMT5 tool compound EPZ015666. ACS Med. Chem. Lett. *7*, 162–166.

Feinberg, A.P., Koldobskiy, M.A., and Göndör, A. (2016). Epigenetic modulators, modifiers and mediators in cancer aetiology and progression. Nat. Rev. Genet. *17*, 284–299.

Fillmore, C.M., and Kuperwasser, C. (2008). Human breast cancer cell lines contain stem-like cells that self-renew, give rise to phenotypically diverse progeny and survive chemotherapy. Breast Cancer Res. *10*, R25.

Gabut, M., Samavarchi-Tehrani, P., Wang, X., Slobodeniuc, V., O'Hanlon, D., Sung, H.-K., Alvarez, M., Talukder, S., Pan, Q., Mazzoni, E.O., et al. (2011). An alternative splicing switch regulates embryonic stem cell pluripotency and reprogramming. Cell *147*, 132–146.

Gao, J., Aksoy, B.A., Dogrusoz, U., Dresdner, G., Gross, B., Sumer, S.O., Sun, Y., Jacobsen, A., Sinha, R., Larsson, E., et al. (2013). Integrative analysis of complex cancer genomics and clinical profiles using the cBioPortal. Sci. Signal. *6*, pl1.

Ginestier, C., Hur, M.-H., Charafe-Jauffret, E., Monville, F., Dutcher, J., Brown, M., Jacquemier, J., Viens, P., Kleer, C.G., Liu, S., et al. (2007). ALDH1 is a marker of normal and malignant human mammary stem cells and a predictor of poor clinical outcome. Cell Stem Cell *1*, 555–567.

Goulet, I., Gauvin, G., Boisvenue, S., and Côté, J. (2007). Alternative splicing yields protein arginine methyltransferase 1 isoforms with distinct activity, substrate specificity, and subcellular localization. J. Biol. Chem. *282*, 33009–33021.

Goyal, A., Chavez, S.L., and Reijo Pera, R.A. (2013). Generation of human induced pluripotent stem cells using epigenetic regulators reveals a germ cell-like identity in partially reprogrammed colonies. PLoS ONE *8*, e82838.

Grebien, F., Vedadi, M., Getlik, M., Giambruno, R., Grover, A., Avellino, R., Skucha, A., Vittori, S., Kuznetsova, E., Smil, D., et al. (2015). Pharmacological targeting of the Wdr5-MLL interaction in C/EBPα N-terminal leukemia. Nat. Chem. Biol. *11*, 571–578.

Györffy, B., Lánczky, A., Eklund, A.C., Denkert, C., Budczies, J., Li, Q., and Szállási, Z. (2010). An online survival analysis tool to rapidly assess the effect of 22,277 genes on breast cancer prognosis using microarray data of 1,809 patients. Breast Cancer Res. Treat. *123*, 725–731.

Harrison, H., Farnie, G., Howell, S.J., Rock, R.E., Stylianou, S., Brennan, K.R., Bundred, N.J., and Clarke, R.B. (2010). Regulation of breast cancer stem cell activity by signaling through the Notch4 receptor. Cancer Res. *70*, 709–718.

Holohan, C., Van Schaeybroeck, S., Longley, D.B., and Johnston, P.G. (2013). Cancer drug resistance: an evolving paradigm. Nat. Rev. Cancer *13*, 714–726.

Huang, D.W., Sherman, B.T., and Lempicki, R.A. (2009). Systematic and integrative analysis of large gene lists using DAVID Bioinformatics Resources. Nature Protoc. *4*, 44–57.

Jin, Y., Zhou, J., Xu, F., Jin, B., Cui, L., Wang, Y., Du, X., Li, J., Li, P., Ren, R., and Pan, J. (2016). Targeting methyltransferase PRMT5 eliminates leukemia stem cells in chronic myelogenous leukemia. J. Clin. Invest. *126*, 3961–3980.

Liu, F., Cheng, G., Hamard, P.-J., Greenblatt, S., Wang, L., Man, N., Perna, F., Xu, H., Tadi, M., Luciani, L., and Nimer, S.D. (2015). Arginine methyltransferase PRMT5 is essential for sustaining normal adult hematopoiesis. J. Clin. Invest. *125*, 3532–3544.

Migliori, V., Müller, J., Phalke, S., Low, D., Bezzi, M., Mok, W.C., Sahu, S.K., Gunaratne, J., Capasso, P., Bassi, C., et al. (2012). Symmetric dimethylation of H3R2 is a newly identified histone mark that supports euchromatin maintenance. Nat. Struct. Mol. Biol. *19*, 136–144.

Muñoz, P., Iliou, M.S., and Esteller, M. (2012). Epigenetic alterations involved in cancer stem cell reprogramming. Mol. Oncol. *6*, 620–636.

Nagamatsu, G., Kosaka, T., Kawasumi, M., Kinoshita, T., Takubo, K., Akiyama, H., Sudo, T., Kobayashi, T., Oya, M., and Suda, T. (2011). A germ cell-specific gene, Prmt5, works in somatic cell reprogramming. J. Biol. Chem. *286*, 10641–10648.

Naudin, C., Hattabi, A., Michelet, F., Miri-Nezhad, A., Benyoucef, A., Pflumio, F., Guillonneau, F., Fichelson, S., Vigon, I., Dusanter-Fourt, I., and Lauret, E. (2017). PUMILIO/FOXP1 signaling drives expansion of hematopoietic stem/progenitor and leukemia cells. Blood *129*, 2493–2506.

Oskay Halacli, S. (2017). FOXP1 enhances tumor cell migration by repression of NFAT1 transcriptional activity in MDA-MB-231 cells. Cell Biol. Int. *41*, 102–110.

Pal, S., Vishwanath, S.N., Erdjument-Bromage, H., Tempst, P., and Sif, S. (2004). Human SWI/SNF-associated PRMT5 methylates histone H3 arginine 8 and negatively regulates expression of ST7 and NM23 tumor suppressor genes. Mol. Cell. Biol. *24*, 9630–9645.

Ponti, D., Costa, A., Zaffaroni, N., Pratesi, G., Petrangolini, G., Coradini, D., Pilotti, S., Pierotti, M.A., and Daidone, M.G. (2005). Isolation and in vitro propagation of tumorigenic breast cancer cells with stem/progenitor cell properties. Cancer Res. *65*, 5506–5511.

Powers, M.A., Fay, M.M., Factor, R.E., Welm, A.L., and Ullman, K.S. (2011). Protein arginine methyltransferase 5 accelerates tumor growth by arginine

methylation of the tumor suppressor programmed cell death 4. Cancer Res. *71*, 5579–5587.

Scoumanne, A., Zhang, J., and Chen, X. (2009). PRMT5 is required for cell-cycle progression and p53 tumor suppressor function. Nucleic Acids Res. *37*, 4965–4976.

Shigekawa, T., Ijichi, N., Ikeda, K., Horie-Inoue, K., Shimizu, C., Saji, S., Aogi, K., Tsuda, H., Osaki, A., Saeki, T., and Inoue, S. (2011). FOXP1, an estrogen-inducible transcription factor, modulates cell proliferation in breast cancer cells and 5-year recurrence-free survival of patients with tamoxifen-treated breast cancer. Horm. Cancer *2*, 286–297.

Tam, W.L., and Weinberg, R.A. (2013). The epigenetics of epithelial-mesenchymal plasticity in cancer. Nat. Med. *19*, 1438–1449.

Tee, W.-W., Pardo, M., Theunissen, T.W., Yu, L., Choudhary, J.S., Hajkova, P., and Surani, M.A. (2010). Prmt5 is essential for early mouse development and acts in the cytoplasm to maintain ES cell pluripotency. Genes Dev. *24*, 2772–2777.

Wu, Y., Wang, Y., Yang, X.H., Kang, T., Zhao, Y., Wang, C., Evers, B.M., and Zhou, B.P. (2013). The deubiquitinase USP28 stabilizes LSD1 and confers stem-cell-like traits to breast cancer cells. Cell Rep. *5*, 224–236.

Xiao, J., He, B., Zou, Y., Chen, X., Lu, X., Xie, M., Li, W., He, S., You, S., and Chen, Q. (2016). Prognostic value of decreased FOXP1 protein expression in various tumors: a systematic review and meta-analysis. Sci. Rep. *6*, 30437.

Yang, F., Wang, J., Ren, H.-Y., Jin, J., Wang, A.-L., Sun, L.-L., Diao, K.-X., Wang, E.-H., and Mi, X.-Y. (2015). Proliferative role of TRAF4 in breast cancer by upregulating PRMT5 nuclear expression. Tumour Biol. *36*, 5901–5911.

Zhang, T., Günther, S., Looso, M., Künne, C., Krüger, M., Kim, J., Zhou, Y., and Braun, T. (2015). Prmt5 is a regulator of muscle stem cell expansion in adult mice. Nat. Commun. *6*, 7140.

Zhao, Q., Rank, G., Tan, Y.T., Li, H., Moritz, R.L., Simpson, R.J., Cerruti, L., Curtis, D.J., Patel, D.J., Allis, C.D., et al. (2009). PRMT5-mediated methylation of histone H4R3 recruits DNMT3A, coupling histone and DNA methylation in gene silencing. Nat. Struct. Mol. Biol. *16*, 304–311.

Cell Reports, Volume *21*

Supplemental Information

PRMT5 Is a Critical Regulator of Breast Cancer Stem Cell Function via Histone Methylation and FOXP1 Expression

Kelly Chiang, Agnieszka E. Zielinska, Abeer M. Shaaban, Maria Pilar Sanchez-Bailon, James Jarrold, Thomas L. Clarke, Jingxian Zhang, Adele Francis, Louise J. Jones, Sally Smith, Olena Barbash, Ernesto Guccione, Gillian Farnie, Matthew J. Smalley, and Clare C. Davies

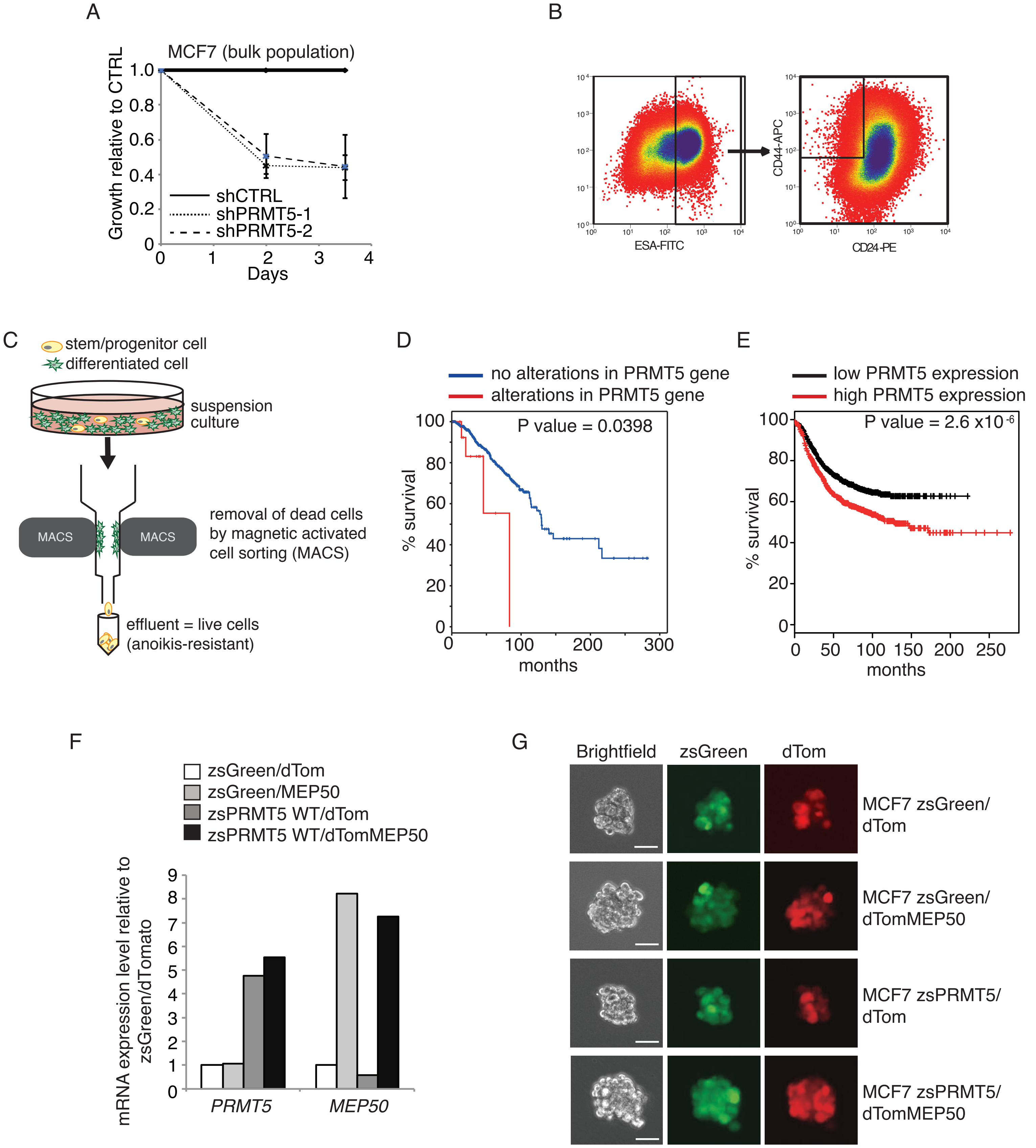

Supplemental Figure 1, related to Figure 1: PRMT5 is required for breast cancer maintenance. **(A)** Proliferation of MCF7 shCTRL, shPRMT5-1 (hairpin1) or shPRMT5-2 (hairpin2) cells was assessed by MTT assay. **(B)** FACS-sorting methodology. Cells were stained with ESA-FITC, CD24-PE and CD44-APC and gated for $ESA^{+}/CD24^{low}/CD44^{+}$. **(C)** Schematic for the isolation of anoikis-resistant cells. **(D)** Kaplan-Meier (KM) plot of % survival for alterations in PRMT5. Data taken from cBioportal.org (TCGA, 2015 dataset, 816 cases). 2% of cases exhibit alterations in the PRMT5 gene; 11 amplification, 4 missense and 1 in-frame mutation. **(E)** KM plot for survival of breast cancer patients with low and high PRMT5 levels. Data taken from http://kmplot.com/analysis/ with filters set to “all” breast cancers using probe 1564520_s_at. **(F)** qPCR for PRMT5 and MEP50 gene expression in engineered MCF7 cells. (G) Microscope images of mammospheres derived from MCF7 cells engineered to overexpress PRMT5 and MEP50. Scale bar = 50μm.

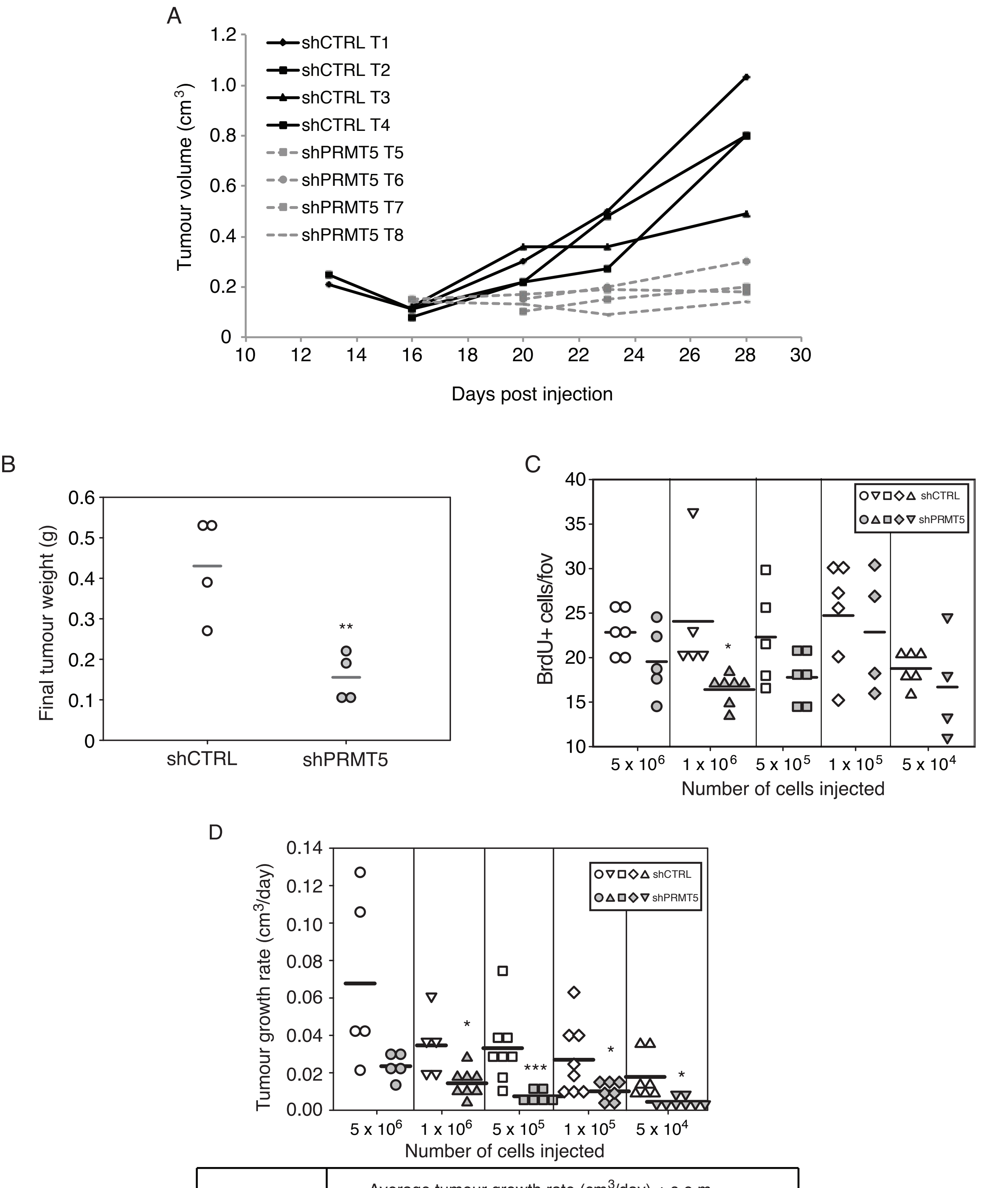

	Average tumour growth rate (cm^3/day) ± s.e.m.				
# cells injected	5 x 10^6	1 x 10^6	5 x 10^5	1 x 10^5	5 x 10^4
shCTRL	0.07 ± 0.02	0.03 ± 0.01	0.03 ± 0.01	0.03 ± 0.01	0.02 ± 0.005
shPRMT5	0.02 ± 0.003	0.01 ± 0.003	0.01 ± 0.001	0.01 ± 0.002	0.004 ± 0.001

Supplemental Figure 2, related to Figure 2: PRMT5 is required for self-renewal and tumour growth *in vivo*.
Data shown in (A) and (B) are from a specific cohort of mice (Group 3 - 5x10^5 cells) from one limiting dilution experiment as shown in Figure 2B. **(A)** Growth of tumours from mice injected with 5 x 10^5 cells was monitored by caliper measurements at the times indicated and plotted as tumour volume (cm^3) over time. T denotes an individual tumour. T1-4 denote shCTRL tumours (solid black lines). T5-8 denote shPRMT5 tumours (dotted grey lines). **(B)** Final tumour weight (g) of tumours derived from shCTRL or shPRMT5-expressing cells in (A). Bar = mean, ** $p < 0.01$ Student's *t*-test. **(C)** Dot density plot of $BrdU^+$ /total number of cells in field of view (fov) from shCTRL and shPRMT5 tumours. **(D)** Average tumour growth rate of tumours from the limiting dilution assay shown in Figure 2B.

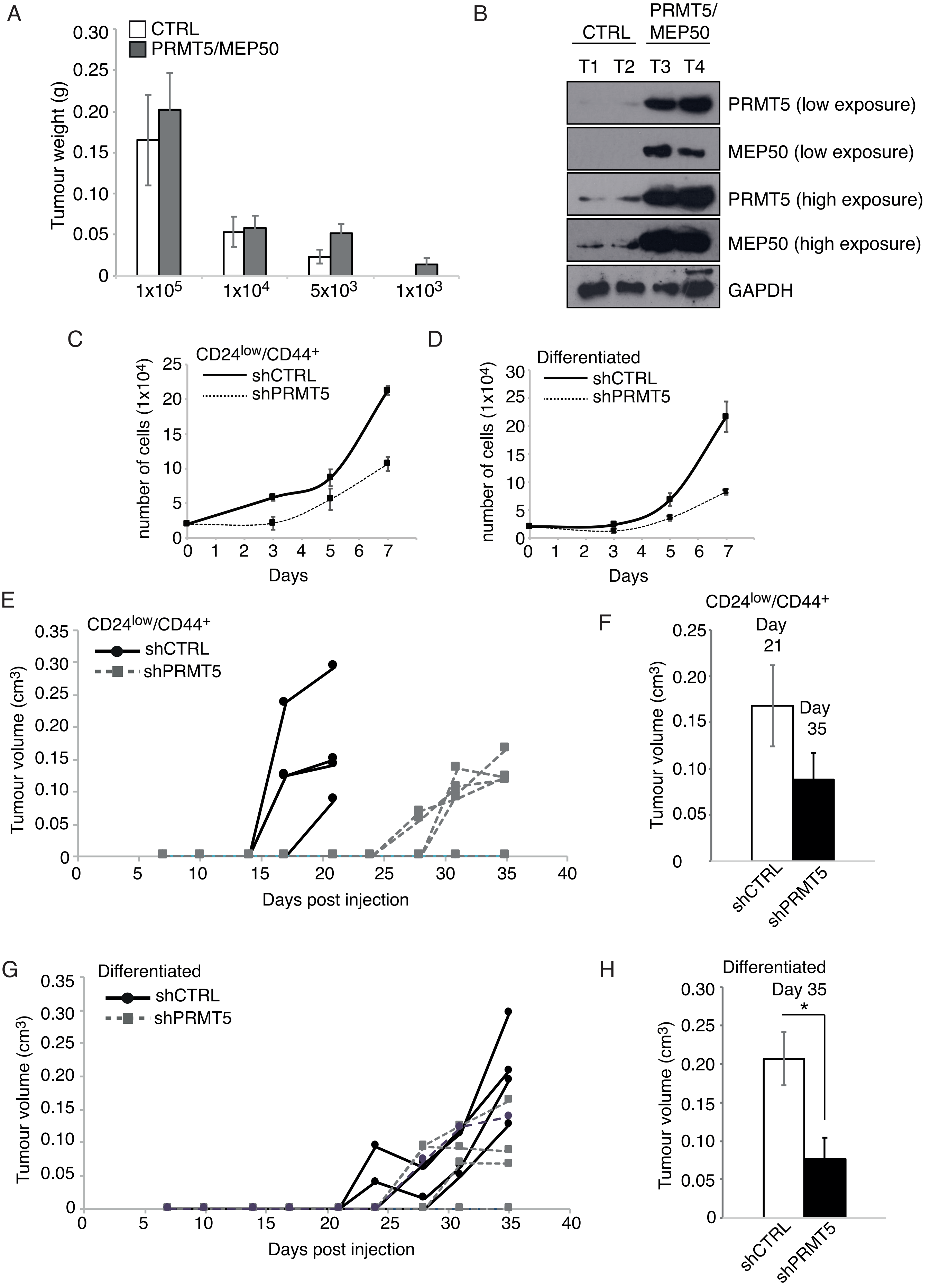

Supplemental Figure 3, related to Figure 2: PRMT5 is required for BCSC and bulk tumour function *in vivo.*
(A) Average weight of tumours derived from PRMT5/MEP50 or control cells from the limiting dilution assay shown in Figure 2G. **(B)** PRMT5 and MEP50 overexpression was assessed in tumours derived from the limiting dilution assay. T1 and T2 are denote individual control tumours and T3 and T4 denote individual tumours derived from PRMT5/MEP50 overexpressing cells. Proliferation of shCTRL or shPRMT5 **(C)** ESA$^+$/CD24^{low}/CD44$^+$ or **(D)** differentiated cells was determined by counting on a haemocytometer. MCF7 shCTRL or shPRMT5 ESA$^+$/CD24^{low}/CD44$^+$ or differentiated cells were FACS sorted and injected into NSG mice. **(E)** Growth of tumours derived from shCTRL or shPRMT5 EESA$^+$/CD24^{low}/CD44$^+$ cells. **(F)** Average final volume of tumours derived from shCTRL or shPRMT5 ESA$^+$/CD24^{low}/CD44$^+$ cells. **(G)** Growth of tumours derived from shCTRL or shPRMT5 differentiated cells. **(H)** Average final volume of tumours derived from shCTRL or shPRMT5 differentiated cells.

A

Injected cells	Slide number	Grade	Cellularity	Tubules	Pleomorphism	Mitosis	Fibrous Tissue	Necrosis (%)
MCF7 shCTRL	135/F3/L	3	high	3	2	3	min	nd
MCF7 shCTRL	135/F3/R	2	mod	3	2	2	mod	nd
MCF7 shCTRL	135/F5/P5/L	nd	nd	nd	nd	nd	nd	nd
MCF7 shCTRL	135/F5/P5/R	nd	nd	nd	nd	nd	nd	nd
MCF7 shCTRL	24/F4/R	3	high	3	3	3	min	0
MCF7 shCTRL	24/F5	3	high	3	3	3	min	30
MCF7 shCTRL	27/F1/L	3	high	3	3	3	min	25
MCF7 shCTRL	27/F1/R	3	high	3	3	3	min	20
MCF7 shCTRL	27/F2/L	3	high	3	3	3	min	40
MCF7 shCTRL	27/F2/R	3	high	3	3	3	min	60
MCF7 shCTRL	27/F3/L	3	high	3	3	3	min	5
MCF7 shCTRL	27/F3/R	3	high	3	3	3	min	25
MCF7 shCTRL	27/F4/L	3	high	3	3	3	low	0
MCF7 shCTRL	27/F4/R	3	high	3	3	3	min	10
MCF7 shCTRL	54/F3/L	3	high	3	3	3	low	65
MCF7 shCTRL	54/F3/R	3	high	3	3	3	min	0
MCF7 shCTRL	54/F4/L	3	high	3	3	3	min	45
MCF7 shPRMT5	136/F3/C/L	3	very high	3	3	3	none	20
MCF7 shPRMT5	136/F3/C/R	nd	nd	nd	nd	nd	nd	40
MCF7 shPRMT5	160/F3/L	3	mod	2	3	3	mod	0.1
MCF7 shPRMT5	160/F3/R	3	high	3	3	3	min	0.05
MCF7 shPRMT5	160/F5/L	3	mod	2	3	3	mod	0
MCF7 shPRMT5	160/F5/R	3	mod	2	3	3	mod-high	0
MCF7 shPRMT5	162/F2/L	3	high	2	3	3	mod	0
MCF7 shPRMT5	186/F2/L	3	high	3	3	3	low	10
MCF7 shPRMT5	186/F2/R	3	high	3	3	3	mod	<5
MCF7 shPRMT5	186/F3/L	3	mod	2	3	3	mod	5
MCF7 shPRMT5	186/F3/R	3	mod	2	3	3	mod	<5
MCF7 shPRMT5	186/F5/L	3	mod	2	3	3	mod	10
MCF7 shPRMT5	186/F5/R	3	mod	2	3	3	mod	40
MCF7 shPRMT5	24/F1/L	3	mod	2	3	3	mod	<5
MCF7 shPRMT5	24/F1/R	3	high	2	3	3	min	0

B

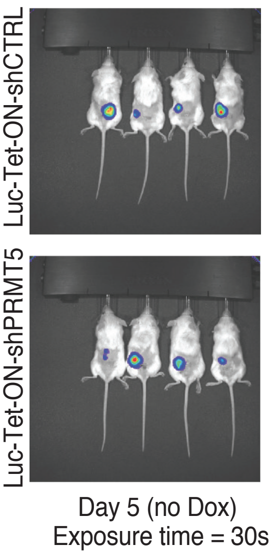

C

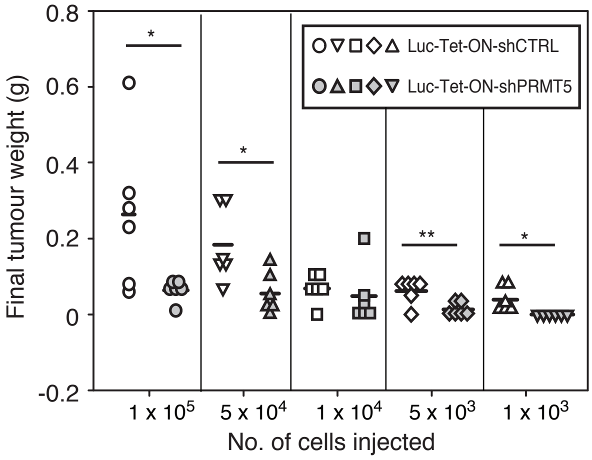

D

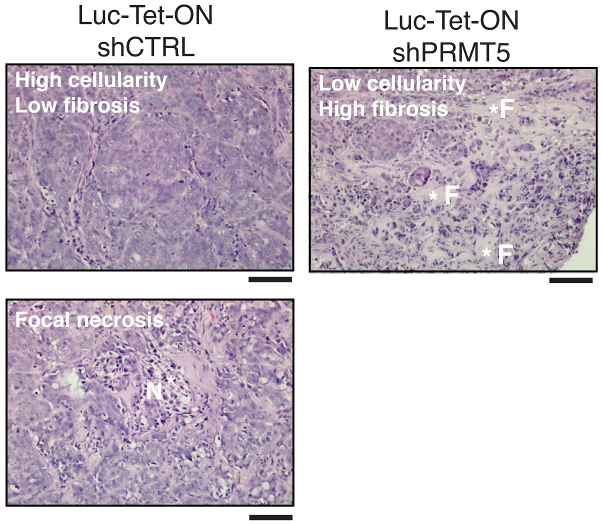

E

Injected cells	Slide number	Grade	Cellularity	Tubules	Pleomorphism	Mitosis	Fibrous Tissue	Necrosis (%)
MCF7 Luc tet-ON shCTRL	137/F2/L	3	high	3	3	3	low	0
MCF7 Luc tet-ON shCTRL	137/F2/R	3	high	3	3	2	low	0
MCF7 Luc tet-ON shCTRL	137/F6/L	3	high	3	3	2	mod	2% focal
MCF7 Luc tet-ON shCTRL	143/F1/L	3	high	3	3	2	none	0
MCF7 Luc tet-ON shCTRL	143/F1/R	3	high	3	3	3	none	0.3
MCF7 Luc tet-ON shCTRL	143/F3/L	2	mod	2	3	2	mod-high	0
MCF7 Luc tet-ON shCTRL	143/F3/R	3	high	3	3	2	min	0
MCF7 Luc tet-ON shCTRL	150/F6/L	2	mod	2	3	2	mod	0
MCF7 Luc tet-ON shCTRL	150/F6/R	3	high	3	3	3	min (focal)	0
MCF7 Luc tet-ON shCTRL	156/F5/L	3	mod	3	3	3	mod	0.05
MCF7 Luc tet-ON shCTRL	156/F5/R	3	mod	3	3	3	min	0
MCF7 Luc tet-ON shPRMT5	137/F5/L	3	mod	3	3	2	mod	0
MCF7 Luc tet-ON shPRMT5	137/F5/R	3	mod	3	3	2	mod	0
MCF7 Luc tet-ON shPRMT5	143/F2/L	1	v low	1	3	1	90% v high	0
MCF7 Luc tet-ON shPRMT5	143/F2/R	3	high	3	3	3	none	0
MCF7 Luc tet-ON shPRMT5	143/F4/R	2	mod	2	3	1	mod	0
MCF7 Luc tet-ON shPRMT5	152/F1/L	2	mod	2	3	2	mod	0
MCF7 Luc tet-ON shPRMT5	152/F2/R	2	mod	2	3	2	mod	0

Supplemental Figure 4, related to Figure 3 and 5: Depletion of PRMT5 reduces tumour aggressiveness.
(A) Table of pathological features of shCTRL and shPRMT5 tumour sections from limiting dilution assay shown in Figure 2B. **(B)** IVIS imaging to confirm tumour growth prior to *in vivo* doxycycline-induced depletion of PRMT5. **(C)** Dot density plot of final tumour weight from limiting dilution assay of MCF7 Luc-TET-ON-shCTRL or shPRMT5 cells. Bar = mean, * $p < 0.05$, ** $p < 0.01$ Student's *t*-test. **(D)** Representative images of pathological features from Luc Tet-ON-shCTRL or shPRMT5 tumours. F = fibrous tissue, N = necrotic area. Scale bar = 100µm. **(E)** Table of pathological features of MCF7 Luc-Tet-ON shCTRL and shPRMT5 tumour sections.

A

Genes suppressed by PRMT5

**ABCC11**	12.69	EDN1	3.92	S100A8	3.05	RAMP3	2.64	BDKRB2	2.43	NFKBIZ	2.19	ANKRD50	1.98
**SLC4A10**	9.91	COL4A5	3.87	ACKR3	3.02	CRISPLD1	2.63	ACACB	2.38	LGALS3BP	2.16	PALLD	1.98
**CLCA2**	8.15	COL12A1	3.83	ATF3	2.99	PIK3IP1	2.61	TFPI2	2.35	STMN3	2.16	GDF15	1.97
**MALRD1**	7.87	MAPK8IP2	3.69	ADCY5	2.99	QPRT	2.61	KIF1A	2.35	USP40	2.16	ALG10	1.97
**ABCA12**	6.48	SLC6A14	3.58	**KITLG**	2.99	GABARAPL1	2.59	**CDKN1A**	2.34	PMP22	2.15	S100A9	1.97
**COL3A1**	6.30	KYNU	3.51	METTL7A	2.97	ELF5	2.59	NTN4	2.34	DOCK10	2.14	TIMP2	1.95
CMAHP	6.22	SH3TC1	3.49	SLC12A4	2.92	MTRNR2L10	2.57	ITGB6	2.33	ANXA6	2.14	NCAM2	1.94
**COL21A1**	6.07	TIMP3	3.43	IGSF1	2.83	PHLDB2	2.56	MAP3K1	2.33	EFHD1	2.14	UGCG	1.93
**ERBB4**	5.93	COL5A1	3.43	NYNRIN	2.80	AKR1C1	2.55	ARHGAP29	2.32	FAXDC2	2.11	SEMA3E	1.93
**NPY1R**	5.89	GFRA1	3.39	SYNPO2	2.79	DBN1	2.54	LIFR	2.32	TMPRSS2	2.11	MDM2	1.93
PLXDC2	5.42	EHF	3.35	MAP1B	2.77	ELP2	2.54	ADAMTS9	2.32	PARM1	2.10	JUN	1.91
ARHGAP36	5.13	BNIPL	3.24	SESN3	2.76	CTSF	2.52	CRABP2	2.27	TMEM63C	2.08	SSFA2	1.90
CDH18	5.12	ST8SIA4	3.17	CAB39L	2.73	NGEF	2.49	ATP2B4	2.26	DBP	2.07	GNA12	1.89
OLFML3	4.96	AMIGO2	3.16	WNT5A	2.70	KIAA1407	2.47	INSR	2.26	CLSTN2	2.05	**PRICKLE2**	1.89
C16orf45	4.89	SLC39A6	3.15	FAM129A	2.69	MTRNR2L8	2.47	ETNK2	2.26	KLHL24	2.04	MARCKS	1.84
L1CAM	4.67	RGS22	3.14	KLF6	2.69	HPGD	2.45	FBLIM1	2.22	COX6C	2.04	MAN1A1	1.84
SHISA2	4.52	ACSS1	3.12	LDHB	2.68	LOC374443	2.44	PEG10	2.21	ALG10B	2.03		
PTGER4	4.28	ARMCX2	3.12	ALDH3B2	2.67	FSCN1	2.44	SYT1	2.20	PLCG2	2.01		
PDK4	4.25	SYT10	3.10	SCARA3	2.66	CLSTN3	2.43	DUSP8	2.19	IRS2	2.00		
NAALADL2	4.10	RPS18P9	3.07	PAPSS2	2.66	MTRNR2L6	2.43	MAOA	2.19	SLC7A2	2.00		

Genes activated by PRMT5

**LOX**	0.13	HSH2D	0.33	IFITM10	0.42	SLC29A3	0.48
**C1orf116**	0.15	SOX21-AS1	0.34	NOL3	0.43	FAM171B	0.49
**SCIN**	0.16	FGD3	0.35	PDK1	0.43	TSTA3	0.49
**KRT81**	0.16	**LRP4**	0.36	PLXNA2	0.43	SLC39A4	0.49
PPP1R3G	0.17	ARID5A	0.37	SLC9A2	0.43	COBLL1	0.49
**CRAT**	0.19	SLC45A3	0.37	**HK2**	0.44	HIST1H2AH	0.50
**MAB21L3**	0.21	CEACAM5	0.38	PKIB	0.44	**TPD52**	0.50
**MPPED2**	0.23	EHD3	0.39	NR5A2	0.45	STARD10	0.50
**RIMKLA**	0.25	HOXA13	0.39	ABHD17C	0.45	SPDEF	0.50
**ADM**	0.25	FUT11	0.39	GPC6	0.45	EIF5B	0.51
MUCL1	0.25	RAB3B	0.39	ITGA6	0.45	ANKRD36	0.51
**NDRG1**	0.26	MLKL	0.39	GOLM1	0.45	COX5B	0.51
SERPINB5	0.29	ALDOC	0.40	KANK1	0.46	FIGN	0.51
FAM13A	0.30	MUC5B	0.40	CA2	0.47	**FOXP1**	0.52
CAPN9	0.30	EGLN3	0.41	ZFP36L2	0.47	SNRPC	0.53
**PRMT5**	0.31	TOX3	0.41	PTPN18	0.47	PPIF	0.53
SOX21	0.32	ABLIM1	0.41	NEB	0.47	STARD7	0.54
EML5	0.33	PPFIA4	0.41	HIST1H2BB	0.48	PFN1	0.56
PRODH	0.33	GALNT12	0.41	NCAPH	0.48		
ALDH3A1	0.33	BCAS1	0.42	AGR2	0.48		

B

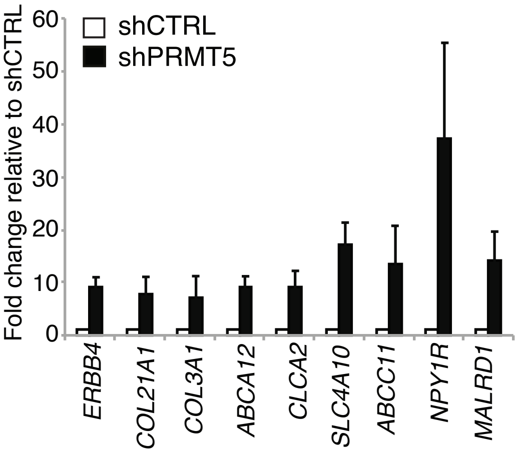

C

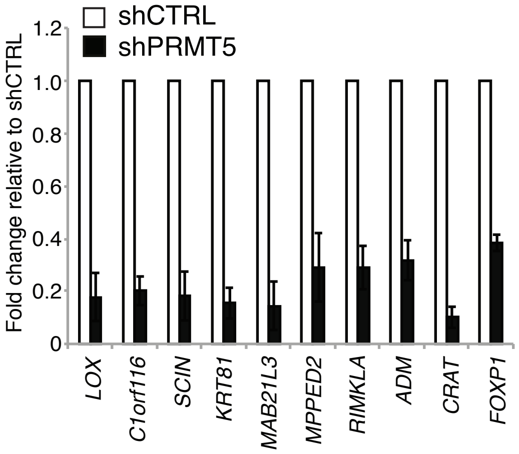

D

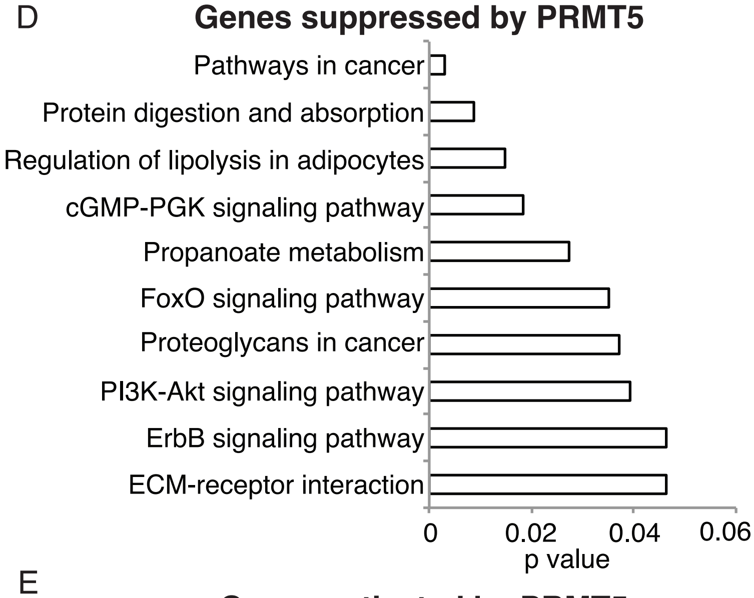

E

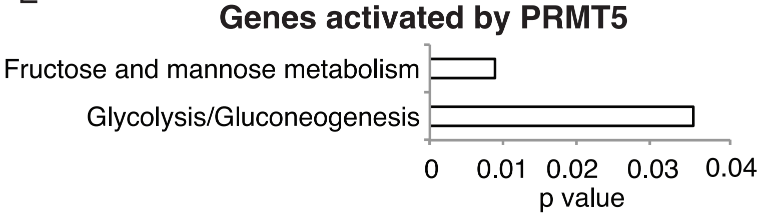

Supplemental Figure 5, related to Figure 6: PRMT5 regulates gene expression in BCSCs.
(A) Table of PRMT5-regulated genes in BCSCs. Three independent RNA samples were isolated from MCF7 shCTRL or shPRMT5 ESA^{+}/CD24^{low}/CD44^{+} cells. Number of reads for each sample are shCTRL 1: 31,997,304, shCTRL 2: 44,222,799, shCTRL 3: 13,885,650, shPRMT5 1: 39,039,672, shPRMT5 2: 132,754,382 (sample resequenced hence higher read number) and shPRMT5 3: 14,209,470. Samples were paired-end sequenced and aligned to the human hg19 genome. Differential gene expression was determined using Cuffdiff 2.2.1 with a threshold of FDR < 0.05 and fold change > 1.5. Fold change of genes that were significantly upregulated following PRMT5 knockdown (hence, suppressed by PRMT5) (red) and downregulated following PRMT5 knockdown (activated by PRMT5) (blue) are shown. Validation of top 10 differentially **(B)** PRMT5-suppressed and **(C)** PRMT5-activated genes, as highlighted in bold in (A), was performed by qPCR. *FOXP1* is not a top10 gene but is included for comparison. n=3, error = s.e.m. KEGG pathway analysis of **(D)** genes suppressed by PRMT5 and **(E)** genes activated by PRMT5.

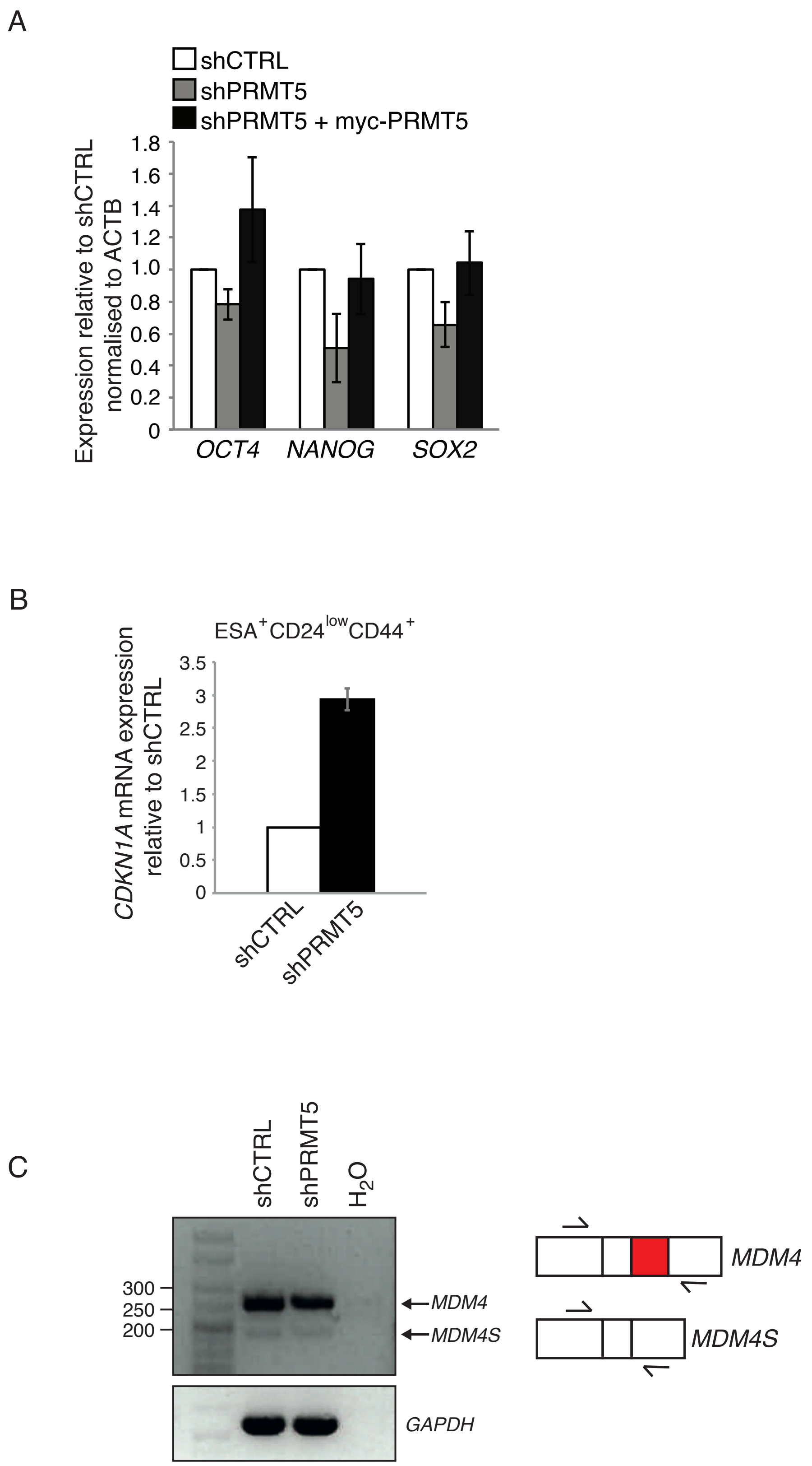

Supplemental Figure 6, related to Figure 6: Analysis of differentially regulated genes following PRMT5 depletion in BCSCs. **(A)** Quantitative PCR (qPCR) for *OCT4, NANOG* and *SOX2* in MCF7 shCTRL, shPRMT5 or shPRMT5 anoikis-resistant cells reconstituted with ectopically overexpressed PRMT5. Gene expression is expressed relative to shCTRL (n=3). **(B)** Expression of CDKN1A in $ESA^{+}/CD24^{low}/CD44^{+}$ cells was assessed by qPCR following PRMT5 depletion (n=3). **(C)** Analysis of MDM4 splicing following PRMT5 depletion in anoikis-resistant cells. MDM4 = full length isoform, MDM4S = short isoform as a result of exon skipping.

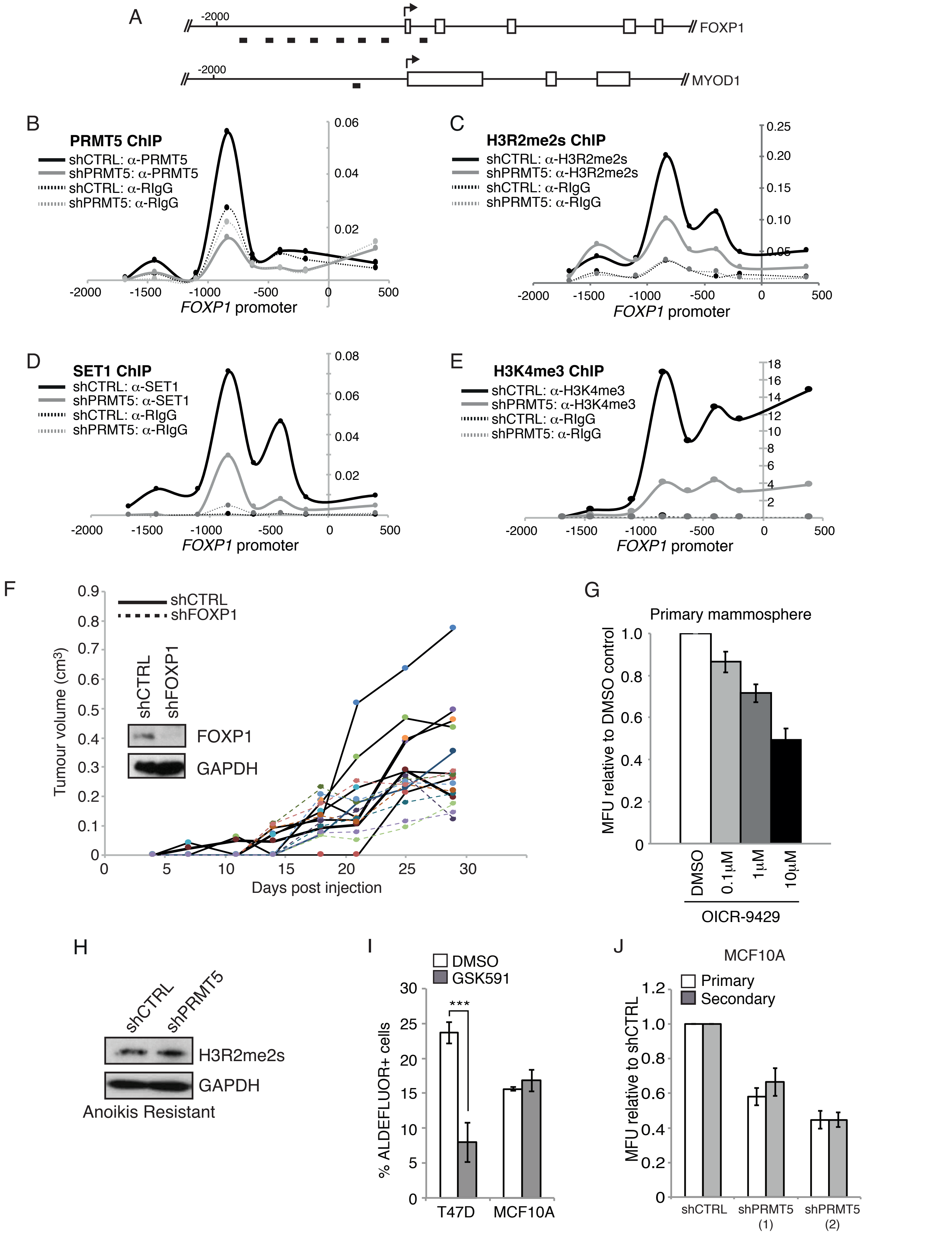

Supplemental Figure 7, related to Figure 6 and 7: PRMT5 epigenetically regulates FOXP1 gene expression contributing to tumour growth.
(A) Schematic representation of FOXP1 and MYOD1 promoter regions. Thick black bars show tiling primers used to assess enrichment at these loci. Tiling ChIP-qPCR of **(B)** PRMT5, **(C)** H3R2me2s, **(D)** SET1, **(E)** H3K4me3 enrichment across the FOXP1 promoter region. **(F)** Growth of tumours derived from shCTRL or shFOXP1 cells. FOXP1 levels in MCF7 shCTRL or shFOXP1 cells were assessed prior to injection (inset). **(G)** Mammosphere assay of MCF7 cells following WDR5 inhibition by OICR-9429. **(H)** Immunoblot for H3R2me2 levels in anoikis resistant cells. **(I)** ALDEFLUOR assay of T47D and MCF10A cells ± 5μM GSK591. **(J)** Mammosphere assay of MCF10A shCTRL or shPRMT5 cells. n=3, error = s.e.m. *** $p< 0.005$ Student's t test.

Supplementary Experimental Procedures

Cell Culture

MCF7 and T47D cells were cultured in Dulbecco's Modified Eagle's Media (DMEM) (Sigma) supplemented with 10% foetal bovine serum (FBS) (Gibco) and 5mM glutamine (Sigma). MCF10A cells were cultured in DMEM/F-12 supplemented with 25% horse serum, 500ng/ml hydrocortisone (Sigma), 10μg/ml insulin (Sigma), 20ng/ml EGF (Miltenyi) and 100ng/ml cholera toxin (Sigma). SUM159 cells were cultured in Ham's F12 Nutrient Mixture (Thermo Scientific 21765-039) supplemented with 5% FBS, 1μg/ml hydrocortisone, 5μg/ml insulin and 10mM HEPES (Sigma). All cell lines were grown in 5U/ml penicillin and 1mg/ml streptomycin.

Generation of cell lines used in this study

All RNAi-mediated depletions were conducted via lentiviral infection of mammary/breast cancer cells and the generation of stable cell lines. For lentiviral production, low passage 293T cells were transfected with pLKO-shCTRL, -shPRMT5, -shPRMT1, -shFOXP1, pHIVzsGreen, pHIVzsGreen-PRMT5 or -FOXP1, or pHIVdTomato-MEP50 and appropriate packaging vectors, using PEI reagent (Park Scientific) in a 3:1 ratio of DNA:PEI for a minimum of six hours prior to addition of fresh media. Cells were cultured for 24hr before harvesting of viral supernatant. MCF7, T47D and MCF10A cells were transduced by centrifuging with viral supernatant containing 8μg/ml polybrene (Sigma) for 90 mins at 4000rpm. Cells were selected with 1μg/ml puromycin and only low passage numbers used for experiments. For doxycycline-inducible cell lines, MCF7 cells were first transduced with p201-doubleTet lentivirus (gift from K. Brennan) and CMV-Luciferase lentiviral particles (Amsbio), selected with 1mg/ml G418 (Sigma) and 2μg/ml blasticidin (Thermo Scientific) and then

infected with pLKO-Tet-ON-shCTRL or shPRMT5. For reconstitution of wild-type PRMT5 or catalytically inactive PRMT5, cells were first transfected with pLKO-shCTRL or shPRMT5 and selected with 1µg/ml puromycin. Surviving cells were then lentivirally infected with shRNA-resistant pHIVzsGreen-PRMT5 or PRMT5 (G367A/R368A) as described above and cells expressing the vector were isolated by flow cytometry. The shRNA sequences used in this study are as follows:

shCTRL: 5'-CCTAAGGTTAAGTCGCCCTCG-3'

shPRMT1(1): 5'-CCGGCAGTACAAAGACTACAA-3'

shPRMT1(2): 5'-TGAGCGTTCCTAGGCGGTTTC-3'

shPRMT5(1): 5'-GCGTTTCAAGAGGGAGTTCAT-3'

shPRMT5(2): 5'-AGGGACTGGAATACGCTAATT-3'

shFOXP1 (SIGMA TRCN0000015664): 5'-GCAGCAAGTTAGTGGATTAAA-3'

Mammosphere assay

MCF7, T47D and MCF10A cells were trypsinised and passed through a 25G needle to generate a single cell suspension. Cells were plated onto pHEMA (Sigma) -coated 6-well plates at a density of 3000 cells/well for MCF7 and 5000 cells/well for T47D, SUM159 and MCF10A cells and cultured at 37°C for five days. For MCF7, T47D and SUM159, cells were cultured in DMEM/F12 (Thermo Scientific 21041025) supplemented with B27 (Thermo Scientific 12587010), 20ng/ml EGF (Miltenyi) and 5U/ml penicillin and 1mg/ml streptomycin. For MCF10A, cells were cultured in DMEM/F12 supplemented with B27, 500ng/ml hydrocortisone, 10µg/ml insulin, 20ng/ml EGF, 100ng/ml cholera toxin and 5U/ml penicillin and 1mg/ml streptomycin. Mammospheres > 50µm were scored using a graticle. To assess self-renewal capabilities, mammospheres were harvested and disaggregated into single cell suspension as previously. Cells were cultured in pHEMA-coated 6-well plates for a further five

days prior to scoring. For mammosphere assays from xenografts, tumours were dissociated using the Miltenyi Tumour Cell Dissociation kit according to manufacturer's instructions. Cells were plated at 5000 cells/well and scored for both primary and secondary mammospheres and expressed as mammosphere forming unit (MFU). For drug treatment, cells were incubated with 5µM GSK591 or 2.5µM 4-OHT (Sigma).

Isolation of anoikis-resistant cells

MCF7 cells were plated on poly-HEMA coated 15cm dishes at a density of 900 cells/cm^2 and cultured in suspension overnight at 37°C. Anoikis-resistant cells were isolated by depletion of dead cells using the Miltenyi Dead Cell Removal kit according to manufacturer's instructions (Miltenyi 130-090-101).

Cell sorting and flow cytometry

Cells were trypsinised to form a single cell suspension. Cells were incubated with primary antibody as detailed in the table below prior to sorting or analysis.

Antibody	Cat No	Dilution
**ESA-FITC**	130-080-301	1:10
**CD24-PE**	130-095-952	1:10
**CD44-APC**	130-095-177	1:20
**Mouse IgG1-FITC**	130-092-213	1:10
**Mouse IgG1-PE**	130-092-212	1:10
**Mouse IgG1-APC**	130-098-214	1:20

Annexin-FITC (Biolegend 640905) staining was performed according to manufacturer's instructions. For PI staining, cells were harvested and fixed with ice cold 70% ethanol for 30

mins prior to incubation with 25 μg/ml propidium iodide solution (Sigma) containing 0.1 mg/ml RNAse A.

ALDEFLUOR assay

ALDEFLUOR assay (Stem Cell Technologies) was performed according to manufacturer's instructions using 2 x 10^5 cells per sample and incubation for 30 mins at 37°C. Where appropriate, cells were incubated with 5μM GSK591 or DMSO for a minimum of 24hr prior to harvest.

Xenograft studies and *in vivo* imaging

Animal experiments were conducted in accordance with United Kingdom Home Office regulations. For the limiting dilution assay, 5-7 week old female NSG (NOD/Scid/IL-2Rγnull) mice were injected with the appropriate number of MCF7 shCTRL, shPRMT5, shFOXP1, zsGreen/dTomato or zsPRMT5/MEP50 cells cells in a 1:1 ratio with matrigel (200μl total volume) (BD Biosciences). At the time of injection, a slow release oestrogen pellet (Innovative Research of America, NE-121) was subcutaneously implanted at the base of the tail. Tumour growth was monitored by caliper measurements. Two hours prior to harvest at the experimental endpoint, mice were injected with 100mg/kg BrdU (Sigma). Extracted tumours were weighed and imaged. Tumours were subsequently processed for protein extraction, immunohistochemistry (IHC) and mammosphere assay where appropriate. For *in vivo* depletion of PRMT5, female NSG mice were injected with 5 x 10^6 MCF7 Luc-Tet-ON-shCTRL or shPRMT5 cells as above. IVIS imaging and doxycycline-inducible knockdown initiated when tumours were of a palpable size. Mice were then maintained on diet consisting of doxycycline chow (TestDiet, T-5BQ8-1816629-203). For imaging, mice were injected IP with 150μg/g of luciferin (Promega) 15 mins prior to imaging. Images were captured on an

IVIS Spectrum. Tumours were harvested at the experimental endpoint and weighed prior to dissociation into single cells using the Miltenyi tumour dissociation kit according to manufacturer's instructions. Cells were then injected into female NSG mice in a limiting dilution assay.

Immunoblotting

Cell lysates were prepared by sonicating in lysis buffer (Tris-HCl pH 8.0, 150mM NaCl, 50mM, 1mM EDTA, 1% NP40, 1mM Na_3VO_4, 50mM NaF, 1mM β-glycero-phosphate, 100mM phenylmethylsulfonyl fluoride (PMSF), 10μg/ml leupeptin, 10μg/ml aprotinin), prior to incubation on ice and clarification by centrifugation. Proteins were separated by SDS-PAGE and transferred to nitrocellulose or PVDF. Membranes were blocked and incubated overnight in primary antibody at the dilutions indicated below. Membranes were washed and incubated with secondary antibody as indicated below.

Antibody	Company	Catalogue number	Dilution
**PRMT1**	CST	2449	1:1000
**PRMT5**	Millipore	07-405	1:1000
**MEP50**	CST	2823	1:1000
**SDMA**	CST	13222	1:1000
**SmD3**	Sigma-Aldrich	HPA001170	1:100
**FOXP1**	Kind gift from A. Banham		1:30
**GAPDH**	Santa Cruz	sc-32233	1:5000
**ACT**	Sigma-Aldrich	A2228	1:5000
**Tubulin**	Sigma-Aldrich	T6199	1:1000

**Anti-Rabbit IgG HRP**	DAKO	P0399	1:5000
**Anti-Mouse IgG HRP**	DAKO	P0447	1:5000

Immunohistochemistry

Tumours were fixed overnight in 10% neutralised buffered formalin (NBF) prior to embedding in paraffin. Sections were stained for BrdU (DAKO, M0744, 1:200) using the Mouse on Mouse (M.O.M. ™) Basic kit (Vector Labs) according to manufacturer's instructions and with haematoxylin (Sigma) and eosin (Sigma).

RNA extraction, qPCR and RT-PCR

RNA was extracted from cell pellets using the GenElute Mammalian Total RNA Isolation Kit (Sigma) according to manufacturer's instructions. RNA was reverse transcribed using the SensiFAST™ cDNA Synthesis Kit (Bioline) according to manufacturer's instructions. qPCR was performed using SYBR® Green PCR Master Mix (Thermo Scientific) according to manufacturer's instructions. RT-PCR for MDM4 was performed under the following conditions: 95°C: 5mins, followed by 95°C: 45s, 58°C: 30s, 72°C: 40s (43 cycles for MDM4 and 40 cycles for GAPDH) and 74°C: 4mins followed by cooling to 4°C. Water was used as a negative control. PCR products were visualised on a 3.5% agarose gel containing ethidium bromide. Primers used in this study are described below.

Gene	Forward primer (5'-3')	Reverse Primer (5'-3')
ACTB	CTCTTCCAGCCTTCCTTCCT	GGATGTCCACGTCACACTTC
PRMT5	GGCCAAGCAGGGGTTTGA	GGGCCGATTCTTAGCAGGTT
MEP50	CTCACTTGGGTTGGGGAGAG	CTCATTCTCATCTAGTTCCCACA
FOXP1	CCCTTCCCCTTCAACCTCTT	TTGCCTGTGGTTTCTTCTGC
OCT4	CGTGAAGCTGGAGAAGGAGA	TGCTCGAGTTCTTTCTGCAG
NANOG	CGTGAAGCTGGAGAAGGAGA	TGCTCGAGTTCTTTCTGCAG
SOX2	CCCCTGTGGTTACCTCTTCC	CCGGGGAGATACATGCTGAT
CRAT	CAGAAGATGGCTCCTGTGGG	AGCTCGGGTTTCTTCGTGTA
LOX	ACCCAGCCGACCAAGATATT	CTTCAGCCACTCTCCTCTGG
C1orf116	TTTCCGAGCACTGCCCATAA	TGCTGAGTGATGGTCTCCTC
SCIN	CGTGGAAGAAGGAAGTGAACC	TCATCATCACCTCCATCTGG
KRT81	CATGATCCAAAGGCTGACGG	CATCACTGAGGGCCGCCT
MAB21L3	GGAAGCTGGTGGAAAATGCA	TGTTTCCACAGCAACCCAAA
MPPED2	CTTCTCCACACAGGCGATTT	TGATTCCCAGCAATCACTATTTTA
RIMKLA	ACTGATGGACGGATGCAGAG	CCAACTGCTTGCCTTGTTCT
ADM	CGGCCCCAGGACATGAAG	TAGCGCTTGACTCGGATGC
CDKN1A	GAAGAAGGGCACCCTAGTTCTAC	ACCACATGGGACCCTCAC
ERBB4	CATCGAGAATGTGCTGGAGG	GGGACACTGAGTAACACATGC
COL21A1	GGGAAGTGATGAAGCAGAAACT	ATCCCCTTTCATCACGAGCT
COL3A1	CCCCCTGGAATCTGTGAATC	GCGAGTCCTCCTACTGCTAC
ABCA12	CTCGCACGAATTCTTGGCTT	GCTGTCACATAGTTCTCTTCGT
CLCA2	ACTTAACAGCTGGCTACGGA	CTCATCGAACACACCCCAAC

SLC4A10	AGAGGATGGAAGGGAGTCAC	TTCCTCGTCATCATCCTCGG
ABCC11	AGAACACCATCCCTCCACTG	GCTTTTTCAATCCCTCGCCT
NPY1R	AGGAGAAATACCAGCGGATCT	TTCCCTTGAACTGAACAATCC
MALRD1	AGGTATCAGAACAAGGGACCTG	ATTTCCGGACAAAGCTCAGA
MDM4	TGTGGTGGAGATCTTTTGGG	GCAGTGTGGGGATATCGT
GAPDH	GAAGGTGAAGGTCGGAGTC	GAAGATGGTGATGGGATTTC

RNA-sequencing

ESA$^+$CD24^{low}CD44$^+$ cells were isolated by flow cytometry from MCF7 shCTRL or shPRMT5 cells. Total RNA was extracted using the GenElute Mammalian Total RNA Isolation Kit and rRNA was depleted using the Low Input RiboMinus™ Eukaryote System v2 (Thermo Scientific) prior to sequencing on the Illumina Hiseq. Sequenced reads were mapped to build hg19 of the human genome from the University of California Santa Cruz (UCSC) genome database using TopHat version 2.0.12 (Kim et al., 2013) and aligned with Bowtie (Langmead et al., 2009) using default parameters and by supplying the gene model with annotations from Ensembl. Differential gene expression was determined using Cuffdiff 2.2.1 with a threshold of FDR < 0.05 and fold change > 1.5. Enriched Gene Ontology terms and KEGG pathways were identified using DAVID (david.ncifcrf.gov) (Huang et al., 2009). Experiment was performed using three independent replicates of shCTRL and shPRMT5 ESA$^+$CD24^{low}CD44$^+$ cells.

Isolation of tumour-derived patient cells

Fresh tumour samples were collected from resected breast tumours from patients at the Queen Elizabeth Hospital, Birmingham or in collaboration with Barts Cancer Institute with informed consent. Samples were chopped into 0.5cm^3 chunks and dissociated by incubating overnight

with 0.5mg/ml collagenase (Sigma-Aldrich) and 0.5mg/ml hyaluronidase (Sigma) in RPMI supplemented with 5% FBS, 5µg/ml amphotericin (Sigma) and 5U/ml penicillin and 1mg/ml streptomycin. Cells were isolated by centrifugation and incubated in red blood cell lysis buffer (155mM NH_4Cl, 10mM $KHCO_3$, 0.1mM EDTA) at room temperature for 10 min. Cells were collected by centrifugation at 800rpm for 10 min. For mammosphere assays, 10 000 cells/well of a poly-HEMA coated 6-well plate were cultured ± 5µM GSK591 or DMSO for the duration of the assay.

Chromatin Immunoprecipitation

MCF7 shCTRL or shPRMT5 cells were fixed in 1% formaldehyde before being quenched with 0.125M glycine. Cells were washed once with PBS and nuclei isolated by incubating with lysis buffer (10mM Tris-HCl pH 8.0, 10mM NaCl, 0.2% NP40, 10mM sodium butyrate, 50µg/ml PMSF, 1µg/ml leupeptin) and centrifugation. Nuclei were lysed (50mM Tris-HCl pH 8.1, 10mM EDTA, 1% SDS, 10mM sodium butyrate, 50µg/ml PMSF, 1µg/ml leupeptin) and chromatin sonicated to produce DNA fragments of between 300bp and 500bp. Chromatin was precleared using Rabbit IgG (Millipore, 12-370; 1 µg per 10^6 cells) and protein G-agarose (Roche, 11719416001). ChIP was performed using chromatin equivalent to 5 x 10^6 cells. Chromatin was incubated overnight at 4^oC with 5µg PRMT5 (Millipore, 07-405), H3K4me3 (Abcam, ab8580), Set1 (Abcam ab70378), H4R3me2s (Abcam ab5823), 10µl H3R2me2s (gift from E. Guccione) or the equivalent amount of Rabbit IgG (Millipore, 12-370), followed by 3hr incubation with protein G beads. Beads were collected and washed twice with wash buffer 1 (20 mM Tris-HCl pH 8.1, 50 mM NaCl, 2 mM EDTA, 1% Triton X-100, 0.1% SDS), once with wash buffer 2 (10mM Tris-HCL pH 8.1, 250mM LiCl, 1mM EDTA, 1% NP-40, 1% deoxycholic acid), twice with Tris-EDTA (pH 8) and eluted in 100mM $NaHC0_3$, 1% SDS. Immunoprecipitated DNA was reverse crosslinked and treated with proteinase-K prior to

purification by phenol/chloroform and ethanol precipitation. For anoikis-resistant cells, ChIP was performed using chromatin equivalent to 2 x 10^5 cells. Chromatin was incubated overnight at 4°C with 1µg H3K4me3 (Abcam, ab8580) or Rabbit IgG (Millipore, 12-370) and isolation of DNA as described earlier. Associated DNA was quantified using the primers below.

Gene	Forward primer (5'-3')	Reverse Primer (5'-3')
FOXP1 **(-1683)**	ccttagcagcctccctttct	gggtggcataagactatcgc
FOXP1 **(-1442)**	gacagtaagcctctcccctc	tcccaaaccagcatttccag
FOXP1 **(-1098)**	aggctcttagtttattccttcga	gctctcccagtgatcagtca
FOXP1 **(-838)**	agtggctctgacaagggg	ttctcccctgtttctcggaa
FOXP1 **(-627)**	gacgggagagagggagaaag	gtgacagttgaaggagagcg
FOXP1 **(-397)**	ccccatttcccatctcctga	agggatctggcaggaaagac
FOXP1 **(-187)**	cccattctttggcaattgcg	gtccctgacgtcacatccac
FOXP1 **(+396)**	caccgagagaccatttgcag	gcttgggaaaaagtggagca
MYOD1 **(-585)**	cacttcaactctcggggtct	ccgaaagaggctgagagaca

Cell proliferation assay

Cells were plated in triplicate at a density of 20 000 cells/well in 24-well plates. Cell proliferation was determined by counting on a haemocytometer with trypan blue exclusion.
